# Fc effector of anti-Aβ antibody induces synapse loss and cognitive deficits in Alzheimer’s disease-like mouse model

**DOI:** 10.1038/s41392-022-01273-8

**Published:** 2023-01-25

**Authors:** Xiao-ying Sun, Xiao-lin Yu, Jie Zhu, Ling-jie Li, Lun Zhang, Ya-ru Huang, Dong-qun Liu, Mei Ji, Xun Sun, Ling-xiao Zhang, Wei-wei Zhou, Dongming Zhang, Jianwei Jiao, Rui-tian Liu

**Affiliations:** 1 State Key Laboratory of Biochemical Engineering, Institute of Process Engineering, Chinese Academy of Sciences, Beijing, 100190 China; 2 School of Chemistry and Chemical Engineering, University of Chinese Academy of Science, Beijing, 100049 China; 3 Innovation Academy for Green Manufacture Institute, Chinese Academy of Sciences, Beijing, 100190 China; 4 State Key Laboratory of Stem Cell and Reproductive Biology, Institute of Zoology, Chinese Academy of Sciences, Beijing, 100101 China

**Keywords:** Neuroimmunology, Neurological disorders

## Abstract

Passive immunotherapy is one of the most promising interventions for Alzheimer’s disease (AD). However, almost all immune-modulating strategies fail in clinical trials with unclear causes although they attenuate neuropathology and cognitive deficits in AD animal models. Here, we showed that Aβ-targeting antibodies including their lgG1 and lgG4 subtypes induced microglial engulfment of neuronal synapses by activating CR3 or FcγRIIb via the complex of Aβ, antibody, and complement. Notably, anti-Aβ antibodies without Fc fragment, or with blockage of CR3 or FcγRIIb, did not exert these adverse effects. Consistently, Aβ-targeting antibodies, but not their Fab fragments, significantly induced acute microglial synapse removal and rapidly exacerbated cognitive deficits and neuroinflammation in APP/PS1 mice post-treatment, whereas the memory impairments in mice were gradually rescued thereafter. Since the recovery rate of synapses in humans is much lower than that in mice, our findings may clarify the variances in the preclinical and clinical studies assessing AD immunotherapies. Therefore, Aβ-targeting antibodies lack of Fc fragment, or with reduced Fc effector function, may not induce microglial synaptic pruning, providing a safer and more efficient therapeutic alternative for passive immunotherapy for AD.

## Introduction

Microglia, the predominant immune cells in the central nervous system (CNS), play crucial physiological roles by clearing pathogens and dying neurons, and prune excess neuronal synapses, through the recognition of complement C1q and C3 tagged on the synapses via microglial complement receptor 3 (CR3) during brain development.^1–3^ Synapse loss, a critical hallmark in Alzheimer’s disease (AD) and numerous other neurodegenerative disorders, correlates strongly with cognitive deficits.^4–7^ In the progress of AD, oligomeric β-amyloid (Aβ) prefers to bind to synapses, triggers microglia to excessively eliminate synapses via complement-dependent pathway, which contributes to synapse loss and cognitive deficits.^8,9^

To attenuate the deleterious effects of Aβ, immunotherapy, one of the most promising interventions for AD, has been extensively explored with long-running endeavor. However, almost all of the tested antibodies fail in clinical trials because of low efficacy or side effects such as cerebral inflammation, hemorrhage, and amyloid-related imaging abnormalities-edema (ARIA-E).^10,11^ To overcome the side effects of immunotherapies, antibodies targeting Aβ N-terminus (such as bapineuzumab), or IgG4 and IgG2 subtypes such as crenezumab and ponezumab were applied in clinical trials.^12–14^ However, these antibodies still failed to rescue cognitive deficits in AD patients although some side effects significantly reduced. Recently, antibodies targeting Aβ aggregates such as aducanumab and lecanemab showed some beneficial effects in rescuing cognitive deficits, but they still exhibited low efficacy.^15,16^ Thus, the underlying mechanism of the failures and low efficacy in clinical trials should be urgently investigated for the development of efficient immunotherapies.

The therapeutic prospects of antibodies need to be balanced by potential responsibilities associated with immune modulation, particularly in CNS.^17^ Notably, the classical complement proteins may bind to the Fc fragment of antibody in the antibody-antigen complexes when antibodies are applied for the treatment of CNS diseases, inducing the cascade of complement activation.^18,19^ Based on the previous findings,^8,20,21^ we speculate that the Fc effector of therapeutic antibodies against Aβ triggers activation of the complement components to induce synapse engulfment by microglia in brain likely via complement-dependent microglial synapse pruning pathway.

In the present study, we used neuron-microglia cocultures and APP/PS1 transgenic mouse model to validate our hypothesis. We show that an Aβ-targeting antibody remarkably amplified microglial synapse pruning both in neuron-microglia cocultures and in APP/PS1 mice via the complement-dependent pathway, resulting in significant synapse loss and cognitive deficits in APP/PS1 mice, which may reveal the possibility of the clinical trial failure of AD immunotherapy.

## Results

### Fc effector of anti-Aβ antibody mediates acute synapse loss and cognitive deficits in APP/PS1 mice

**Fig. 1:**
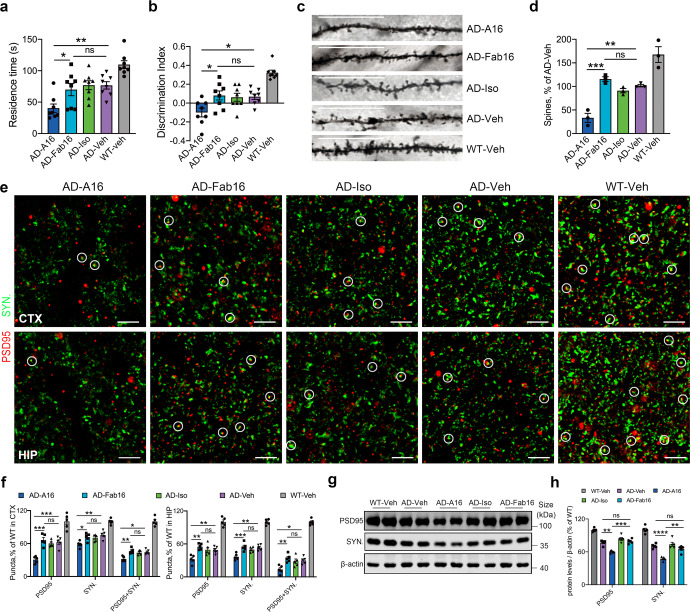
Full effector antibody A16 robustly mediates synapse loss and cognitive deficits in APP/PS1 mice at 24-48 h post-treatment. **a** The time spent by APP/PS1 or WT mice in the novel arm of Y-maze at 24 h post-treatment with A16, Fab16, isotype control antibody, or PBS (*n* = 8 mice). **b** Discrimination index of the mice in novel object recognition test (*n* = 8 mice). **c** Representative images of Golgi-stained dendrites from the cortical neurons of APP/PS1 or WT mice treated with A16, Fab16, isotype control antibody, or PBS, at 48 h post-injection. Scale bar, 50 μm. **d** Quantification of dendritic spine density in **c** (*n* = 3 mice). **e** Immunolabeling of PSD95 (red) and synaptophysin (green) in the cortex and hippocampus of APP/PS1 or WT mice treated with A16, Fab16, isotype control antibody, or PBS, at 48 h post-injection. White circles indicate the colocalized PSD95 with synaptophysin puncta. Scale bar, 2.5 μm. **f** Quantification of synaptic puncta or their apposition in **e** (*n* = 5 mice). **g** Western blot analysis of PSD95 and synaptophysin in the brains of APP/PS1 or WT mice treated with A16, Fab16, isotype control antibody, or PBS, at 48 h post-injection. **h** Quantitation of PSD95 and synaptophysin expression in **g** (*n* = 5 mice). Data are expressed as mean ± s.e.m. and were analyzed by one-way ANOVA with Tukey’s test. *P < 0.05, **P < 0.01, ***P < 0.001, ****P < 0.0001; ns not significant

To investigate the effects of antibody effector function on microglial synapse engulfment, we generated a murine monoclonal antibody against Aβ 1-16 with an IgG1 backbone (A16), while its effector-less F(ab’)_2_ antibody (Fab16) was obtained by using ficin digestion to remove the Fc fragment. A16 and Fab16 showed scant differences in their specificity and affinity for Aβ, and inhibitory effects on Aβ aggregation and cytotoxicity (Supplementary Fig. 1). APP/PS1 mice were intracerebroventricular (ICV) administered A16, Fab16, or isotype control antibodies. The cognitive performance in mice was then assessed using Y-maze and novel object recognition (NOR) tests at 24 h post-injection. A16 induced cognitive deficits in APP/PS1 mice, with reduced residence time in the novel arm of Y-maze (Fig. 1a) and decreased discrimination index in the NOR test (Fig. 1b). Consistently, A16, but not Fab16 or IgG control, markedly reduced the dendritic spine levels in the brains of APP/PS1 mice (Fig. 1c, d). We further measured the levels of two neuronal synaptic marker proteins by immunohistochemistry, one is presynaptic protein synaptophysin, and the other is post synaptic density protein-95 (PSD95), which is a member of the membrane-associated guanylate kinase (MAGUK) family. The colocalization of synaptophysin and PSD95 represent structural integrity of synapses. Compared with that of vehicle-treated APP/PS1 mice, a significant decrease in synapse number was observed in A16-, but not in Fab16- or IgG control-treated APP/PS1 mice (Fig. 1e, f and Supplementary Movie 1). This result was confirmed by detecting the levels of the synapse marker proteins using western blotting (Fig. 1g, h).

**Fig. 2:**
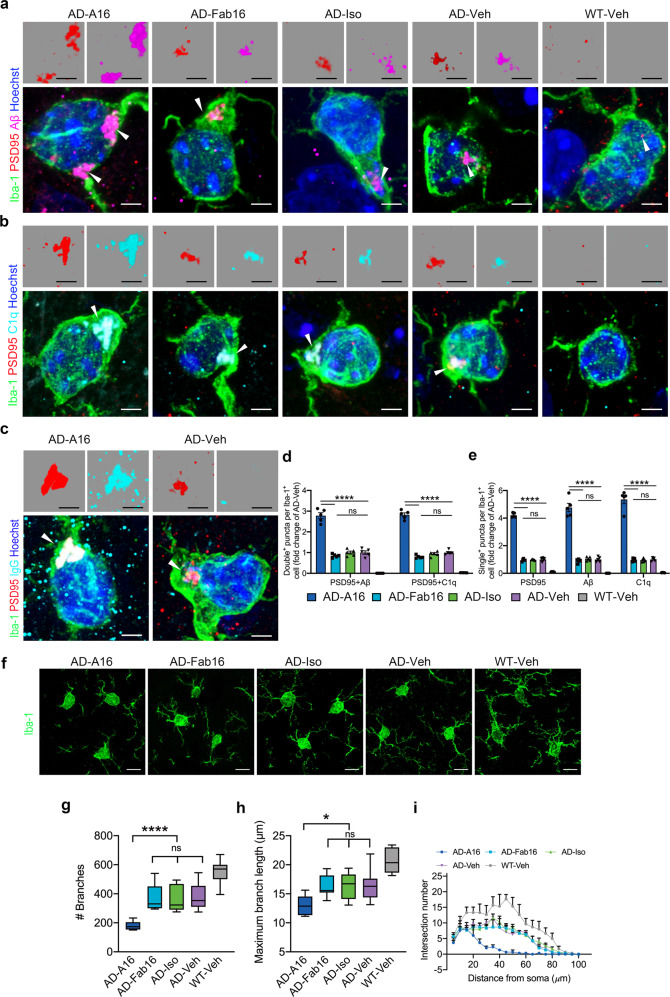
Full effector antibody A16 significantly promotes microglial engulfment of synapses in APP/PS1 mice at 48 h post-treatment. **a** Representative images of the engulfed PSD95 (red) and Aβ (magenta) puncta within Iba-1^+^ (green) microglial cells in the brains of APP/PS1 or WT mice treated with A16, Fab16, isotype control antibody, or PBS, at 48 h post-injection. Scale bar, 3 μm. **b** Representative images of the engulfed PSD95 (red) and C1q (cyan) puncta within Iba-1^+^ (green) microglial cells in the brains of APP/PS1 or WT mice treated with A16, Fab16, isotype control antibody, or PBS, at 48 h post-injection. Scale bar, 3 μm. **c** Representative images of the engulfed PSD95 (red) and IgG (cyan) puncta within Iba-1^+^ (green) microglial cells in the brains of APP/PS1 mice treated with A16 or PBS, at 48 h post-injection. Scale bar, 3 μm. **d** Quantification of the colocalized PSD95 and Aβ puncta, and PSD95 and C1q puncta, per Iba-1^+^ microglial cell (*n* = 5 mice). **e** Quantification of PSD95, Aβ, and C1q puncta per Iba-1^+^ microglial cell (*n* = 5 mice). **f** Representative images of Iba-1 (green)-stained microglial cells in the brains of APP/PS1 or WT mice treated with A16, Fab16, isotype control antibody, or PBS, at 48 h post-injection. Scale bar, 10 μm. Skeleton analysis (**g**, **h**) and Sholl analysis (**i**) of microglial cells in **f** (*n* = 5 mice). Arrows indicate the engulfed inputs in microglia in (**a**–**c**). Data are expressed as mean ± s.e.m. (**d**, **e**, **i**) or shown as boxplots (**g**, **h**). For boxplots, the central band displays the median, the boxes depict values between lower and upper quartile, and the whiskers represent the minimum and maximum values. One-way ANOVA with Tukey’s test was performed to determine significance. *P < 0.05, ****P < 0.0001; ns not significant

To determine whether A16 antibody-induced synapse loss was associated with microglial synapse engulfment, we quantified PSD95, Aβ, and C1q puncta in Iba-1-immunoreactive microglia in the brains of APP/PS1 mice. A significant increase and colocalization of PSD95, Aβ, and C1q was observed in the microglia of APP/PS1 mice treated with A16, but not in those of mice treated with Fab16 or IgG control (Fig. 2a, b, d, e, Supplementary Fig. 2, and Supplementary Movie 2). We also detected a significant increase and colocalization of PSD95 and IgG in the microglia of APP/PS1 mice treated with A16 (Fig. 2c). Treatment with A16 significantly decreased the number of ramified microglia (Fig. 2f). The skeleton analysis of microglia morphologies showed fewer and shorter processes per microglial cell in APP/PS1 mice treated with A16, compared with those of mice treated with Fab16 or vehicle (Fig. 2g, h). Consistently, Sholl analysis demonstrated that microglia became de-ramified in A16-treated APP/PS1 mice, but not in Fab16- or vehicle-treated mice (Fig. 2i). These findings revealed that treatment with A16 markedly promoted synapse phagocytosis by microglia, and caused synapse loss in APP/PS1 mice at 48 h post-injection, resulting in rapidly cognitive decline. By contrast, A16 treatment in WT mice did not induce cognitive impairment (Supplementary Fig. 3a, b), synapse loss (Supplementary Fig. 3c–f) or synaptic engulfment by microglia (Supplementary Fig. 3g, h), suggesting that A16-triggered microglial synapse elimination was Aβ-dependent.

### Full effector antibody reduces Aβ burden but promotes gliosis in the brains of APP/PS1 mice at 48 h post-treatment

**Fig. 3:**
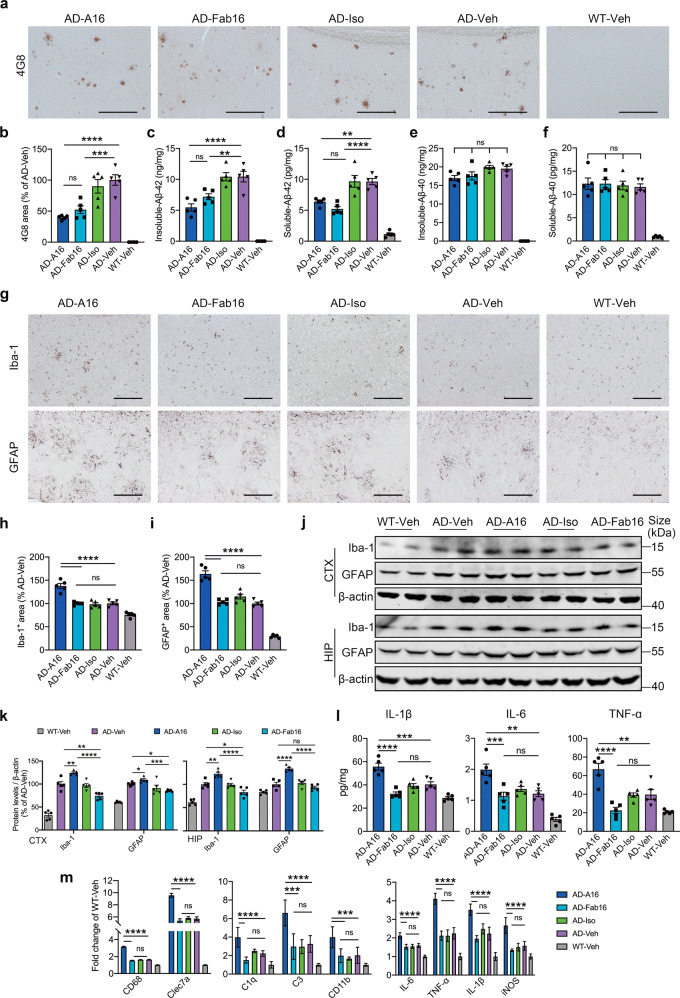
Full effector antibody A16 reduces Aβ burden, but enhances inflammation in the brains of APP/PS1 mice at 48 h post-treatment. **a** Detection of Aβ plaques by 4G8 immunolabeling in the brains of APP/PS1 or WT mice treated with A16, Fab16, isotype control antibody, or PBS, at 48 h post-injection. Scale bar, 200 μm. **b** Quantification of 4G8-labeled area in **a** (*n* = 5 mice). Levels of insoluble Aβ42 (**c**), soluble Aβ42 (**d**), insoluble Aβ40 (**e**), and soluble Aβ40 (**f**) in brain lysates of APP/PS1 or WT mice treated with A16, Fab16, isotype control antibody, or PBS, at 48 h post-injection (*n* = 5 mice). **g** Detection of microglia and astrocytes by immunolabeling Iba-1 and GFAP in the brains of APP/PS1 or WT mice treated with A16, Fab16, isotype control antibody, or PBS, at 48 h post-injection. Scale bar, 200 μm. **h** Quantification of Iba-1-labeled area in **g** (*n* = 5 mice). **i** Quantification of GFAP-labeled area in **g** (*n* = 5 mice). **j** Western blot analysis of Iba-1 and GFAP in the cortex and hippocampus of APP/PS1 or WT mice treated with A16, Fab16, isotype control antibody, or PBS, at 48 h post-injection. **k** Quantitation of Iba-1 and GFAP expression in **j** (*n* = 5 mice). **l** The levels of IL-1β, IL-6, and TNF-α in the brain lysates of APP/PS1 or WT mice treated with A16, Fab16, isotype control antibody, or PBS, at 48 h post-injection (*n* = 5 mice). **m** The expression signatures of inflammation-related gene (CD68, Clec7a), complement-related gene (C1q, C3, CD11b), and inflammatory factor-related gene (IL-6, TNF-α, IL-1β, iNOS) in microglial cells of APP/PS1 mice were analyzed by qPCR (*n* = 6 mice). Data are expressed as mean ± s.e.m. and were analyzed by one-way ANOVA with Tukey’s test. *P < 0.05, **P < 0.01, ***P < 0.001, ****P < 0.0001; ns not significant

To explore the effect of full effector antibody on the neuropathology of APP/PS1 mice, we evaluated Aβ burden and gliosis in the brains of APP/PS1 mice. Treatment with A16 and Fab16 significantly reduced the plaque burden and Aβ42 levels in the brains of APP/PS1 mice at 48 h post-injection (Fig. 3a–d), while the levels of Aβ40 were not affected at this time point (Fig. 3e, f). Moreover, treatment with A16, but not Fab16, significantly increased microgliosis and astrocytosis in the brains of APP/PS1 mice (Fig. 3g–i). Western blot analysis consistently showed increased levels of Iba-1 and GFAP in the brains of APP/PS1 mice treated with A16 (Fig. 3j, k). The levels of IL-1β, IL-6, and TNF-α in A16-treated APP/PS1 mice were higher than those of APP/PS1 mice treated with Fab16 and those of mice in other control groups (Fig. 3l). Furthermore, to assess the impact of A16 or Fab16 on microglial activation states, we measured inflammation- and complement-related gene expression signatures in microglial cells of APP/PS1 mice. The results showed that the inflammation-related transcripts, such as CD68, Clec7a, and iNOS, inflammatory factor transcripts such as IL-1β, IL-6 and TNF-α, and complement-pathway-related transcripts such as C1q, C3, and CD11b were significantly upregulated in microglial cells of APP/PS1 mice treated with A16 relative to Fab16 and vehicle (Fig. 3m). The immunoreactivity of C1q (Supplementary Fig. 4a, b) and the levels of C1q protein (Supplementary Fig. 4c, d) were significantly elevated in the brains of APP/PS1 mice treated with A16, but not in those treated with Fab16 or IgG isotype control. However, A16 treatment in WT mice did not induce gliosis and inflammatory cytokine release (Supplementary Fig. 3i–k, m, n), also failed to activate the classical complement cascade (Supplementary Fig. 3l–n).

To detect the potential consequences of antibody therapy in APP/PS1 mice at different age, we performed A16 treatment in 3- and 5-month-old APP/PS1 mice before Aβ deposition development in brains, and 10-month-old APP/PS1 mice with significant plaque load, synapse loss and cognitive impairments. In our study, 3-month-old APP/PS1 mice showed no difference in cognitive performance, synaptic and C1q levels compared with WT mice (Supplementary Fig. 5), and A16 treatment did not induce cognitive decline (Supplementary Fig. 5a, b), neuroinflammation (Supplementary Fig. 6) and microglial synapse engulfment (Supplementary Fig. 7) in 3-month-old APP/PS1 mice when relative to APP/PS1 controls. However, 5-month-old APP/PS1 controls exhibited some but without statistical difference in cognitive deficits and synapse loss compared with WT mice (Supplementary Fig. 8a–e, g). Also, no significant difference in cognitive performance (Supplementary Fig. 8a, b), synapse number (Supplementary Fig. 8c–e, g), and Aβ levels (Supplementary Fig. 9a–f) was observed in 5-month-old APP/PS1 mice treated with A16 relative to vehicle control. However, compared with the WT-Veh group, A16-treated APP/PS1 mice at 5 months of age showed a significant decrease in cognitive ability and synaptic levels (Supplementary Fig. 8a–e, g). When compared with 5-month-old APP/PS1 controls, A16 treatment resulted in a remarkable increase in C1q level (Supplementary Fig. 8f, h), neuroinflammation (Supplementary Fig. 9g–i) and microglial phagocytosis of PSD95 (Supplementary Fig. 10). For 10-month-old APP/PS1 mice, A16 treatment triggered further synapse loss (Supplementary Fig. 11c–e, g), significant microglial synapse engulfment (Supplementary Fig. 12), decreased Aβ42 levels (Supplementary Fig. 13a–f), increased C1q level (Supplementary Fig. 11f, h) and neuroinflammation (Supplementary Fig. 13g–i) in APP/PS1 brains. However, we failed to find further remarkable cognitive decline in A16-treated APP/PS1 mice (Supplementary Fig. 11a, b), which may be due to the limitation of Y-maze and NOR measurements.

To verify the reliability and repeatability of the results, we further applied 6E10 (Signet Laboratories’ monoclonal anti-Aβ antibody), a conventional benchmark antibody, to 6-month-old APP/PS1 mice via ICV injection. There was no difference in the binding ability to Aβ between 6E10 and A16 (Supplementary Fig. 1h). 6E10, consistent with A16, caused cognitive deficits (Supplementary Fig. 14a, b), synapse loss (Supplementary Fig. 14c–e, g), complement activation (Supplementary Fig. 14f, h), neuroinflammation (Supplementary Fig. 15g–j) and microglial phagocytosis of PSD95 (Supplementary Fig. 16) in APP/PS1 mice, 6E10 treatment also lowered plaques and Aβ42 levels (Supplementary Fig. 15a–d), but not Aβ40 (Supplementary Fig. 15e, f) in APP/PS1 brains.

### Antibody-mediated microglial engulfment of synapse requires the participation of Aβ rather than amyloid precursor protein

Aβ-targeting antibody A16 is able to bind to amyloid precursor protein (APP) C-terminal and C99, but not neuronal marker proteins MAP2, PSD95 or synaptophysin (Supplementary Fig. 1g). We further measured the levels of Aβ and APP in the brain lysates of APP/PS1 mice at 3, 5, 6, 7, 10 months of age. The levels of APP in APP/PS1 mouse brains are constant but Aβ levels increase within 3-10 months of age (Supplementary Fig. 17), whereas antibody-induced microglial synapse removal was observed only at 5-10 months of age. These findings suggested that A16-mediated microglial engulfment of synapse required the participation of Aβ rather than APP.

### Full effector antibody mediates synapse engulfment by microglia in vitro

**Fig. 4:**
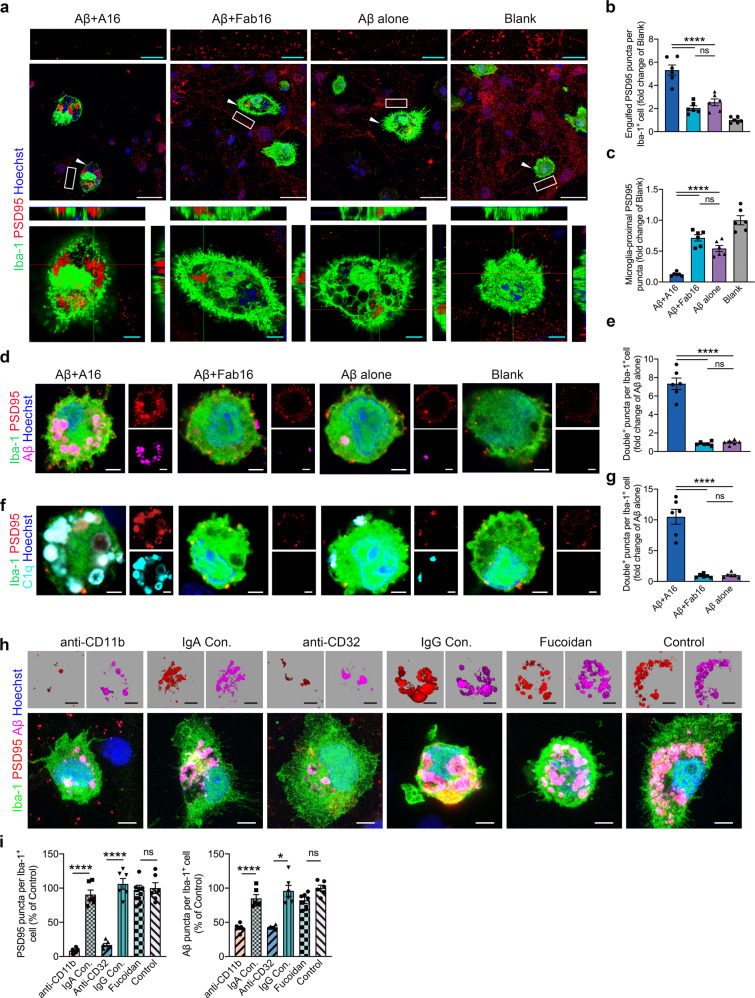
Full effector antibody A16 but not the effector-less antibody Fab16 induces microglial engulfment of synapses in vitro. **a** Immunolabeling of PSD95 (red) and Iba-1 (green) in neuron-microglia cocultures treated with A16 or Fab16 in the presence or absence of AβOs. Scale bars: cyan, 5 μm; white: 25 μm. Arrows indicate the enlarged microglia cells. Rectangles indicate microglia-proximal PSD95 puncta. **b** Quantification of engulfed PSD95 puncta in Iba-1^+^ microglial cells in **a** (*n* = 6). **c** Quantification of microglia-proximal PSD95 density (<15 μm from microglia) in a (*n* = 6). **d** Immunolabeling of PSD95 (red) and Aβ (magenta) in Iba-1^+^ (green) microglial cells in cocultures. Scale bar, 2.5 μm. **e** Quantification of colocalized PSD95 and Aβ puncta per Iba-1^+^ microglial cell in **d** (*n* = 6). **f** Immunolabeling of PSD95 (red) and C1q (cyan) in Iba-1^+^ (green) microglial cells in cocultures. Scale bar, 2.5 μm. **g** Quantification of colocalized PSD95 and C1q puncta per Iba-1^+^ microglial cell in **f** (*n* = 6). **h** Immunolabeling of engulfed PSD95 (red) and Aβ (magenta) within Iba-1^+^ (green) microglial cell in neuron-microglia cocultures treated with A16 in presence of AβOs. Microglia cells were pretreated with anti-CD11b functional antibody or its isotype control IgA, anti-CD32 functional antibody or its isotype control IgG, or fucoidan, respectively. Scale bar, 5 μm. **i** Quantification of PSD95 and Aβ puncta engulfed by Iba-1^+^ microglial cells in **h** (*n* = 6). All experiments in **b**, **c**, **e**, **g**, and **i** were performed as biological replicates over three independent experiments. Data are expressed as mean ± s.e.m. and were analyzed by one-way ANOVA with Tukey’s test. *P < 0.05, ****P < 0.0001; ns not significant

To further confirm the effects of full effector antibody on microglia-mediated synapse engulfment, we added A16 to the neuron-microglia cocultures in the presence of 500 nM Aβ oligomers (AβOs, Supplementary Fig. 18). A16 induced a significantly higher number of internalized synapses in microglia compared with that induced by Fab16 (Fig. 4a, b and Supplementary Fig. 19). We consistently observed the disappearance of numerous neuronal synapses around microglia (Fig. 4a, c and Supplementary Fig. 20a, b). PSD95 levels in A16-treated cocultured neurons, determined using western-blotting, were significantly decreased (Supplementary Fig. 20c, d). We also detected significantly increased colocalization of PSD95, synaptophysin, and Aβ in the microglial cells treated with A16 but not with Fab16 (Fig. 4d, e and Supplementary Fig. 20g, h). Previous studies have shown that AβOs promote microglial engulfment of synapses,^8^ this effect was robustly exacerbated by A16 (Supplementary Fig. 20i). However, A16 alone, without Aβ, did not induce microglial synapse engulfment (Supplementary Fig. 21).

It is reported that C1q is a key mediator of AβO-induced synaptic loss.^8^ Upon AβO challenge, we observed significantly upregulated C1q expression and increased levels of C1q and Aβ in cultured microglial cells (Supplementary Fig. 22a–c), verifying microglia as an important source of C1q. In microglial cultures in the presence of AβOs, A16 induced significantly higher C1q expression and higher levels of IL-1β, TNF-α, and IL-6 than Fab16 (Supplementary Fig. 22c, d). Because C1q is the initiating protein of the classical complement cascade, we further assessed whether the classical complement cascade was involved in A16-mediated synapse engulfment. We observed increased quantities of C1q and C3 puncta, which mainly overlapped with PSD95, Aβ, A16, and lysosome markers in A16-treated microglial cells (Fig. 4f, g and Supplementary Fig. 20e–h, j, k). By contrast, Fab16 did not induce the accumulation of synaptic proteins and complement components in microglia.

### CR3 and FcγRIIb participate in microglia-mediated synapse loss

To further investigate which receptors are involved in A16-triggered synaptic engulfment by microglia, we used an anti-CD11b antibody to block CR3, and an anti-CD32 antibody to block FcγRIIb, on microglia. Both antibodies, but not their isotype control antibodies, significantly prevented synapse engulfment, as well as AβO phagocytosis by microglia (Fig. 4h, i). Consistently, depletion of C1qa, C3, CD11b, and FcγRIIb abrogated microglial phagocytic capacity for synapses, respectively (Supplementary Fig. 23a–c). However, fucoidan, a general ligand and antagonist of scavenger receptors, did not inhibit microglial synapse uptake (Fig. 4h, i). These results suggest that both CR3 and FcγRIIb, but not scavenger receptors, participate in antibody-mediated microglial engulfment of synapses.

### Treatment with IgG4 antibody induces acute microglia-mediated synapse loss in APP/PS1 mice

A16I4, an IgG4 subtype Aβ-targeting antibody, was obtained via genetically engineered modification of A16. Treatment with A16I4 significantly increased intracellular levels of PSD95 and synaptophysin, which colocalized fully with Aβ and C3, but not C1q, in microglial cells from the coculture system (Supplementary Figs. 24, 25). These results suggest that A16I4 promoted microglial phagocytosis of Aβ and synapses by activating the complement system via the alternative or lectin complement pathway, rather than the classical pathway.^22,23^ Consistently, a significant increase and colocalization of PSD95, Aβ and C3 were observed in the microglia of APP/PS1 mice at 48 h post-intravenous injection with A16I4 (Supplementary Fig. 26a–e and Supplementary Fig. 27). Moreover, treatment with A16I4 resulted in a markedly decrease of ramified microglia by the skeleton and Sholl analysis (Supplementary Fig. 26f–i). These results indicate that IgG4 subtype Aβ-targeting antibody also promoted synapse engulfment by microglia and resulted in synapse loss.

### Full effector antibody rescues cognitive deficits in APP/PS1 mice at 2-week post 2nd antibody treatment

**Fig. 5:**
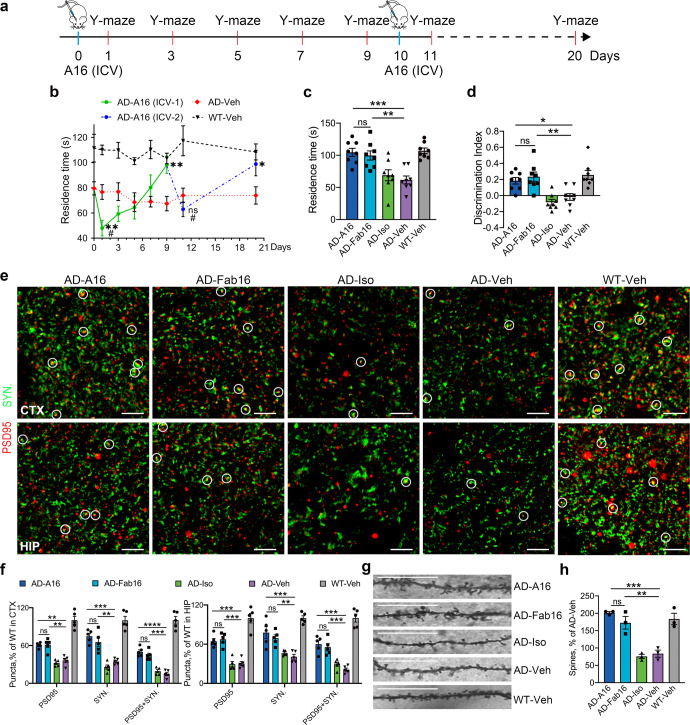
Full effector antibody A16 rescues memory deficits and synapse loss in APP/PS1 mice at 2-week post 2nd antibody treatment. **a** Schematic representation of treatment regimen with A16 and experimental design. **b** APP/PS1 mice were administered A16 or vehicle on Days 0 and 10, and underwent Y-maze test on days 1, 3, 5, 7, 9, 11, and 20. The time spent in the novel arm was scored. *n* = 8 mice, AD-A16 versus AD-Veh: Day 1, ^**^P = 0.0052; Day 9, ^**^P = 0.0049; Day 11, ns, *P* = 0.1958; Day 20, ^*^P = 0.0470; A16 at Day 0 versus A16 at Day 1, ^#^*P* = 0.0112; A16 at Day 9 versus A16 at Day 11, ^#^*P* = 0.0109. **c** APP/PS1 or WT mice were treated with A16, Fab16, isotype control antibody, or PBS twice biweekly, and their cognitive performance was evaluated using Y-maze test at 2-week post 2nd antibody treatment. The time spent in the novel arm of Y-maze was scored (*n* = 8 mice). **d** Discrimination index of the mice in novel object recognition test (*n* = 8 mice). **e** Immunolabeling of colocalized PSD95 (red) and synaptophysin (green) puncta in the cortex and hippocampus of APP/PS1 or WT mice treated with A16, Fab16, isotype control antibody, or PBS, at 2-week post 2nd antibody treatment. White circles indicate the colocalized PSD95 with synaptophysin puncta. Scale bar, 2.5 μm. **f** Quantification of synaptic puncta or their apposition in **e** (*n* = 5 mice). **g** Representative images of Golgi-stained dendrites from the cortex neurons of APP/PS1 or WT mice treated with A16, Fab16, isotype control antibody, or PBS, at 2-week post 2nd antibody treatment. Scale bar, 50 μm. **h** Quantification of dendritic spine density in **g** (*n* = 3 mice). Data are expressed as mean ± s.e.m. and were analyzed by one-way ANOVA with Tukey’s test (**c** and **d**, **f**, **h**) or student t-test (**b**). *P < 0.05, **P < 0.01, ***P < 0.001, ****P < 0.0001; ns not significant

Contrary to our results obtained at 24-48 h post-treatment, numerous studies have shown that Aβ-targeting antibodies can attenuate cognitive deficits and neuroinflammation at several days post-treatment.^24^ To track changes in the cognitive performance of 6-month-old APP/PS1 mice treated with Aβ-targeting antibodies, we tested the memory function of APP/PS1 mice using Y-maze at 1, 3, 5, 7, and 9 days post-treatment (Fig. 5a). Consistent with our previous findings, APP/PS1 mice treated with A16 spent considerably less time in the novel arm compared with APP/PS1 control mice at one day post-injection, indicating that the memory function of APP/PS1 mice was rapidly impaired by A16 treatment (Fig. 5b). Then the memory of A16-treated mice partly recovered on day 3 post-injection, which reached a cognition level similar as that of APP/PS1 control mice on day 5 post-injection. Treatment with A16 significantly improved the memory function of APP/PS1 mice on day 9 post-injection compared with that of APP/PS1 control mice thereafter. After injecting with A16 again on day 10, the memory function of APP/PS1 mice was significantly impaired on day 11, but recovered markedly to a higher level on day 20 post 1st A16 treatment than that of APP/PS1 control mice (Fig. 5b). To further confirm these results, APP/PS1 mice were treated with A16, Fab16, or isotype control antibody by ICV administration twice biweekly, and then cognitive performance of these mice was evaluated at 2-week post 2nd antibody treatment. Expectedly, both A16 and Fab16 effectively rescued cognitive decline in APP/PS1 mice, as evaluated using Y-maze (Fig. 5c) and NOR tests (Fig. 5d). Consistently, significant increases in the number of synapses (Fig. 5e, f) and dendritic spine density (Fig. 5g, h) were observed in APP/PS1 mice treated with A16 and Fab16, relative to those of mice treated with vehicle or control IgG. These findings were confirmed by western blot analysis of PSD95 levels (Supplementary Fig. 28a, b). Similar levels of PSD95, Aβ, and C1q were detected in the microglia of APP/PS1 mice treated with A16 and Fab16, which were much lower than those of mice treated with vehicle or control IgG (Supplementary Fig. 28c–f and Supplementary Fig. 29).

### Treatment with full effector antibody attenuates neuropathology of APP/PS1 mice at 2-week post 2nd antibody treatment

Treatment with A16 and Fab16, but not with IgG isotype control, markedly reduced the plaque burden (Supplementary Fig. 30a, b), Aβ levels (Supplementary Fig. 30c–f), gliosis (Supplementary Fig. 30g–k), and cytokine levels (Supplementary Fig. 30l) in APP/PS1 mouse brains at 2-week post 2nd antibody treatment. The immunoreactivity of C1q (Supplementary Fig. 31a, b), transcripts of C1q, C3, and CR3 (Supplementary Fig. 31c), and C1q protein levels (Supplementary Fig. 31d, e) were also significantly decreased in the brains of A16- and Fab16-treated APP/PS1 mice. Moreover, the skeleton and Sholl analysis revealed that the ramified microglia significantly increased in the brains of APP/PS1 mice treated with A16 and Fab16, relative to those of vehicle- or control IgG-treated mice (Supplementary Fig. 31f–i). These findings suggest that the Aβ-targeting antibody ameliorated neuropathology in APP/PS1 mice at 2-week post 2nd antibody treatment.

### The rate of synaptogenesis in humans is much lower than that in mice

We firstly test whether synaptogenesis can be induced at a later timepoint in neuron-microglia cocultures after A16 treatment and AβO challenge. We found that PSD95 levels were much lower in A16-treated cocultures than those treated with Fab16 at 36 h after fresh medium replacement, while such synaptic density recovered and reached a level similar as that of the cocultures treated with Fab16 or just vehicle at 144 h (Supplementary Fig. 20c, d).

To compare the rates of synaptogenesis in humans and mice, we mimicked Aβ-mediated damage to synapses in AD brain by adding 500 nM AβOs to the neurons derived from neuronal stem cells of humans and mice, respectively, and then AβOs were removed after 24 h-incubation. The results showed that AβOs induced a significant decrease in PSD95 levels by 53% in mouse neurons and 42% in human neurons. After that, the PSD95 levels in mouse neuronal cultures recovered to approximately 91% at 96 h, while the levels of PSD95 in human neuronal cultures only reached 65% at 192 h, and 81% at 528 h (Supplementary Fig. 32). These findings suggest that the rate of synaptogenesis in humans was much lower than that in mice.

## Discussion

Microglia, which maintain CNS homeostasis by performing immune-related and phagocytic functions, continuously contact dendritic spines to regulate structural synaptic changes, and eliminate unneeded synapses during development and throughout life.^25,26^ Classical complement cascade may mediate this process by the binding of C1q and C3 to synapses and microglial CR3 activation.^25,27^ However, synaptic pruning by microglia significantly elevated in an AβO, C1q, and C3-dependent manner in patients with AD, leading to increased synapse loss.^8^ Aβ may upregulate C1q expression in microglia and astrocytes, resulting in more C1q binding to synapses, and consequent promotion of C3 activation and deposition on synapses. This cascade causes aberrant synapse elimination by microglia via CR3. Our present study showed that treatment with an Aβ-targeting antibody markedly exacerbated microglial synapse engulfment in vitro and in vivo, these events occurred via the activation of the complement system by antibody-Aβ complexes, observed as the colocalization of Aβ, antibody, C1q, C3, PSD95 and synaptophysin in microglia (Figs. 2, 4 and Supplementary Figs. 2, 20). Fab16, which lacks of Fc effector function, or using functional antibodies against CR3 and FcγRIIb, can significantly prevent antibody-induced microglial synapse phagocytosis.

Activated microglia contribute to neuronal damage in AD patients and transgenic mice by impairing Aβ clearance and overexpression of inflammatory cytokines (such as IL-1β, IL-6, and TNF-α), resulting in neurodegeneration in the corresponding brain regions.^28,29^ Our present study showed that microglia also released a large amount of proinflammatory factors while engulfing Aβ and synapses (Fig. 3l). Clinical trials of Aβ-targeting antibodies showed a high incidence of ARIA,^30^ such as vasogenic edema in patients, it is likely caused by the binding of antibodies to vascular Aβ, followed by the activation of complement and microglia via CR3 or FcγR, which leading to the generation of soluble complement components and proinflammatory cytokines. To attenuate these adverse events, IgG4 subtypes and several modified antibodies with reduced affinity for FcγR and C1q have been tested in clinical trials. Although they decreased the incidence of ADCC, CDC, vasogenic edema, and microhemorrhage, these antibodies still did not demonstrate adequate therapeutic efficacy.^13,14^ These findings indicate that merely reducing the risk of inappropriate proinflammatory response do not increase therapeutic efficacy, and other mechanisms may underlie clinical failures. In our present study, we showed that an IgG4 subtype Aβ-targeting antibody, A16I4, activated the complement system and increased microglial synapse elimination (Supplementary Figs. 24, 26, 27) although it significantly attenuated proinflammatory response.

For the four subclasses of IgG (IgG1-4) antibody, they display distinct complement activation profiles. IgG1 subclass antibody such as A16 is considered to be the most efficient activators for classical complement cascade via C1q binding,^31,32^ while IgG4 fails to bind to C1q and thus it is incapable of driving classical complement activation.^33^ However, IgG4 subclass antibodies are able to activate complement via the alternative or lectin pathways.^22,23^ It is reported that C3 amplifies complement cascade independent of the initiation route.^33^ In our study, although IgG1 subclass antibody A16 and IgG4 antibody A16I4 were capable of activating distinct complement pathways, they both activated C3, and led the binding to CR3, facilitating microglia-mediated phagocytosis of synapse and resultant synapse loss. Our findings suggest that synapse loss, rather than proinflammatory response and other side effects, may underlie clinical failures of AD immunotherapy.

Our results showed that full effector antibody A16 induced substantial microglia-mediated synapse loss in APP/PS1 mice, resulting in significant cognitive deficits, increased gliosis, and high levels of pro-inflammatory factors, this cascade occurred via activation of complement, CR3, and FcγR within 24-48 h post-injection. However, these detrimental effects of A16 gradually abated, significantly improved cognitive performance and reduced gliosis were observed in APP/PS1 mice at 2-week post 2nd A16 treatment. It is possible that after injection, A16 rapidly bound to the Aβ on the synapse, activated the classical complement cascade, and induced microglial engulfment of antigen-antibody complexes together with synapses, resulting in increased synapse loss. During the process of microglial synapse phagocytosis, Aβ was also engulfed and degraded, such Aβ clearance improved CNS microenvironment, gradually promoted synapse regeneration and recovery, and reversed cognitive deficits in antibody-treated APP/PS1 mice. These positive results, obtained during long-term post-treatment follow-up, were consistent with the observations in numerous preclinical studies.^11^ However, these processes would not readily happen in the brains of AD patients after immune treatment. It is reported that synaptogenesis in primates or humans reached a peak at early adolescence, and then rapidly declined with age,^34^ while the peak in rodents appeared at about 3 months of age and then the peak value did not decrease significantly. Moreover, the neurogenesis rate in 9-12 month-old mice (corresponding to about 40-year-old humans) was 10-20 times higher than that in humans.^35^ Our present studies also confirmed that the rate of synaptogenesis in humans was much lower than that in mice, which may be the cause that the engulfed synapses in the brains of AD patients were not rapidly replenished after antibody treatment in clinical trials. These findings may also explain the poor efficacy of immunotherapies and their failure in clinical trials for AD treatment.

We here also simulated the intracerebral environment with excessive Aβ-targeting antibody in neuron-microglia cocultures. When the cell cocultures were preincubated with A16 before Aβ challenge, A16 effectively prevented the binding of Aβ to synapses, and no microglial synapse engulfment was observed (Supplementary Fig. 23d, e). To gain excessive antibody levels in brains of patients with AD, a long-term treatment regimen with high doses of antibody is necessary to deplete the existing and newly-generated Aβ. However, antibody generally fails to remove all the Aβ from CNS under the conditions of clinical trials,^36,37^ and Aβ presents at excess levels relative to antibody, and can, therefore, bind to synapses and neurons before being targeted by antibody. The binding of therapeutic antibodies to the pre-absorbed Aβ on synapses triggers activation of the complement cascades, leading to synapse elimination by microglia.

Based on the present evidences, we propose that any causative-agent targeting immunotherapy for brain disorders, whether it uses active or passive strategies, or whether the causative agent is Aβ, tau, α-synuclein, or other factors, can result in neuronal damage, as long as the antibody with effector function and causative agents located on neuronal surface. Both our present and previous results showed that an Aβ-targeting antibody without effector function effectively reduced Aβ levels, attenuated cognitive deficits and neuropathology without neuronal damage.^38^ These results suggest that antibody lack of effector function may achieve the expected clinical efficacy, although the underlying mechanism by which it reduces Aβ levels remains elusive.

In summary, our study explored the causes for different outcomes between the preclinical and clinical studies of anti-AD immunotherapies. Treatment with full effector antibody against Aβ induced robust microglial phagocytosis of synapses via complement activation. Antibody lack of effector function, or with reduced effector function, can overcome such critical defects and simultaneously exert beneficial effects, thereby showing promise in the treatment of patients with AD.

## Materials and methods

### Antibodies

The following primary antibodies were used in immunocytochemistry and immunohistochemistry studies: anti-C1q (Invitrogen, MA1-40311, lot TD2550204, 1:50); anti-C3 (Abcam, ab200999, lot GR294205-17, 1:200; Hycult, HM1065, lot 23152M1017-A, 1:50); anti-Iba-1 (Abcam, ab178847, lot GR3229566-2, 1:100; GeneTex, GTX101495, lot 41885, 1:50); anti-GFAP (Cell Signaling Technology, 3670S, lot 5, 1:100); anti-LAMP1 (Abcam, ab24170, lot GR3235359-1, 1:100); anti-MAP2 (Abcam, ab11267, lot GR281093-9, 1:100); anti-PSD95 (Abcam, ab12093, lot GR3271883-1; ab18258, lot GR3174013-1, 1:100); anti-Synaptophysin (Abcam, ab32127, lot GR312544-1, 1:100); 4G8 (Biolegend, 800704, lot B238676, 1:100); anti-β-actin (MBL, M177-3, lot 002, 1:1000); mouse anti-rat CD11b (BD Biosciences, 554980, lot 6294728); mouse IgA, κ isotype control (BD Biosciences, 553476, lot 7257918); mouse anti-rat CD32 (BD Biosciences, 550273, lot 8339705); mouse IgG1κ isotype control (BD Biosciences, 553447, lot 8241620).

### Generation of antibodies of A16, Fab16 and A16I4

The Aβ-targeting antibody A16 was generated by immunizing Balb/c mice with human Aβ 1-16 (Chinese Peptide Company, Hangzhou, China) conjugated to keyhole limpet hemocyanin (KLH) using standard mouse immunization and hybridoma screening technologies. Fab16 was prepared by digestion A16 with ficin using Pierce™ Mouse IgG1 Fab and F(ab’)_2_ Preparation Kit (Thermo, 44980) according to the manufacturer’s instructions. A16 was further humanized onto an IgG4 backbone to generate A16I4. The endotoxin level was less than 0.25 EU/mg as measured by the limulus amebocyte lysate (LAL) assay.

### Microscale thermophoresis assay

The affinity of the antibodies with Aβ were calculated by microscale thermophoresis (MST) assay using Monolith NT.115 (NanoTemper Technologies, Germany). The MST power and LED excitation power were set at 40%. Experiments were performed in PBS containing 0.05% tween 20 with standard capillaries. The concentration of FAM-Aβ (AMYD-005A; Chinese Peptide Company, Hangzhou, China) was 40 nM, while A16, Fab16, A16I4 or 6E10 were 2-fold serial diluted (14 points per curve). The binding affinity was calculated using the Kd model in the MO. Affinity Analysis software v2.3. All experiments were performed at least three times.

### Preparation of Aβ42 oligomers

Aβ42 (AMYD-003A; Chinese Peptide Company, Hangzhou, China) was dissolved in PBS at 100 μM and incubated at 37 °C without agitation for 4-8 h. Oligomers were then separated by size exclusion chromatography. The molecular weight of the oligomers in use is about 146 kDa. The endotoxin level in PBS was less than 0.03 EU/ml.

### Thioflavin T fluorescence assay

For Aβ aggregation, 20 μM Aβ was incubated at 37 °C without agitation in the presence or absence of 2 μM antibodies. The aggregation kinetics of Aβ was monitored by Thioflavin T (ThT) fluorescence intensity using the method described previously.^39^ Data were obtained from three independent experiments.

### Primary neurons and microglia

Female Sprague Dawley rats were purchased from Vital River Laboratories (Beijing, China). Primary neurons were obtained from hippocampi of rat embryos on embryonic day 17 (E17) or E18, seeded on poly-D-lysine coated coverslips at a density of 300,000/well in 12-well dish, and cultured in neurobasal medium with B27 and L-GlutaMAX. Primary microglia were prepared from hippocampi and cortices of postnatal (P1–P2) pups, and cultured in DMEM with 10% FBS and 1% penicillin/streptomycin in 75 cm^2^ flasks for 12 days. Thereafter, microglia were shaken off by using a rocking platform shaking for 60 min at 250 r.p.m. For neuron-microglia cocultures, microglia were pelleted and resuspended in neurobasal medium and added to neurons (DIV 7–10) as 1:3 ratio (microglia: neuron).

For microglial engulfment analysis, 500 nM AβOs and/or 250 nM antibodies were added to neuron-microglia cocultures and the cocultures were further incubated for 10 h. Cells were washed three times with PBS before fixation. Immuno-stained cultures were imaged on a laser scanning confocal microscope (Leica TCS SP8, Germany).

### MTT assay

Quantification of cell viability via MTT assay was performed as previously described with some modifications.^40^ Briefly, N2a cells (obtained from the cell line resource center of Peking Union Medical College, Chinese Academy of Medical Sciences) were maintained in DMEM medium containing 10% FBS and seeded in 96-well plates with approximately 5000 cells per 100 μL of medium per well. Cells were treated with 4 µM AβOs in the presence or absence of 2 µM antibodies and then incubated for an additional 72 h at 37 °C. Cell viability was measured by MTT assay. Data were obtained from three independent experiments.

To detect the cytotoxicity of 500 nM AβOs, primary neurons were seeded in 96-well plates with approximately 10000 cells per 100 μL of medium per well. Plates were incubated at 37 °C for 1 week to allow cells to grow. Cells were treated with 500 nM AβOs and then incubated for an additional 12 h at 37 °C. Cell viability was determined by MTT assay.

### Neuronal differentiation from human neuronal stem cells

Human neuronal stem cells (NSCs, purchased from Wuhan Sunncell Biotechnology Co., Ltd) were cultured in Matrigel-coated 12-well plates (50,000 cells per well) for 3 days. Then cells were incubated in DMEM/F12 medium for neuronal differentiation, which containing 1% N2 supplement, 1% B27 supplement, 200 μM ascorbic acid, 400 μM dbcAMP, 10 ng/ml GDNF, and 10 ng/ml BDNF. After 2 days incubation, Laminin was supplied to the cell cultures to facilitate differentiation. Cells were cultured for 14 days and the medium was changed every day.^41^

### Neuronal differentiation from mouse neuronal stem cells

Neural stem cells were obtained from 13 to 16 days of fetal mouse brains and cultured to form neurospheres in growth medium with 2% N2 supplement, 2% B27 supplement, 20 ng/ml bFGF, and 20 ng/ml EGF. The neurospheres were then digested with accutase for 10 min at 37 °C and seeded in PDL-coated 12-well plates (50,000 cells per well). Cells were then cultured for 8 days in differentiation medium containing 1% N2 supplement, 1% B27 supplement, and 0.5% FBS.^42^

To measure the rate of synaptogenesis between human and mouse, 500 nM AβOs were added to neurons derived from the neural stem cells of human and mouse, respectively. After 24 h incubation, the medium was replaced to fresh medium and the neuronal cultures were further incubated for the indicated time. PSD95 level in cultured neurons were measured by western blots every 24 h.

### Immunocytochemistry

Immunocytochemistry study was carried out as described previously.^43^ Briefly, cells were processed for immunofluorescence by incubating with primary antibodies for 1 h at room temperature, followed by corresponding Alexa-conjugated (-488, -594, or -647) secondary antibodies, respectively, and then counterstained with Hoechst (Cat# C0021, Solarbio, 1:100). Fluorescence signals were captured on a laser scanning confocal microscope (Leica TCS SP8, Germany).

For dendritic spines staining, anti-MAP2 antibody was used followed by Alexa 488-conjugated secondary antibody and Alexa Fluor 555 phalloidin (Abcam, ab176756). The images were acquired by Leica TCS SP8 confocal microscope.

### CR3 and FcγRIIb functional blocking in neuron-microglia cocultures

For CR3 blockade, neurons were pretreated with AβOs (500 nM) for 30 min followed by A16 (250 nM) for another 30 min, while microglia were pretreated with either 15 μg/ml of anti-CD11b functional antibody or 15 μg/ml of isotype control for 30 min. Then the microglia were added to neurons and the cocultures were further incubated for 10 h. Cells were processed for immunofluorescence using anti-PSD95, anti-Iba-1, and 4G8 antibodies, respectively. Images were captured using Leica TCS SP8 confocal microscope. For FcγRIIb blockade, 10 μg/ml of anti-CD32 functional antibody or 10 μg/ml of isotype control were used to treat microglia and the experiments were carried out in the same way.

### Immunoprecipitation

A16 or IgG1 control antibody were cross-linked to protein A magnetic beads (#1614833, Bio-Rad). Then cell lysates from N2a-695 were incubated with such A16 or IgG1 beads overnight at 4 °C. After washing step, proteins were eluted from the magnetic beads using 20 mM glycine (pH 2.0) for three to five rounds. The resulting protein solution was neutralized with 1 M tris buffer (pH 10.0), and the proteins were analyzed by western-blotting.

### Mice and treatment

APP/PS1 mice were originally obtained from Jackson Laboratory (line 85, Stock No: 004462), which expressing a chimeric mouse/human amyloid precursor protein (Mo/HuAPP695swe) and a mutant human presenilin 1 (PS1-dE9), both directed to CNS neurons. All mice for experiments were provided food and water *ad libitum*, group-housed in a colony room at 22 ± 2°C and 45% ±10% humidity on a reverse 12 h light/dark cycle. All experiments were performed in accordance with the China Public Health Service Guide for the Care and Use of Laboratory Animals. Experiments involving mice and protocols were approved by the Institutional Animal Care and Use Committee of Institute of Process Engineering, Chinese Academy of Sciences. Male APP/PS1 mice at 3, 5, 6 or 10 months of age were ICV injected with A16 (AD-A16), Fab16 (AD-Fab16), 6E10 (AD-6E10), isotype control antibodies (AD-Iso) or vehicle (AD-Veh), respectively. WT littermates were given vehicle (WT-Veh). For short-term study, behavioral studies were performed at 24 h post injection. For repeated antibody treatment study, mice were received additional ICV injections of antibodies or vehicle 14 days following the initial ICV injections, and the behavioral studies were performed at 2-week post 2nd antibody treatment. All mice were then sacrificed and the neuropathology in brains were analyzed.

To track changes in the cognitive performance of 6-month-old APP/PS1 mice treated with A16, APP/PS1 mice were ICV injected with A16, and the cognitive function of mice was tested by Y-maze at 0, 1, 3, 5, 7, and 9 days post-treatment. After that, APP/PS1 mice were received additional ICV injections of A16 on Day 10, and the cognitive performance was evaluated by Y-maze at 11 and 20 days post-1st A16 treatment. In this study, we had 16 groups of APP/PS1 mice in parallel (8 groups received A16 treatment, and 8 groups received vehicle only), each group were applied for Y-maze test just once at certain timepoint to avoid behavior confounds.

For A16I4 study, APP/PS1 mice were intravenously injected with 20 mg/kg of A16I4 (AD-A16I4), A16 (AD-A16) or vehicle (AD-Veh) via the tail vein, respectively. WT littermates were given vehicle (WT-Veh). All mice were sacrificed 48 h post injection and microglia engulfment analysis were performed.

For brain harvesting, mice were deeply anaesthetized and transcardially perfused with ice-old PBS containing heparin (10 U/mL) before sacrificed. The left brain hemisphere was fixed in 4% PFA at 4 °C overnight and then paraffin embedded. Serial coronal sections were cut at 6 μm thickness on a Lecia CM1850 microtome (Leica Biosystems, Buffalo Grove, IL, USA).

### Stereotaxic antibody injection

Mice were anesthetized with 3% isoflurane and performed surgery on a stereotaxic apparatus. The stereotactic injection coordinate was +0.5 mm anteroposterior, 1 mm mediolateral and −2.5 mm dorsoventral. 5 μL of A16, Fab16, isotype control (all as 24 μM) or PBS were injected at a rate of 0.5 μL/min. The needle was removed 5 min after injection completed. Mice were put back to their cage until behavioral study.

### Behavioral phenotyping

Y-maze test was conducted as previously described in detail.^44^ NOR test was performed as described previously with some modifications.^44,45^ Briefly, mice were individually habituated to explore the behavioral arena for 5 min 24 h before testing, and then were allowed to explore for 5 min in the training session. After a 6 h retention period, mice were reintroduced to the box and allowed to explore for 5 min in the testing session. The discrimination index of each mouse was calculated by subtracting the time spent exploring the familiar object from the time spent exploring the novel object and dividing this by the total exploration time.

### Immunohistochemistry

Immunohistochemistry analysis was conducted as previously described.^43^ For immunofluorescence staining, the following primary antibodies were used: anti-PSD95, anti-synaptophysin or anti-C1q antibodies, followed by corresponding secondary antibodies conjugated to Alexa Fluor 488 or 594, respectively. Images were captured by Leica TCS SP8 confocal microscope. For 3′-Diaminobenzidine (DAB) immunostaining, the following primary antibodies were used: 4G8, anti-Iba-1 or anti-GFAP antibodies, followed by corresponding HRP-labeled secondary antibodies and visualized with DAB by Olympus IX73 inverted microscope with a DP80 camera.

For quantification of label, three to seven sections spanning the cortex or hippocampus were analyzed for each mouse. Six images were acquired on matching areas of per section. Values from each section of per mouse were averaged. All images were analyzed by ImageJ Software (National Institutes of Health, USA). The experimenters were blinded to the treatment groups.

For microglial engulfment assays, mouse brains were fixed in 4% PFA overnight, and cryoprotected in 30% sucrose. 40-μm thickness coronal frozen sections were processed for immunostaining by incubating with 4G8, anti-Iba-1, anti-PSD95, anti-synaptophysin, anti-C1q, or anti-C3 antibodies overnight at 4 °C, followed by corresponding secondary antibodies conjugated to Alexa Fluor 488, 546, 647 or Streptavidin-Cy3 (Sigma, S6402, 1:200). Fluorescence signals were captured on Leica TCS SP8 confocal microscope. All images were analyzed by ImageJ Software (National Institutes of Health, USA). Three-dimensional volume surface renderings for microglia and engulf inputs was created by Imaris software (Bitplane). Total volume of engulfed inputs in microglia was quantified. 6–9 microglia were analyzed per mouse. Experiments and data analyses were performed blind.

For the study of microglial morphology, Sholl analyses and skeleton analyses of individual microglia were performed using ImageJ Software.^46,47^ For Sholl analyses, Iba-1^+^ microglia were cropped and serial equidistant radiating concentric circles (5 μm) were plotted from the center of microglia cell body to the furthest radiating extent of ramification. Skeleton analyses calculated the maximum branch lengths and number of branches of microglial cells. All analyses were performed blind to treatment group.

### Golgi staining

For Golgi staining, fresh brain hemispheres were processed with FD Rapid GolgiStain^TM^ kit (Cat# PK401, FD NeuroTechnologies, Columbia, MD) according to the manufacturer’s instructions. Serial coronal sections of 100 μm thickness were cut on a Lecia CM1850 microtome (Leica Biosystems, Buffalo Grove, IL). Images of Golgi-impregnated dendrites from cortex neurons were captured with Olympus IX73 microscope with 100× oil immersion lens. Spine density was quantified by manual counting in ImageJ and normalized to the length on the dendritic segment. 10–12 segments were analyzed per mouse.

### RNA extraction and quantitative PCR (q-PCR)

Total RNA from brain and cell lysates was isolated using the RNeasy Lipid Tissue kit (Cat# 74804, QIAGEN, Valencia, CA). cDNA was prepared from 1.5 μg of RNA by using a PrimeScript RT-PCR kit (Cat# RR037Q, Takara, Beijing, China). Q-PCR were performed with 7500 Fast Real-Time PCR System (Applied Biosystems) using SYBR Select Master Mix (Cat# 4472908, Applied Biosystems) for gene amplification and detection. Target gene expression levels were normalized to β-actin or GAPDH. The following primers were used: 5′-GGAGGCAGGAACATCATGGAGA-3′ and 5′- AATTCCTGCAACCCCGTCCT-3′ for C1qa (rat); 5′-TCTCAGCCATTCGGCAGAAC-3′ and 5′-TAACACCTGGAAGAGCCCCTT-3′ for C1qa (mouse); 5′- CGATGATCCTTGACATCTGCACC-3′ and 5′- GTGGTGGTCAGTTGGGGCAGCCG-3′ for C3 (rat); 5′-AACTGCTGGCCTCTGGAGTA-3′ and 5′-GCATGATTCCTCGAGGTTGT-3′ for C3 (mouse); 5′-CATCACCGTGAGTTCCACAC-3′ and 5′-GAGAACTGGTTCTGGCTTGC-3′ for CD11b (rat); 5′- CCAAGACGATCTCAGCATCA-3′ and 5′-GGATGATCCCATACGGTCACC-3′ for CR3 (mouse); 5′-GGAACCCTGCTGTTCCTACC-3′ and 5′-CAGCCTTCGGAAGACCATGA-3′ for FcγRIIb (rat); 5′-TGACAGGATGCAGAAGGAGA-3′ and 5′-GTACTTGCGCTCAGGAGGAG-3′ for β-actin (mouse); 5′-TGAAGGTCGGTGTCAACGGATTTGGC-3′ and 5′-CATGTAGGCCATGAGGTCCACCAC-3′ for GAPDH (rat); 5′-GATTATGGCTCAGGGTCCAA-3′ and 5′-GCTCCAGTGAATTCGGAAAG-3′ for TNF-α (mouse); 5′-CCCAAGCAATACCCAAAGAA-3′ and 5′-GCTTGTGCTCTGCTTGTGAG-3′ for IL-1β (mouse); 5′-CCGGAGAGGAGACTTCACAG-3′ and 5′-TTGCCATTGCACAACTCTTT-3′ for IL-6 (mouse); 5′-CACCTGGAACAGCACTCTCT-3′ and 5′-CTTTGTGCGAAGTGTCAGTG-3′ for iNOS (mouse); 5′-TTCAGGGTTTGGGTTAGTGA-3′ and 5′-GGGCCTTTTATGGATGTCTT-3′ for Clec7a (mouse); 5′-CCTCTTGCTGCCTCTCATCATTGG -3′ and 5′-GGCTGGTAGGTTGATTGTCGTCTG -3′ for CD68 (mouse).

### Brain lysate preparation

The right half brain hemisphere of mice was homogenized in TBS buffer containing protease inhibitor cocktail (Cat# 539131-1VL, Sigma) and centrifuged at 14000 × *g* for 30 min at 4 °C, to obtain the supernatant (TBS-soluble fraction) containing soluble Aβ. The pellets were then resuspended in guanidine buffer (5.0 M guanidine-HCl/50 mM Tris-HCl, pH 8.0) and centrifuged at 14,000 × *g* for 1 h at 4 °C, the supernatants (guanidine-soluble fraction) containing insoluble Aβ were obtained.

### Aβ measurement

The levels of Aβ40 and Aβ42 were determined by electrochemiluminescence (ECL) assay using 96-well MULTI-ARRAY Multiplex Kits (Cat# K15199E-2, Meso Scale Diagnostics, Gaithersburg, MD). The TBS- and guanidine-soluble fractions of brain lysates were processed according to the manufacturer’s instructions. Aβ levels were measured using a SECTOR S 600 reader (Meso Scale Diagnostics).

### Western blot

Protein samples were separated by 12% SDS-PAGE gel (Invitrogen) and transferred to nitrocellulose membranes (Merck Millipore). The membrane was blocked with 5% nonfat milk, and then probed with anti-Iba-1 (1:1000), anti-GFAP (1:1000), anti-PSD95 (1:1000), anti-synaptophysin (1:1000), anti-C1q (1:1000) and anti-β-actin (1:1000) antibodies for 1 h at room temperature, respectively, followed by IRDye secondary antibodies (Li-Cor, Rockland). Immunoreactivity was detected using Odyssey Imaging System (Li-Cor) and analyzed using Image Studio Software (Li-Cor).

### Measurements for inflammatory cytokines

The levels of IL-1β, IL-6, and TNF-α in the brain lysates of mice were determined using corresponding ELISA kits (Biolegend, Cat# 432601 for IL-1β; Cat# 431304 for IL-6; Cat# 430904 for TNF-α) according to the manufacturer’s protocols. A SpectraMax M5 microplate reader (Molecular Devices, Sunnyvale, CA) was used to measure the absorbance at 450 nm.

### shRNA-expressing constructs and lentivirus infection

shRNAs were chemically synthesized from Invitrogen and inserted into the pSicoR-GFP lentiviral vector (Addgene, 12093). Lentivirus were prepared by transfecting the core and packaging plasmids into 293 cells using GenEscort I (Nanjing Wisegen Biotechnology), and collected at 48 h post-transfection.^48^ For lentivirus infection, lentivirus was added to primary microglia cultures for 8 h incubation. 2 μg/ml polybrene was added to the medium to facilitate infection. The following shRNAs were used: C1qa, GCACTGTGCTTCAATTGCAAC; C3, GCATGCGTGATATCCCTATGA; CD11b, GCACCTCGATATCAGCATATC; FcγRIIb, GGACCCACAACACCAAGAACT.

### Classical pathway hemolytic assay

A16 or Fab16 with Aβ was preincubated in 5% guinea pig serum for 40 min at 37 °C, and then the mixture was added to the amboceptor-coated sheep erythrocytes and incubated for additional 10 min at 37 °C. Lysis of erythrocytes was determined by measuring OD value at 541 nm. The results were calculated as hemolysis rate normalized to that of the control in absence of antibody, which was set as 100%.

### Statistical analysis

Significant differences in data were analyzed by GraphPad Prism v.8. using student’s *t*-test or one-way ANOVA followed by Tukey’s post hoc test, as appropriate. Results were expressed as group mean ± s.e.m. *P < 0.05, **P < 0.01, ***P < 0.001 and ****P < 0.0001 were considered statistically significant.

## Supplementary information

Supplementary_Materials.doc

Supplementary Movie 1

Supplementary Movie 2

## Data Availability

The data in this study are available from the corresponding author upon reasonable request.
