# Supplementary material for: Fc effector of anti-Aβ antibody induces synapse loss and cognitive deficits in Alzheimer’s disease-like mouse model
Source: Signal Transduct Target Ther. 2023 Jan 25;8:30. doi: 10.1038/s41392-022-01273-8 (PMC9873795; doi:10.1038/s41392-022-01273-8)
Supplement: Supplementary file 1 — Supplementary_Materials.doc [file 41392_2022_1273_MOESM1_ESM.docx]

Supplementary Materials for

Fc effector of anti-Aβ antibody induces synapse loss and cognitive deficits in Alzheimer’s disease-like mouse model

Xiao-ying Sun^1,3,†^, Xiao-lin Yu^1,2,†*^, Jie Zhu^1,3,†^, Ling-jie Li^1,3^, Lun Zhang^1,2^, Ya-ru Huang^1,3^, Dong-qun Liu^1^, Mei Ji^1^, Xun Sun^1^, Ling-xiao Zhang^1^, Wei-wei Zhou^1,2^, Dongming Zhang^4^, Jianwei Jiao^4^, Rui-tian Liu^1,2*^

^1^ *State Key Laboratory of Biochemical Engineering, Institute of Process Engineering, Chinese Academy of Sciences, Beijing 100190, China*

^2^ *Innovation Academy for Green Manufacture, Chinese Academy of Sciences, Beijing 100190, China*

^3^ *School of Chemistry and Chemical Engineering, University of Chinese Academy of Science, Beijing 100049, China*

^4^ *State Key Laboratory of Stem Cell and Reproductive Biology, Institute of Zoology,*

*Chinese Academy of Sciences, Beijing 100101, China*

*Corresponding author

†These authors contributed equally: Xiao-ying Sun, Xiao-lin Yu, Jie Zhu

Correspondence to: rtliu@ipe.ac.cn; yuxiaolin@ipe.ac.cn

**This PDF file includes:**

Supplementary Figures. S1 to S32

Captions for Movies. S1 to S2

**Other Supplementary Materials for this manuscript include the following:**

Movies S1 to S2


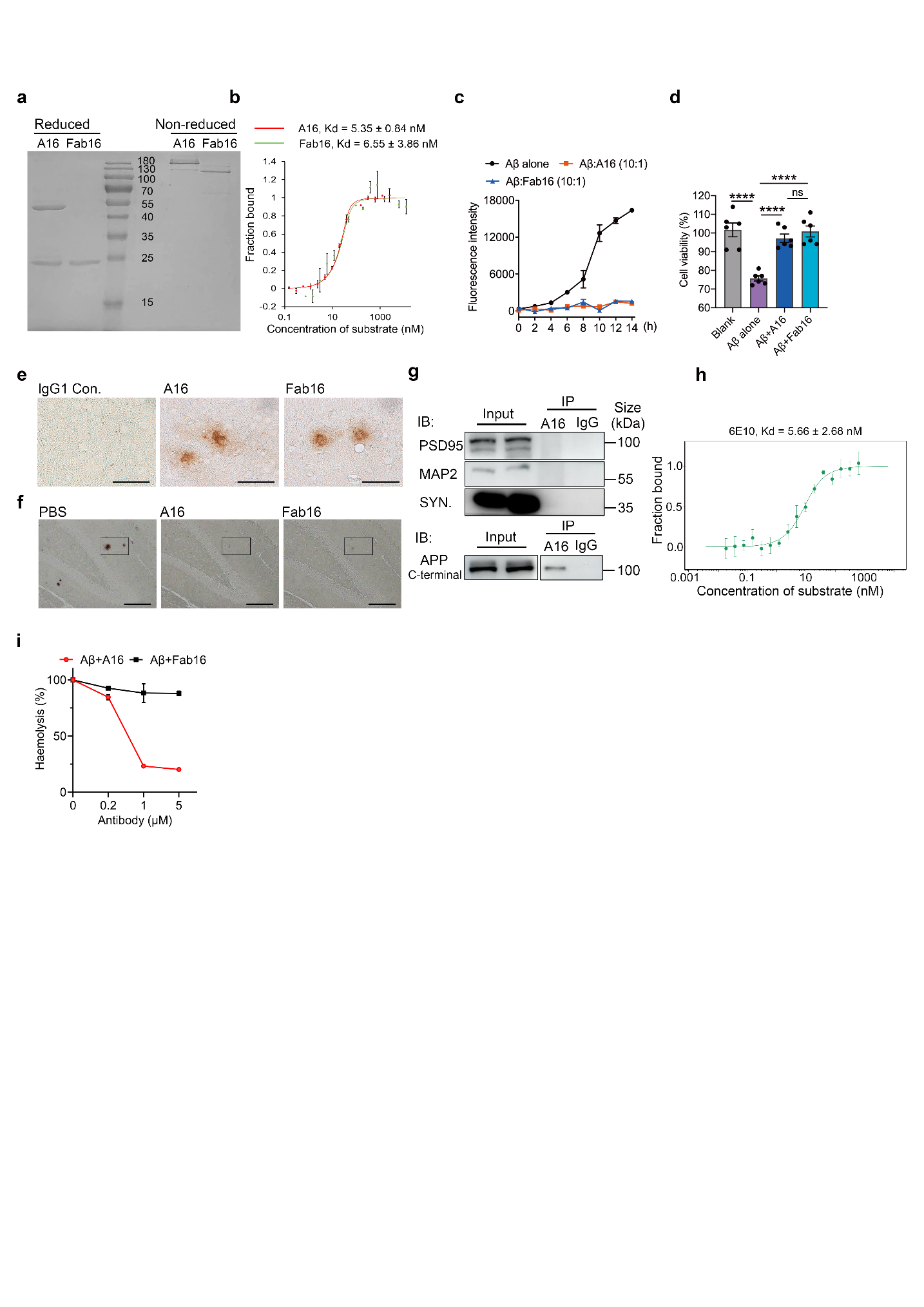


**Supplementary Fig. 1. The characterization of Aβ-targeting antibody A16 and effector-less antibody Fab16. a** A16 and Fab16 antibodies were separated by SDS-PAGE under reduced or non-reduced conditions. **b** The binding affinity of A16 and Fab16 for Aβ was measured using microscale thermophoresis (*n* = 3 independent experiments). **c** Aggregation kinetics of Aβ incubated with A16 or Fab16 were assessed by thioflavin T fluorescence (*n* = 3 independent experiments). **d** The viability of N2a cells treated with A16 or Fab16 in the presence or absence of AβOs for 72 h was determined using MTT assay (*n* = 6 biological replicates over three independent experiments). **e** A16 and Fab16 bind Aβ plaques present in brain sections from APP/PS1 mice. APP/PS1 brain sections were immunolabeled with A16, Fab16, or isotype IgG1 antibodies, followed by HRP-labeled anti-mouse IgG and visualized with DAB. Scale bar, 50 μm. **f** A16 and Fab16 disassemble Aβ plaques present in brain sections from APP/PS1 mice. APP/PS1 brain sections were incubated with A16 or Fab16 antibodies overnight and then immunolabeled with 4G8 antibody. Rectangles indicate plaque area. Scale bar, 200 μm. **g** Immunoprecipitation analysis of PSD95, MAP2, synaptophysin and APP-C terminal from N2a-695 cell lysates with A16 or IgG control antibodies. **h** The affinity of 6E10 for Aβ was measured using microscale thermophoresis (*n* = 3 independent experiments). **i** The immune-complex of A16-Aβ, but not that of Fab16-Aβ, activated the classical complement cascade when incubated with guinea-pig serum. The lysis of sheep erythrocytes by the terminal complement complex was assessed by measuring released hemoglobin at 541 nm (*n* = 3 independent experiments). Data are expressed as mean ± s.e.m. and analyzed by one-way ANOVA with Tukey’s test (**d**). *****P* < 0.0001; ns, not significant.


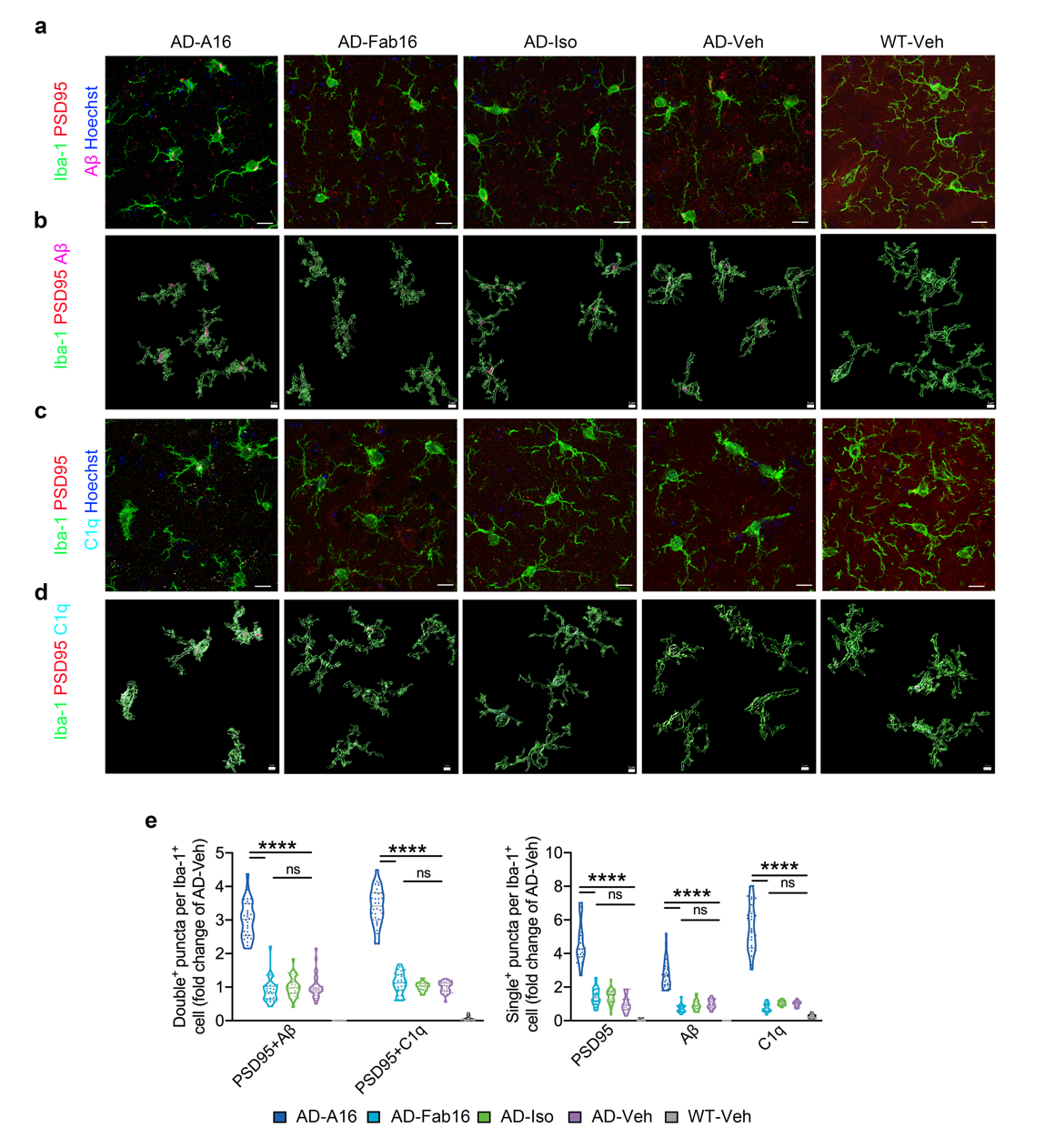


**Supplementary Fig. 2. Full effector antibody A16 significantly promotes microglial engulfment of synapses in 6-month-old APP/PS1 mice. a** Representative images of the engulfed PSD95 (red) and Aβ (magenta) puncta within Iba-1^+^ (green) microglial cells in the brains of APP/PS1 or WT mice treated with A16, Fab16, isotype control antibody, or PBS, at 48 h post-injection. Scale bar, 10 μm. **b** 3D surface rendering of Iba-1^+^ microglia (green) containing engulfed PSD95 (red) and Aβ (magenta) puncta in **a**. Scale bar, 5 μm. **c** Representative images of the engulfed PSD95 (red) and C1q (cyan) puncta within Iba-1^+^ (green) microglial cells in the brains of APP/PS1 or WT mice treated with A16, Fab16, isotype control antibody, or PBS, at 48 h post-injection. Scale bar, 10 μm. **d** 3D surface rendering of Iba-1^+^ microglia (green) containing engulfed PSD95 (red) and C1q (cyan) puncta in **c**. Scale bar, 5 μm. **e** Quantification of colocalized PSD95 and Aβ puncta, PSD95 and C1q puncta, and PSD95, Aβ and C1q puncta, per Iba-1^+^ microglial cell (*n* = 30 microglial cells from 5 mice). Data are displayed as violin plots and were analyzed by one-way ANOVA with Tukey’s test. Dotted lines represent medians and interquartile ranges. *****P* < 0.0001, ns, not significant.


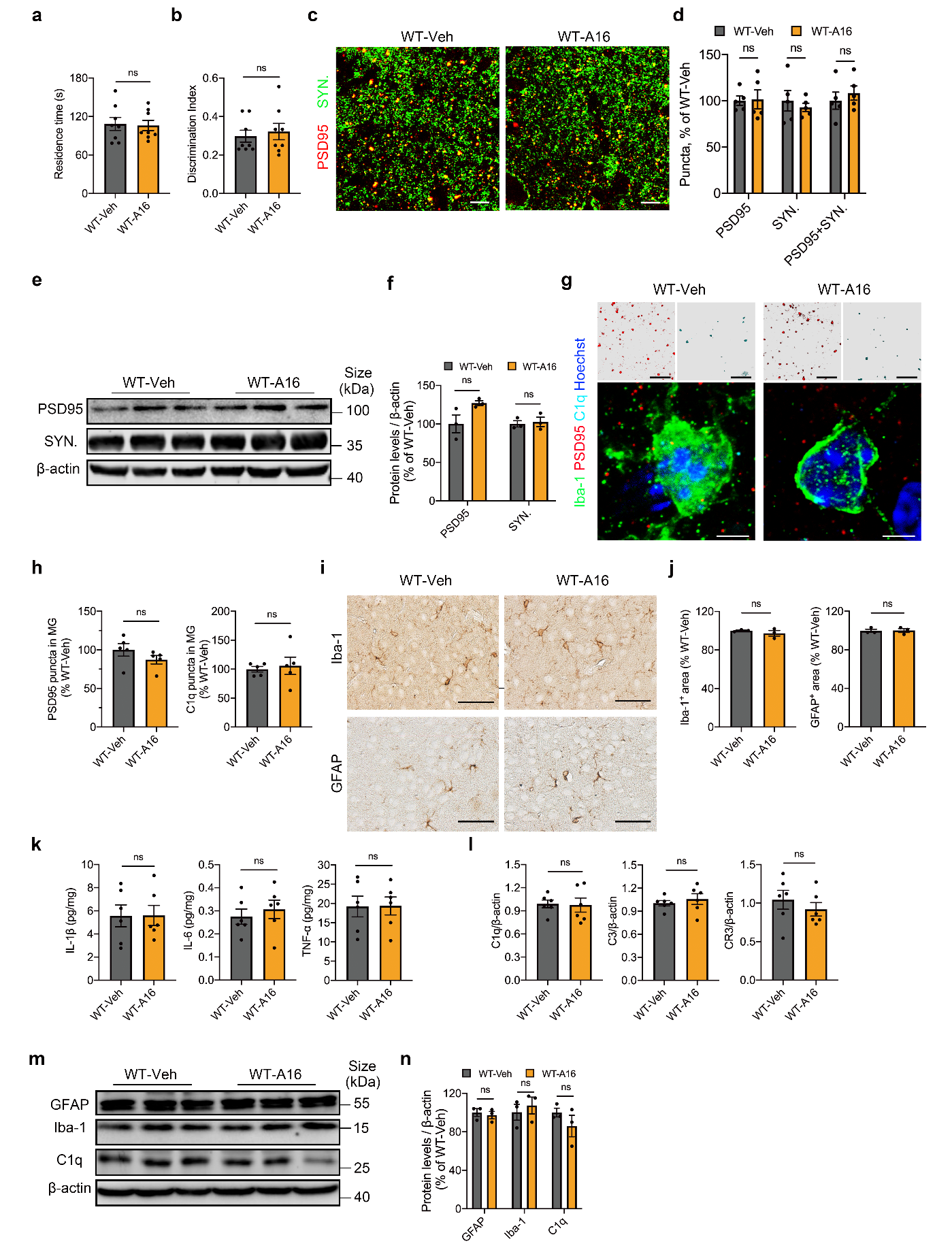


**Supplementary Fig. 3. Full effector antibody A16 fails to induce synapse loss and neuroinflammation in 6-month-old WT mice. a** The time spent in the novel arm of Y-maze by WT mice at 24 h post-treatment with A16 or PBS (*n* = 8 mice). **b** Discrimination index of the mice in novel object recognition test (*n* = 8 mice). **c** Immunolabeling of PSD95 (red) and synaptophysin (green) in the brains of WT mice treated with A16 or PBS at 48 h post-injection. Scale bar, 2.5 μm. **d** Quantification of synaptic puncta or their apposition in **c** (*n* = 5 mice). **e** Western blot analysis of PSD95 and synaptophysin in the brains of WT mice treated with A16 or PBS at 48 h post-injection. **f** Quantitation of PSD95 and synaptophysin expression in **e** (*n* = 3 mice). **g** Representative images of the engulfed PSD95 (red) and C1q (cyan) puncta within Iba-1^+^ (green) microglial cells in the brains of WT mice treated with A16 or PBS, at 48 h post-injection. Scale bar, 3 μm. **h** Quantification of PSD95 and C1q puncta per Iba-1^+^ microglial cell (*n* =5 mice). **i** Immunolabeling of Iba-1 and GFAP in the brains of WT mice treated with A16 or PBS, at 48 h post-injection. Scale bar, 50 μm. **j** Quantification of Iba-1- and GFAP-labeled area in **i** (*n* = 3 mice). **k** The levels of IL-1β, IL-6, and TNF-α in brain lysates of WT mice treated with A16 or PBS at 48 h post-injection (*n* = 6 mice). **l** qPCR analysis of *C1q, C3,* and *CR3* mRNA expression in the brains of WT mice treated with A16 or PBS, at 48 h post-injection (*n* = 6 mice). **m** Western blot analysis of GFAP, Iba-1, and C1q expression in the brains of WT mice treated with A16 or PBS, at 48 h post-injection. **n** Quantitation of GFAP, Iba-1, and C1q expression in **m**. *n* = 3 mice. Data are expressed as mean ± s.e.m. and were analyzed by Student’s *t*-test. ns, not significant.


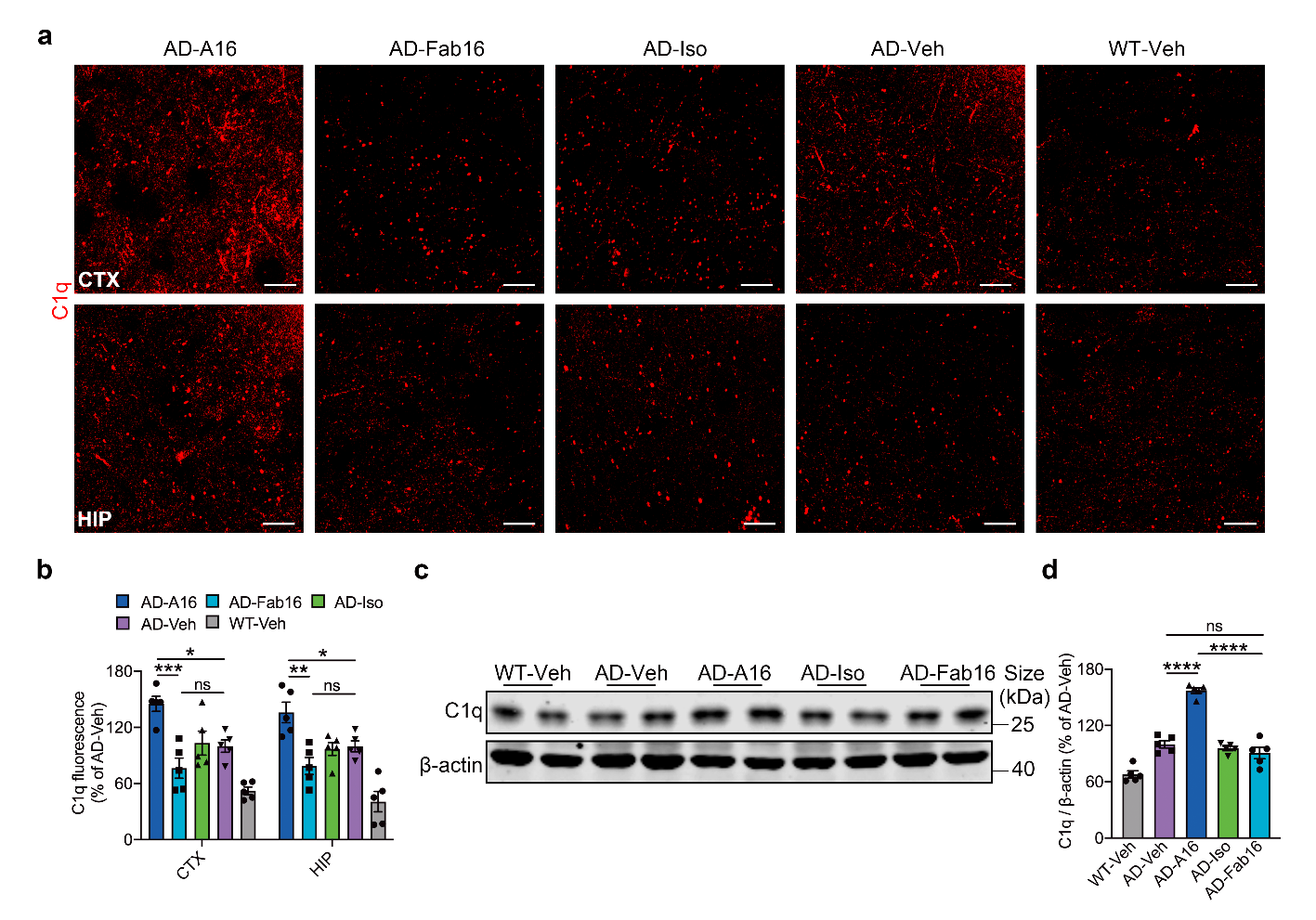


**Supplementary Fig. 4. Full effector antibody A16 activates classical complement cascade in the brains of 6-month-old APP/PS1 mice. a** Immunolabeling for C1q in the cortex and hippocampus of APP/PS1 or WT mice treated with A16, Fab16, isotype control antibody, or PBS, at 48 h post-injection. Scale bar, 10 μm. **b** Quantification of C1q immunofluorescence intensity in **a** (*n* = 5 mice). **c** Western blot analysis of C1q expression in the brains of APP/PS1 or WT mice treated with A16, Fab16, isotype control antibody, or PBS, at 48 h post-injection. **d** Quantitation of C1q expression in **c** (*n* = 5 mice). Data are expressed as mean ± s.e.m. and were analyzed by one-way ANOVA with Tukey’s test. **P* < 0.05, ***P* < 0.01, ****P* < 0.001, *****P* < 0.0001; ns, not significant.

**
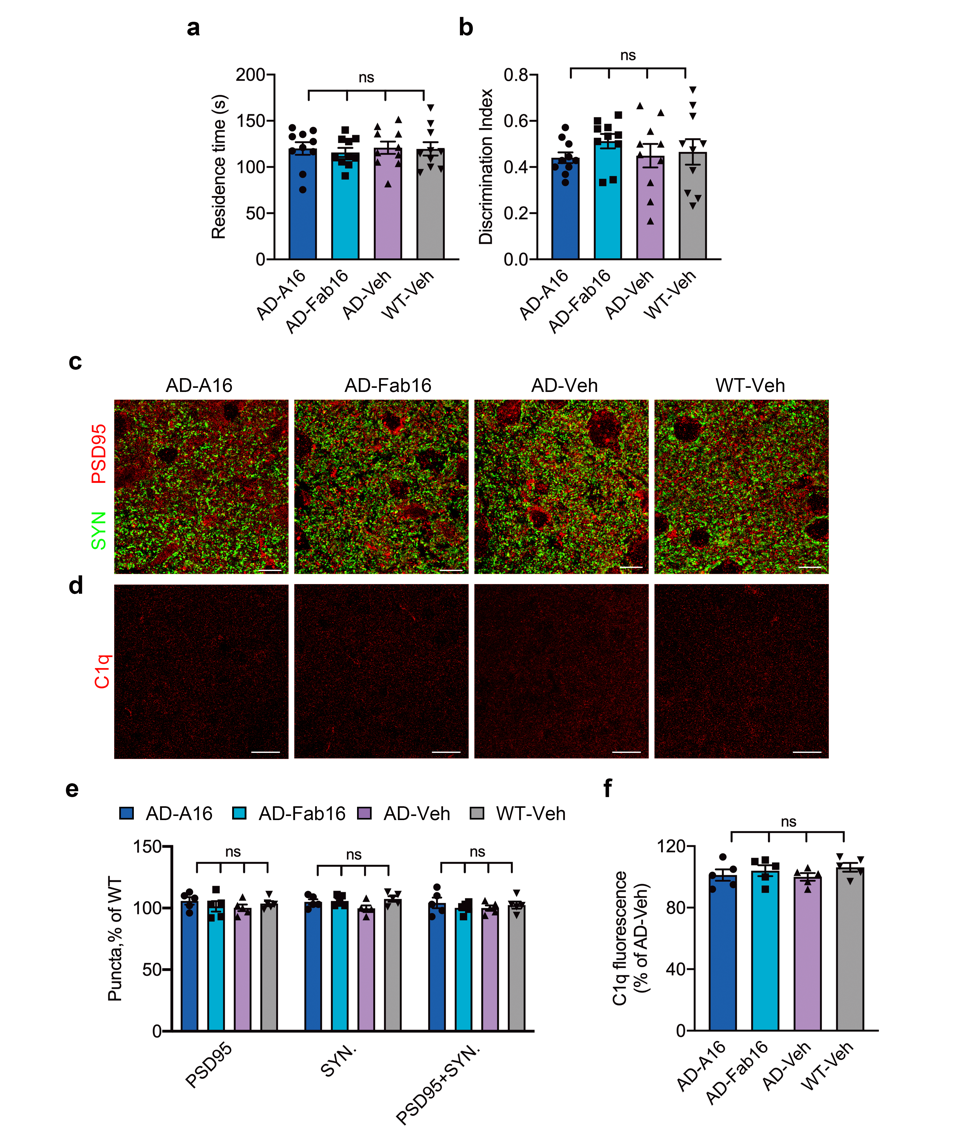
**

**Supplementary Fig.5. Full effector antibody A16 fails to induce memory deficits and synapse loss in 3-month-old APP/PS1 mice. a** The time spent by APP/PS1 or WT mice in the novel arm of Y-maze at 24 h post-treatment with A16, Fab16, or PBS (*n* = 10 mice). **b** Discrimination index of the mice in novel object recognition test (*n* = 10 mice). **c** Immunolabeling of colocalized PSD95 (red) and synaptophysin (green) puncta in the brains of APP/PS1 or WT mice treated with A16, Fab16, or PBS, at 48 h post-treatment. Scale bar, 20 μm. **d** Immunolabeling for C1q in the brains of APP/PS1 or WT mice treated with A16, Fab16, or PBS, at 48 h post-injection. Scale bar, 20 μm. **e** Quantification of synaptic puncta or their apposition in **c (***n* = 5 mice). **f** Quantification of C1q immunofluorescence intensity in **d** (n = 5 mice). Data are expressed as mean ± s.e.m. and were analyzed by one-way ANOVA with Tukey’s test. ns, not significant.


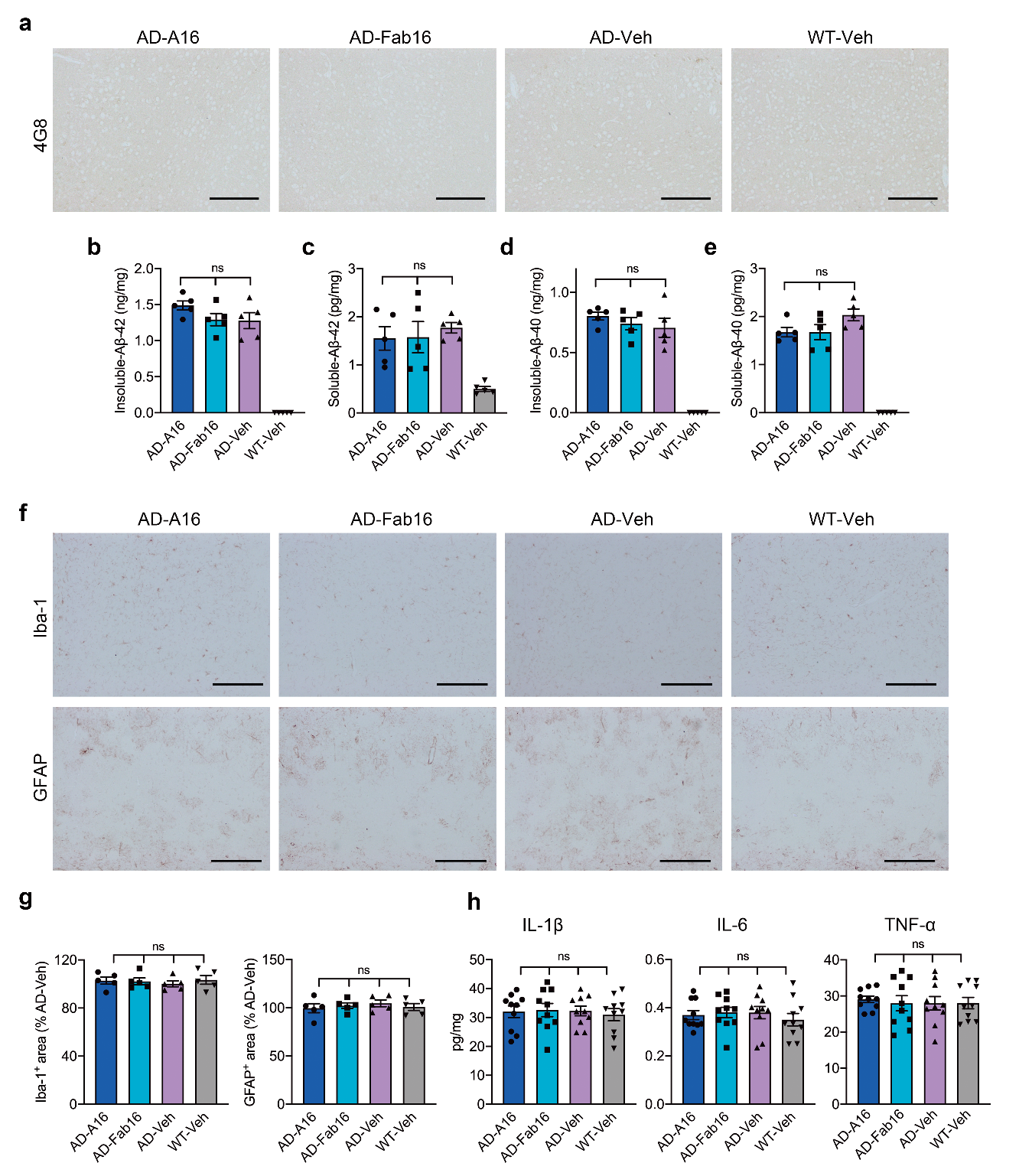


**Supplementary Fig. 6. Full effector antibody A16 fails to affect Aβ levels and inflammation in the brains of 3-month-old APP/PS1 mice. a** Detection of Aβ plaques by 4G8 immunolabeling in the brains of APP/PS1 or WT mice treated with A16, Fab16, or PBS, at 48 h post-injection. Scale bar, 200 μm. **b-e** Levels of insoluble Aβ42 (**b**), soluble Aβ42 (**c**), insoluble Aβ40 (**d**), and soluble Aβ40 (**e**) in brain lysates of APP/PS1 or WT mice treated with A16, Fab16, or PBS, at 48 h post-injection (*n* = 5 mice). **f** Detection of microglia and astrocytes by immunolabeling Iba-1 and GFAP in the brains of APP/PS1 or WT mice treated with A16, Fab16, or PBS, at 48 h post-injection. Scale bar, 200 μm. **g** Quantification of Iba-1-, GFAP-labeled area in **f** (*n* = 5 mice). **h** The levels of IL-1β, IL-6, and TNF-α in the brain lysates of APP/PS1 or WT mice treated with A16, Fab16, or PBS at 48 h post-injection (*n* = 10 mice). Data are expressed as mean ± s.e.m. and were analyzed by one-way ANOVA with Tukey’s test. ns, not significant.


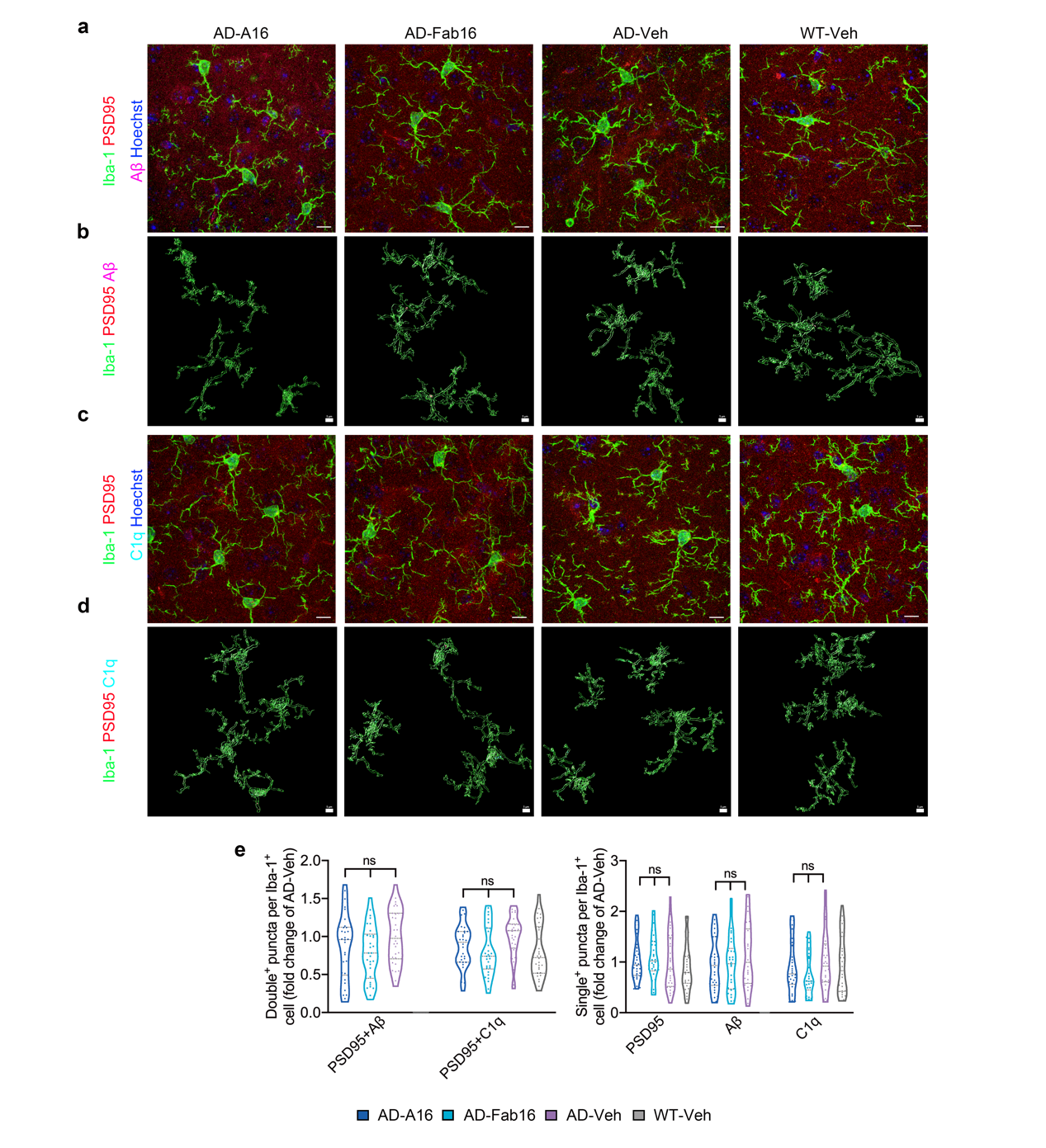


**Supplementary Fig. 7. Full effector antibody A16 fails to induce microglial engulfment of synapses in the brains of 3-month-old APP/PS1 mice. a** Representative images of the engulfed PSD95 (red) and Aβ (magenta) puncta within Iba-1^+^ (green) microglial cells in the brains of APP/PS1 or WT mice treated with A16, Fab16 or PBS, at 48 h post-injection. Scale bar, 10 μm. **b** 3D surface rendering of Iba-1^+^ microglia (green) containing engulfed PSD95 (red) and Aβ (magenta) puncta in **a**. Scale bar, 5 μm. **c** Representative images of the engulfed PSD95 (red) and C1q (cyan) puncta within Iba-1^+^ (green) microglial cells in the brains of APP/PS1 or WT mice treated with A16, Fab16, or PBS, at 48 h post-injection. Scale bar, 10 μm. **d** 3D surface rendering of Iba-1^+^ microglia (green) containing engulfed PSD95 (red) and C1q (cyan) puncta in **c**. Scale bar, 5 μm. **e** Quantification of colocalized PSD95 and Aβ puncta, PSD95 and C1q puncta, and PSD95, Aβ and C1q puncta, per Iba-1^+^ microglial cell (*n* = 30 microglial cells from 5 mice). Data are displayed as violin plots and were analyzed by one-way ANOVA with Tukey’s test. Dotted lines represent medians and interquartile ranges. ns, not significant.

**
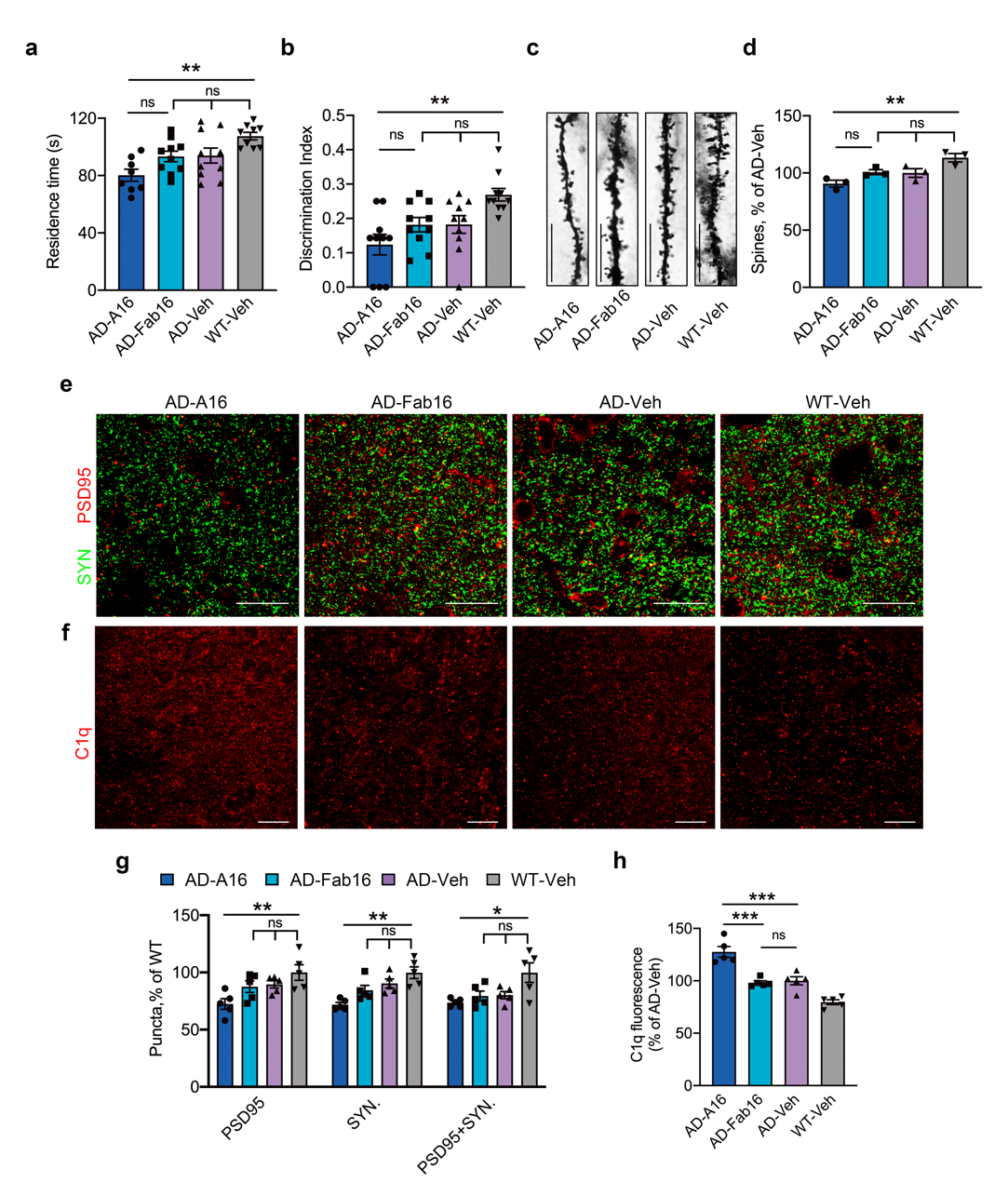
**

**Supplementary Fig. 8. Full effector antibody A16 induces memory deficits and synapse loss in 5-month-old APP/PS1 mice. a** The time spent by APP/PS1 or WT mice in the novel arm of Y-maze at 24 h post-treatment with A16, Fab16, or PBS (*n* = 10 mice). **b** Discrimination index of the mice in novel object recognition test (*n* = 10 mice). **c** Representative images of Golgi-stained dendrites from the cortex neurons of APP/PS1 or WT mice treated with A16, Fab16, or PBS, at 48 h post-treatment. Scale bar, 20 μm. **d** Quantification of dendritic spine density in **c** (*n* = 3 mice). **e** Immunolabeling of colocalized PSD95 (red) and synaptophysin (green) puncta in the brains of APP/PS1 or WT mice treated with A16, Fab16, or PBS, at 48 h post-treatment. Scale bar, 20 μm. **f** Immunolabeling for C1q in the brains of APP/PS1 or WT mice treated with A16, Fab16, or PBS, at 48 h post-treatment. Scale bar, 20 μm. **g** Quantification of synaptic puncta or their apposition in **e (***n* = 5 mice). **h** Quantification of C1q immunofluorescence intensity in **f** (n = 5 mice). Data are expressed as mean ± s.e.m. and were analyzed by one-way ANOVA with Tukey’s test. **P* < 0.05, ***P* < 0.01, ****P* < 0.001; ns, not significant.


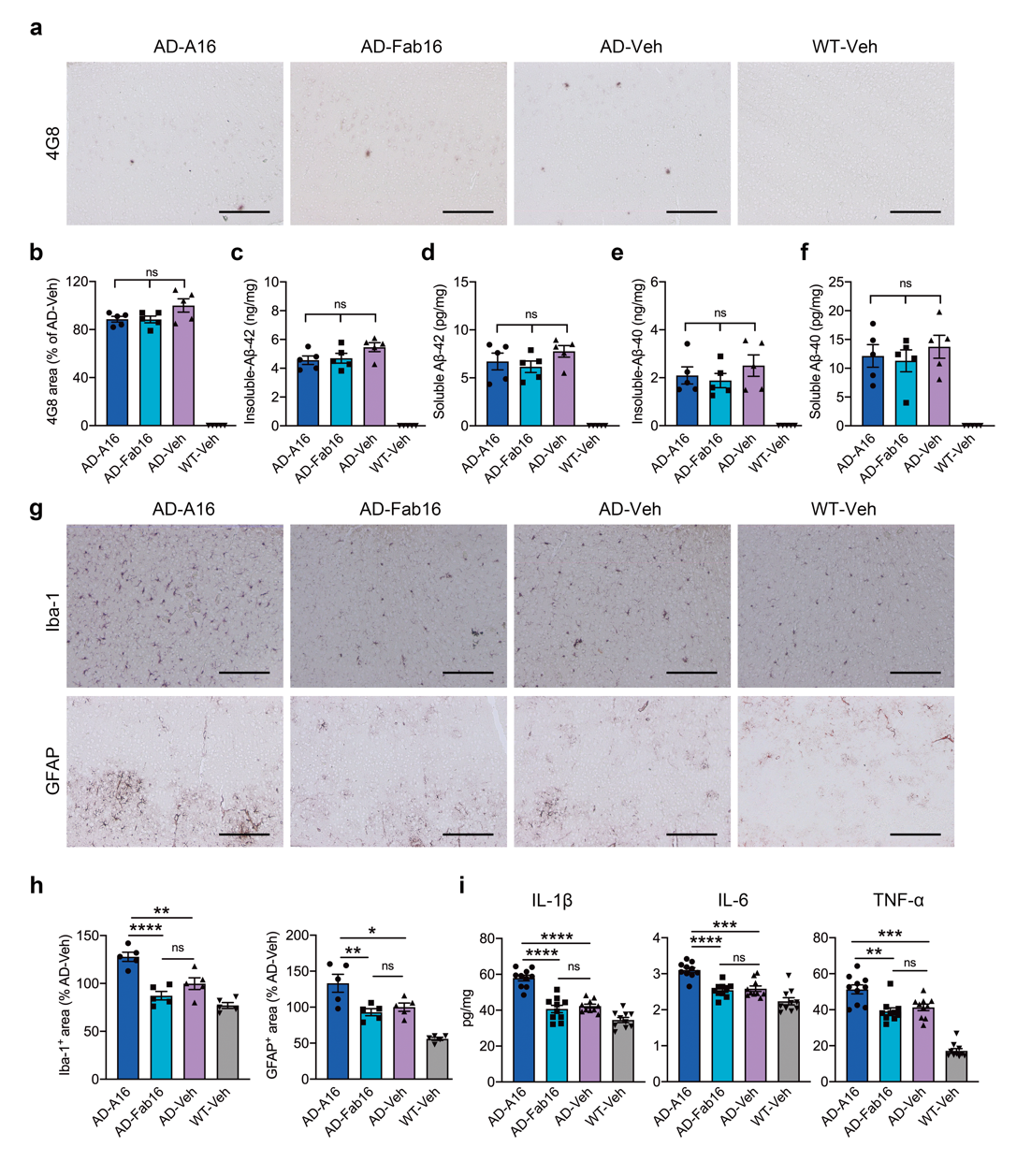


**Supplementary Fig. 9. Full effector antibody A16 fails to affect Aβ levels, but enhances inflammation in the brains of 5-month-old APP/PS1 mice. a** Detection of Aβ plaques by 4G8 immunolabeling in the brains of APP/PS1 or WT mice treated with A16, Fab16, or PBS, at 48 h post-injection. Scale bar, 200 μm. **b** Quantification of 4G8-labeled area in **a** (*n* = 5 mice). **c-f** Levels of insoluble Aβ42 (**c**), soluble Aβ42 (**d**), insoluble Aβ40 (**e**), and soluble Aβ40 (**f**) in brain lysates of APP/PS1 or WT mice treated with A16, Fab16, or PBS, at 48 h post-injection (*n* = 5 mice). **g** Detection of microglia and astrocytes by immunolabeling Iba-1 and GFAP in the brains of APP/PS1 or WT mice treated with A16, Fab16, or PBS, at 48 h post-injection. Scale bar, 200 μm. **h** Quantification of Iba-1-, GFAP-labeled area in **g** (*n* = 5 mice). **i** The levels of IL-1β, IL-6, and TNF-α in the brain lysates of APP/PS1 or WT mice treated with A16, Fab16, or PBS, at 48 h post-injection (*n* = 10 mice). Data are expressed as mean ± s.e.m. and were analyzed by one-way ANOVA with Tukey’s test. **P* < 0.05, ***P* < 0.01, ****P* < 0.001, *****P* < 0.0001; ns, not significant.

**
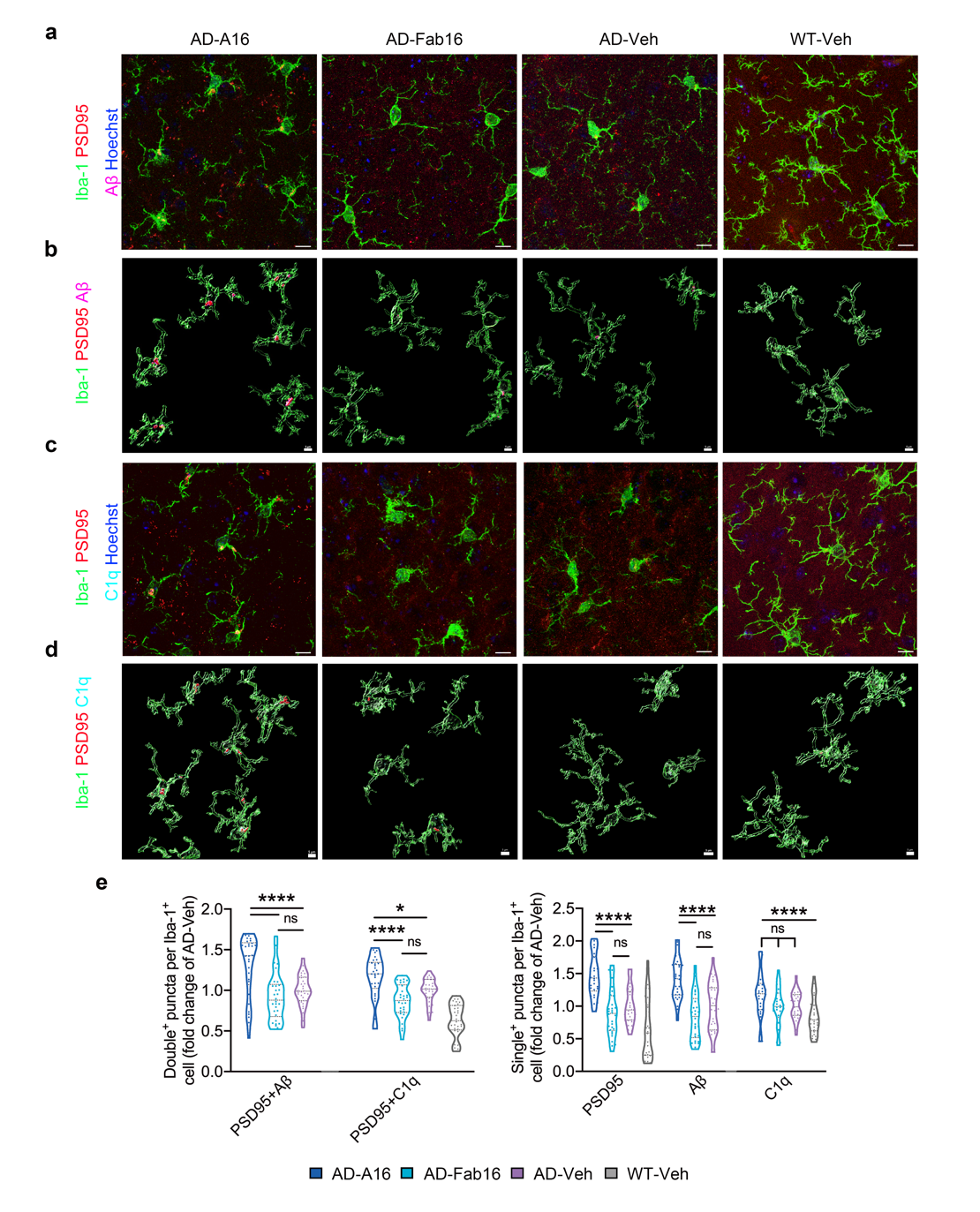
**

**Supplementary Fig. 10. Full effector antibody A16 induces microglial engulfment of synapses in the brains of 5-month-old APP/PS1 mice. a** Representative images of the engulfed PSD95 (red) and Aβ (magenta) puncta within Iba-1^+^ (green) microglial cells in the brains of APP/PS1 or WT mice treated with A16, Fab16, or PBS, at 48 h post-injection. Scale bar, 10 μm. **b** 3D surface rendering of Iba-1^+^ microglia (green) containing engulfed PSD95 (red) and Aβ (magenta) puncta in **a**. Scale bar, 5 μm. **c** Representative images of the engulfed PSD95 (red) and C1q (cyan) puncta within Iba-1^+^ (green) microglial cells in the brains of APP/PS1 or WT mice treated with A16, Fab16, or PBS, at 48 h post-injection. Scale bar, 10 μm. **d** 3D surface rendering of Iba-1^+^ microglia (green) containing engulfed PSD95 (red) and C1q (cyan) puncta in **c**. Scale bar, 5 μm. **e** Quantification of colocalized PSD95 and Aβ puncta, PSD95 and C1q puncta, and PSD95, Aβ and C1q puncta, per Iba-1^+^ microglial cell (n =30 microglia cells from 5 mice). Data are displayed as violin plots and were analyzed by one-way ANOVA with Tukey’s test. Dotted lines represent medians and interquartile ranges. *P < 0.05, ****P < 0.0001; ns, not significant.

**
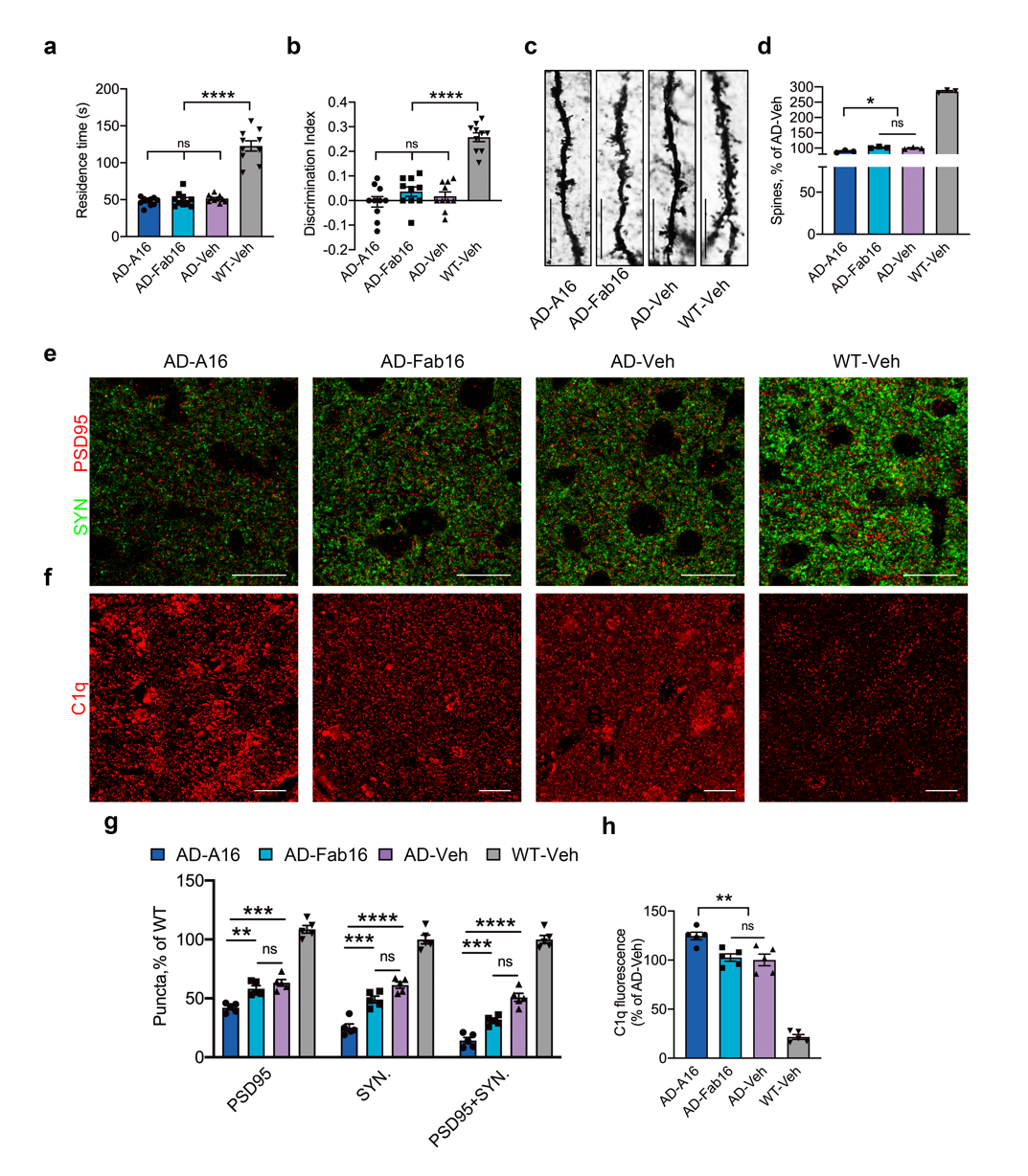
**

**Supplementary Fig. 11. Full effector antibody A16 induces synapse loss in 10-month-old APP/PS1 mice. a** The time spent by APP/PS1 or WT mice in the novel arm of Y-maze at 24 h post-treatment with A16, Fab16, or PBS (*n* = 10 mice). **b** Discrimination index of the mice in novel object recognition test (*n* = 10 mice). **c** Representative images of Golgi-stained dendrites from the cortex neurons of APP/PS1 or WT mice treated with A16, Fab16, or PBS, at 48 h post-treatment. Scale bar, 20 μm. **d** Quantification of dendritic spine density in **c** (*n* = 3 mice). **e** Immunolabeling of colocalized PSD95 (red) and synaptophysin (green) puncta in the cortex of APP/PS1 or WT mice treated with A16, Fab16, or PBS at 48 h post-treatment. Scale bar, 20 μm. **f** Immunolabeling for C1q in the cortex of APP/PS1 or WT mice treated with A16, Fab16, or PBS, at 48 h post-injection after the first treatment. Scale bar, 20 μm. **g** Quantification of synaptic puncta or their apposition in **e (***n* = 5 mice). **h** Quantification of C1q immunofluorescence intensity in **f** (n = 5 mice). Data are expressed as mean ± s.e.m. and were analyzed by one-way ANOVA with Tukey’s test. **P* < 0.05, ***P* < 0.01, ****P* < 0.001, *****P* < 0.0001; ns, not significant.

**
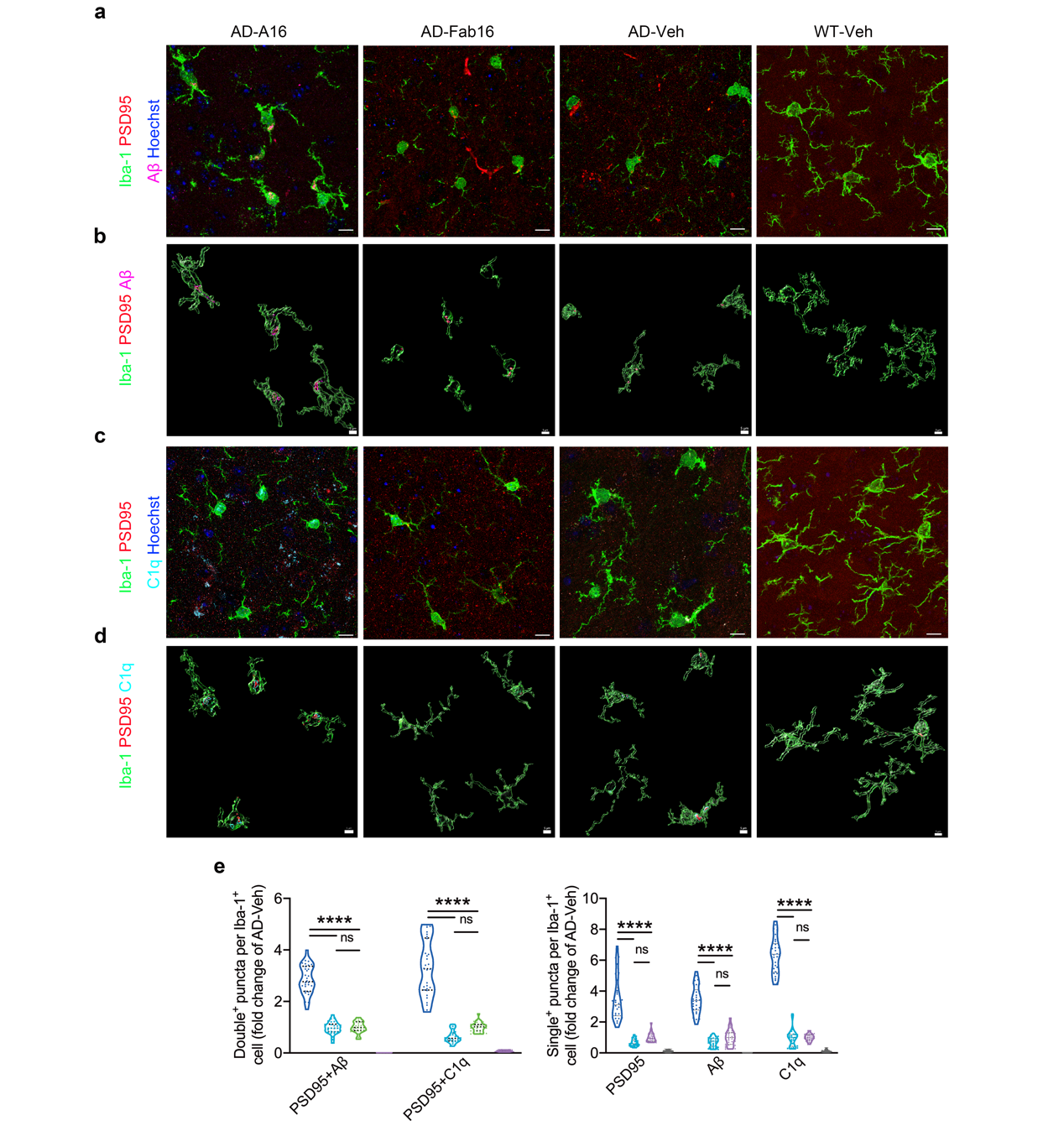
**

**Supplementary Fig. 12. Full effector antibody A16 induces microglial engulfment of synapses in the brains of 10-month-old APP/PS1 mice. a** Representative images of the engulfed PSD95 (red) and Aβ (magenta) puncta within Iba-1^+^ (green) microglial cells in the brains of APP/PS1 or WT mice treated with A16, Fab16, or PBS, at 48 h post-injection. Scale bar, 10 μm. **b** 3D surface rendering of Iba-1^+^ microglia (green) containing engulfed PSD95 (red) and Aβ (magenta) puncta in **a**. Scale bar, 5 μm. **c** Representative images of the engulfed PSD95 (red) and C1q (cyan) puncta within Iba-1^+^ (green) microglial cells in the brains of APP/PS1 or WT mice treated with A16, Fab16, or PBS, at 48 h post-injection. Scale bar, 10 μm. **d** 3D surface rendering of Iba-1^+^ microglia (green) containing engulfed PSD95 (red) and C1q (cyan) puncta in **c**. Scale bar, 5 μm. **e** Quantification of colocalized PSD95 and Aβ puncta, PSD95 and C1q puncta, and PSD95, Aβ and C1q puncta, per Iba-1^+^ microglial cell (n =30 microglia cells from 5 mice). Data are displayed as violin plots and were analyzed by one-way ANOVA with Tukey’s test. Dotted lines represent medians and interquartile ranges. ****P < 0.0001; ns, not significant.

**
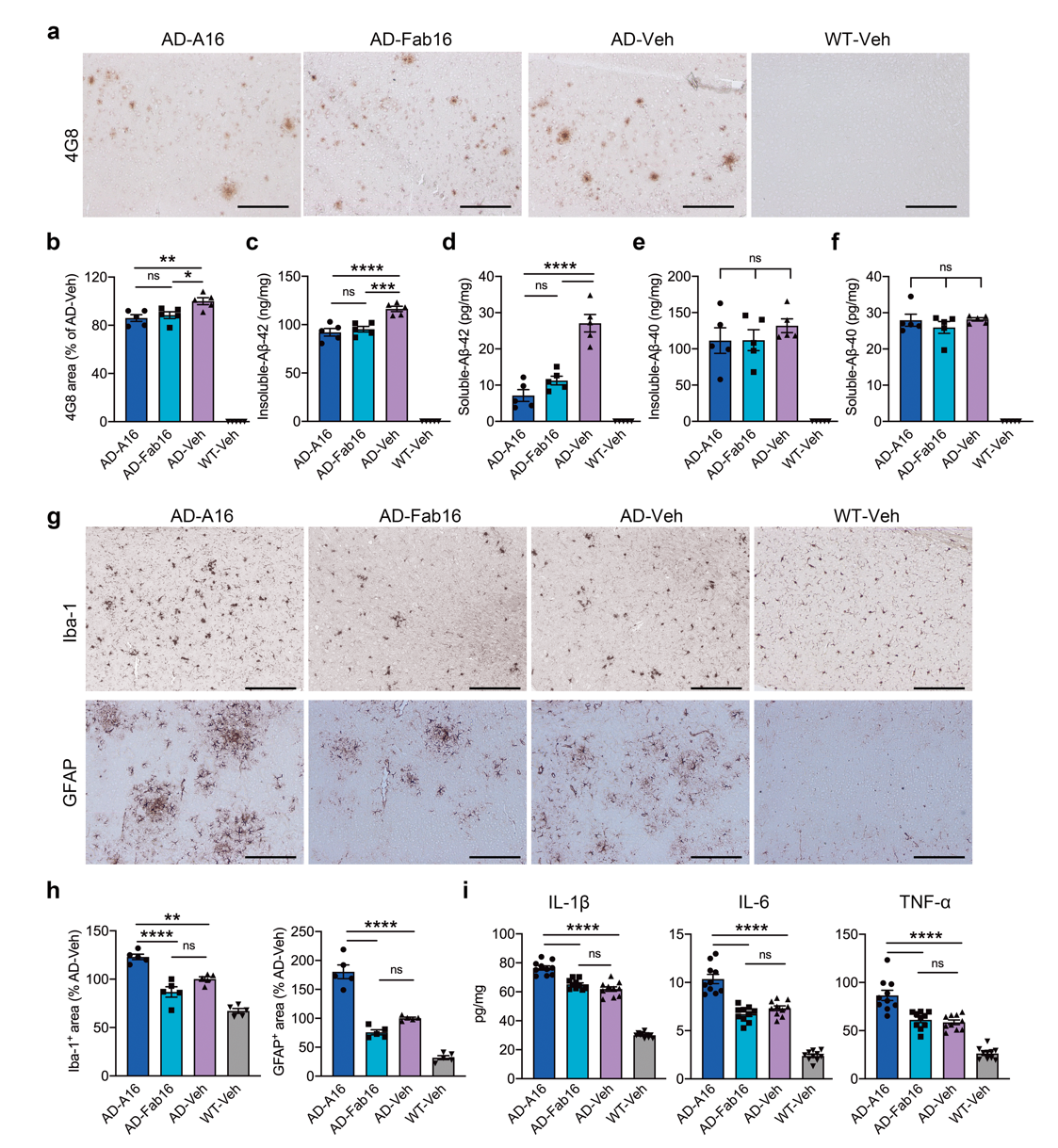
**

**Supplementary Fig. 13. Full effector antibody A16 reduces Aβ burden, but enhances inflammation in the brains of 10-month-old APP/PS1 mice. a** Detection of Aβ plaques by 4G8 immunolabeling in the brains of APP/PS1 or WT mice treated with A16, Fab16, or PBS, at 48 h post-injection. Scale bar, 200 μm. **b** Quantification of 4G8-labeled area in **a** (*n* = 5 mice). **c-f** Levels of insoluble Aβ42 (**c**), soluble Aβ42 (**d**), insoluble Aβ40 (**e**), and soluble Aβ40 (**f**) in brain lysates of APP/PS1 or WT mice treated with A16, Fab16, or PBS, at 48 h post-injection (*n* = 5 mice). **g** Detection of microglia and astrocytes by immunolabeling Iba-1 and GFAP in the brains of APP/PS1 or WT mice treated with A16, Fab16, or PBS, at 48 h post-injection. Scale bar, 200 μm. **h** Quantification of Iba-1-, GFAP-labeled area in **g** (*n* = 5 mice). **i** The levels of IL-1β, IL-6, and TNF-α in the brain lysates of APP/PS1 or WT mice treated with A16, Fab16, or PBS, at 48 h post-injection (*n* = 10 mice). Data are expressed as mean ± s.e.m. and were analyzed by one-way ANOVA with Tukey’s test. **P* < 0.05, ***P* < 0.01, ****P* < 0.001, *****P* < 0.0001; ns, not significant**.**

**
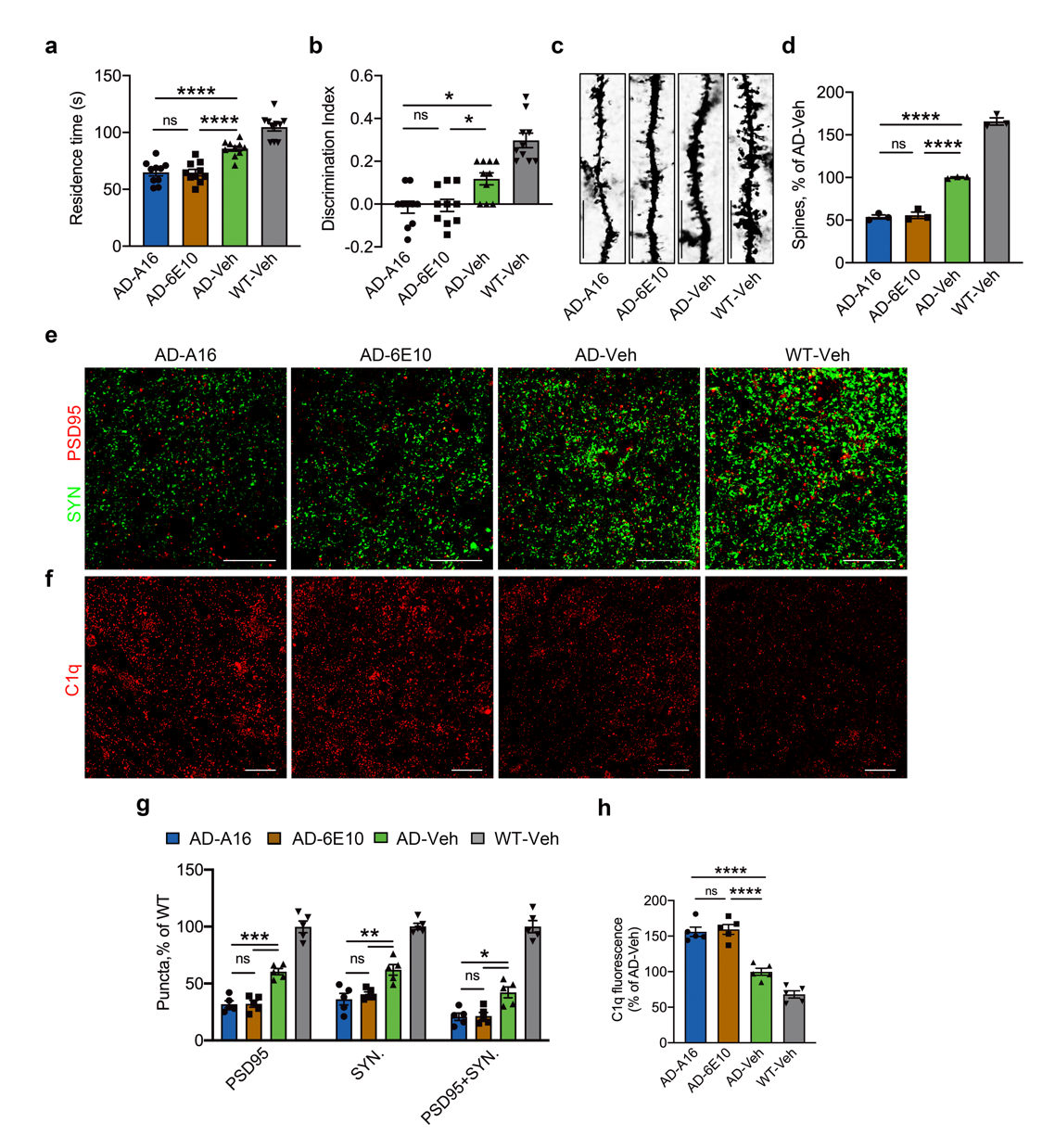
**

**Supplementary Fig. 14. Full effector anti-Aβ antibody A16 and 6E10 induces memory deficits and synapse loss in 6-month-old APP/PS1 mice. a** The time spent by APP/PS1 or WT mice in the novel arm of Y-maze at 24 h post-treatment with A16, 6E10, or PBS (*n* = 10 mice). **b** Discrimination index of the mice in novel object recognition test (*n* = 10 mice). **c** Representative images of Golgi-stained dendrites from the cortex neurons of APP/PS1 or WT mice treated with A16, 6E10, or PBS, at 48 h post-treatment. Scale bar, 20 μm. **d** Quantification of dendritic spine density in **c** (*n* = 3 mice). **e** Immunolabeling of colocalized PSD95 (red) and synaptophysin (green) puncta in the cortex of APP/PS1 or WT mice treated with A16, 6E10, or PBS, at 48 h post-treatment. Scale bar, 20 μm. **f** Immunolabeling for C1q in the cortex of APP/PS1 or WT mice treated with A16, 6E10, or PBS, at 48 h post-injection after the first treatment. Scale bar, 20 μm. **g** Quantification of synaptic puncta or their apposition in **e (***n* = 5 mice). **h** Quantification of C1q immunofluorescence intensity in **f** (n = 5 mice). Data are expressed as mean ± s.e.m. and were analyzed by one-way ANOVA with Tukey’s test. **P* < 0.05, ***P* < 0.01, ****P* < 0.001, *****P* < 0.0001; ns, not significant.


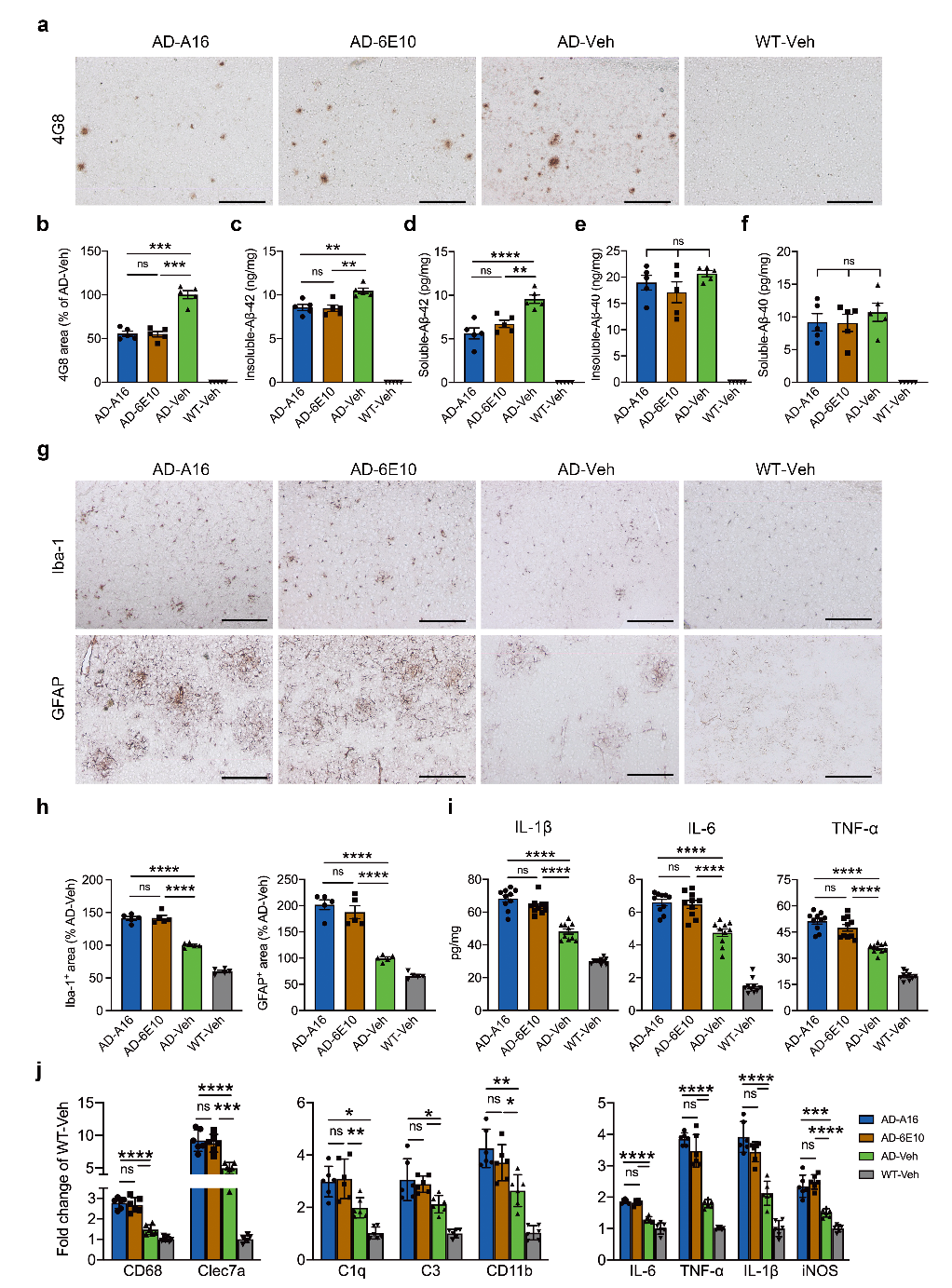


**Supplementary Fig. 15. Full effector anti-Aβ antibody A16 and 6E10 reduces Aβ burden, but enhances inflammation in the brains of 6-month-old APP/PS1 mice. a** Detection of Aβ plaques by 4G8 immunolabeling in the brains of APP/PS1 or WT mice treated with A16, 6E10, or PBS, at 48 h post-injection. Scale bar, 200 μm. **b** Quantification of 4G8-labeled area in **a** (*n* = 5 mice). **c-f** Levels of insoluble Aβ42 (**c**), soluble Aβ42 (**d**), insoluble Aβ40 (**e**), and soluble Aβ40 (**f**) in brain lysates of APP/PS1 or WT mice treated with A16, 6E10, or PBS, at 48 h post-injection (*n* = 5 mice). **g** Detection of microglia and astrocytes by immunolabeling Iba-1 and GFAP in the brains of APP/PS1 or WT mice treated with A16, 6E10, or PBS, at 48 h post-injection. Scale bar, 200 μm. **h** Quantification of Iba-1-, GFAP-labeled area in **g** (*n* = 5 mice). **i** The levels of IL-1β, IL-6, and TNF-α in the brain lysates of APP/PS1 or WT mice treated with A16, 6E10 or PBS, at 48 h post-injection (*n* = 10 mice). **j** The expression signatures of inflammation-related gene (CD68, Clec7a), complement-related gene (C1q, C3, CD11b), and inflammatory factor-related gene (IL-6, TNF-α, IL-1β, iNOS) in microglial cells of APP/PS1 mice were analyzed by qPCR (n = 6 mice). Data are expressed as mean ± s.e.m. and were analyzed by one-way ANOVA with Tukey’s test. **P* < 0.05, ***P* < 0.01, ****P* < 0.001, *****P* < 0.0001; ns, not significant.


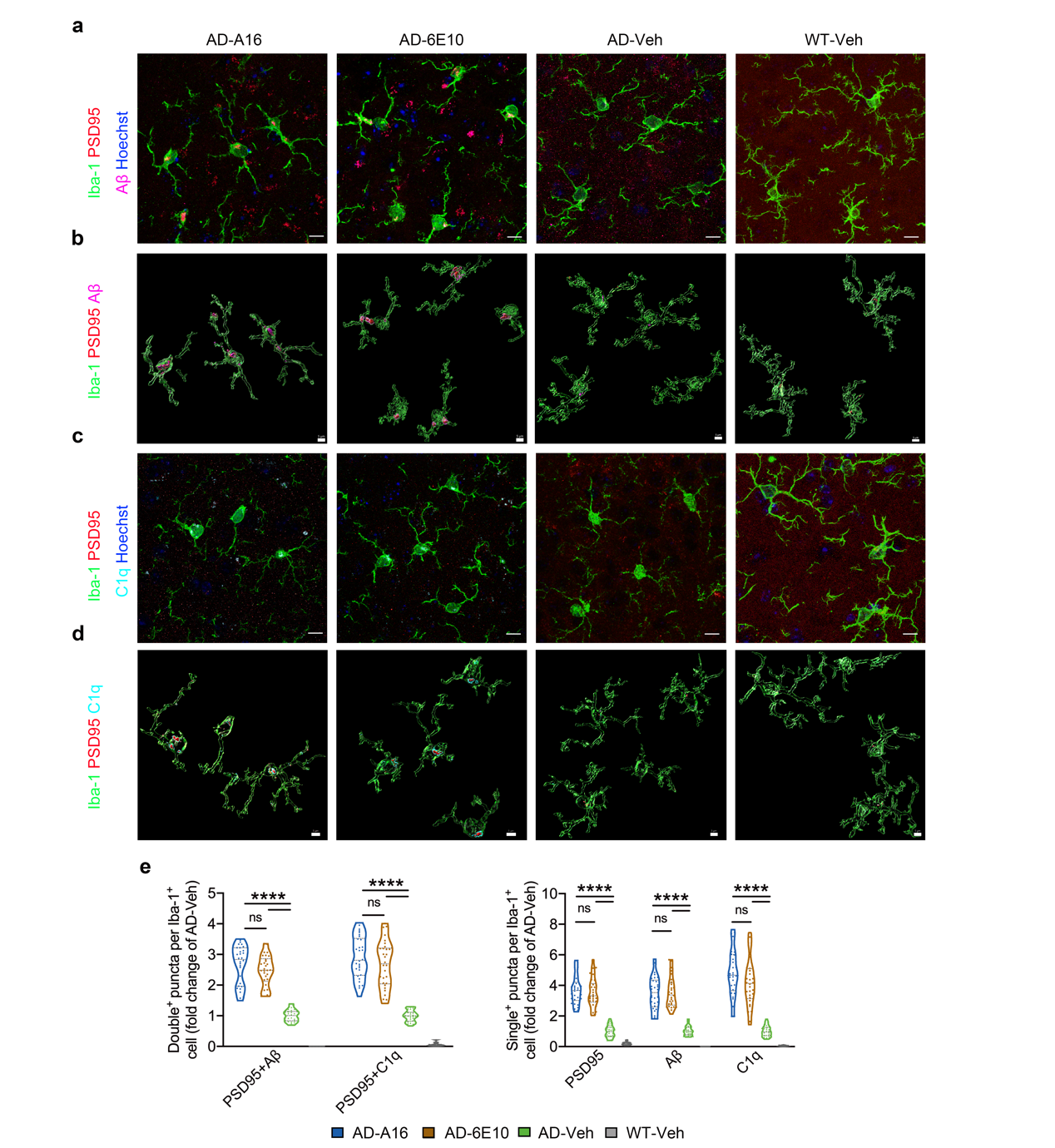


**Supplementary Fig. 16. Full effector anti-Aβ antibody A16 and 6E10 induces microglial engulfment of synapses in the brains of 6-month-old APP/PS1 mice. a** Representative images of the engulfed PSD95 (red) and Aβ (magenta) puncta within Iba-1^+^ (green) microglial cells in the brains of APP/PS1 or WT mice treated with A16, 6E10 or PBS at 48 h post-injection. Scale bar, 10 μm. **b** 3D surface rendering of Iba-1^+^ microglia (green) containing engulfed PSD95 (red) and Aβ (magenta) puncta in **a**. Scale bar, 5 μm. **c** Representative images of the engulfed PSD95 (red) and C1q (cyan) puncta within Iba-1^+^ (green) microglial cells in the brains of APP/PS1 or WT mice treated with A16, 6E10, or PBS, at 48 h post-injection. Scale bar, 10 μm. **d** 3D surface rendering of Iba-1^+^ microglia (green) containing engulfed PSD95 (red) and C1q (cyan) puncta in **c**. Scale bar, 5 μm. **e** Quantification of colocalized PSD95 and Aβ puncta, PSD95 and C1q puncta, and PSD95, Aβ and C1q puncta, per Iba-1^+^ microglial cell (n = 30 microglia cells from 5 mice). Data are displayed as violin plots and were analyzed by one-way ANOVA with Tukey’s test. Dotted lines represent medians and interquartile ranges. ****P < 0.0001; ns, not significant.


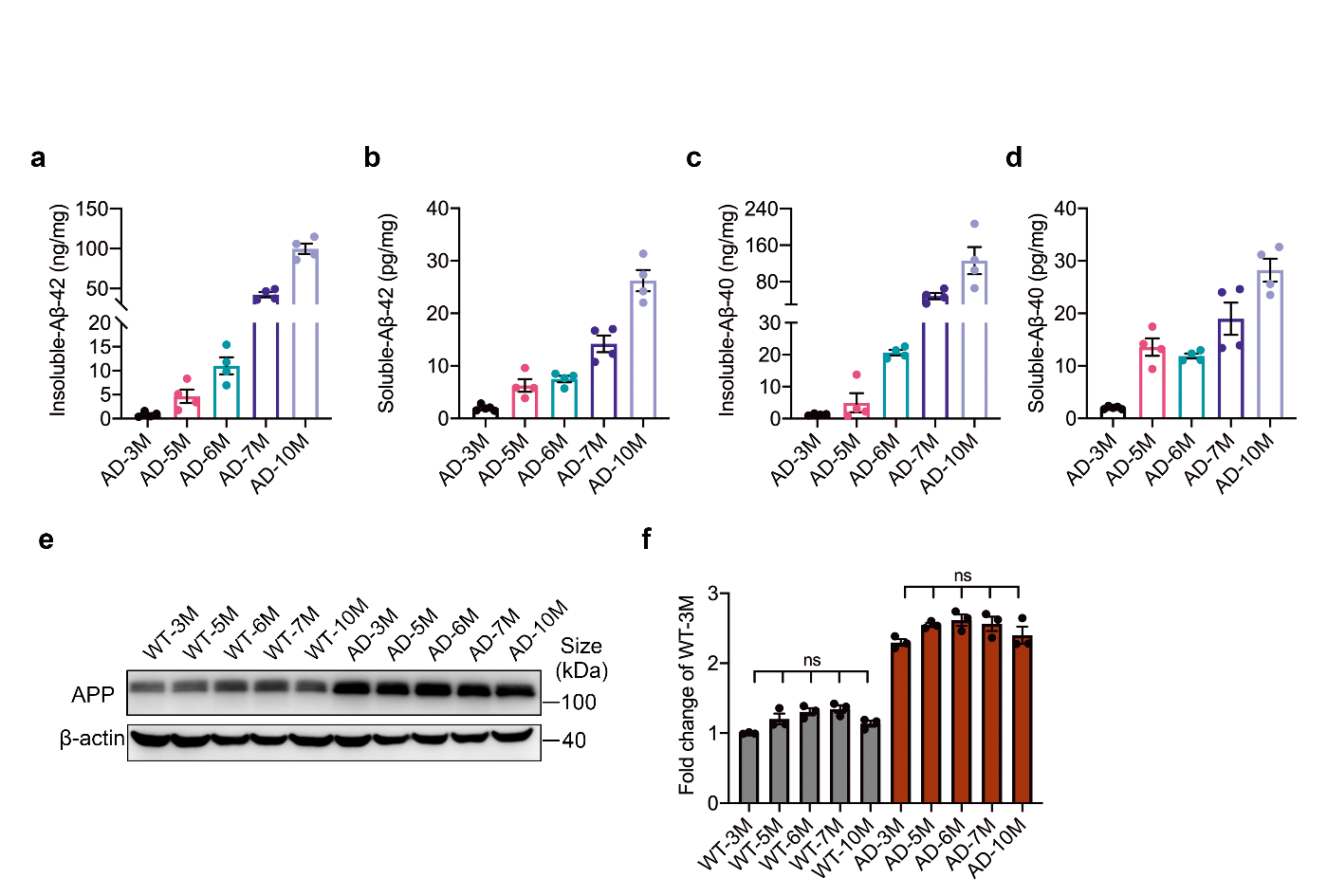


**Supplementary Fig. 17. The levels of Aβ and APP in the brain lysates of 3 to 10-month-old APP/PS1 mice. a-d** Levels of insoluble Aβ42 (**a**), soluble Aβ42 (**b**), insoluble Aβ40 (**c**), and soluble Aβ40 (**d**) in brain lysates of 3-, 5-, 6-, 7-, 10-month-old APP/PS1 mice (*n* = 4 mice). **e** Western blot analysis of APP levels in the brain lysates of 3-, 5-, 6-, 7-, 10-month-old APP/PS1 or WT mice (*n* = 3 mice). **f** Quantitation of APP levels in **e** (*n* = 3 mice). Data are expressed as mean ± s.e.m. and were analyzed by one-way ANOVA with Tukey’s test. ns, not significant.


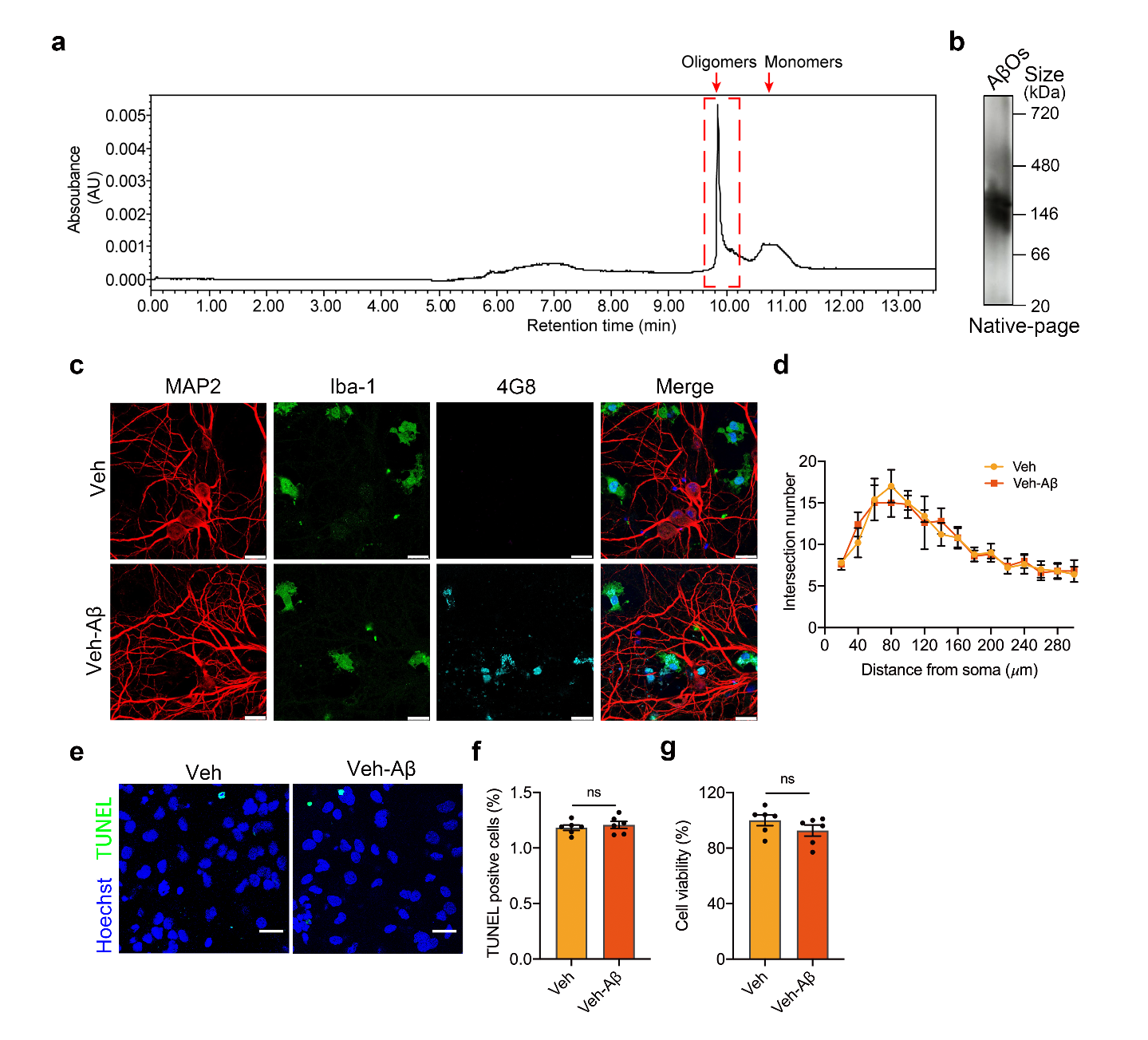


**Supplementary Fig. 18. Aβ oligomer preparation and the toxicity of 500 nM Aβ oligomers. a** Aβ oligomer preparation by size exclusion chromatography. **b** Oligomer native gel electrophoresis visualized by western-blotting using 6E10 antibody. **c** Immunolabeling of MAP2 (red), Iba-1(green), and 4G8 (cyan) in neuron-microglia cocultures treated with 500 nM AβOs. Scale bar, 25 μm. **d** Sholl analysis of the dendritic branching in primary neuronal cultures treated with 500 nM AβOs overnight. 40 neurons per culture from three independent cultures were used for the analysis. **e** Staining of TUNEL (green) and Hoechst (Blue) in primary neuron cultures. Scale bar, 25 μm. **f** Quantification of the number of TUNEL^+^ cells in primary neuronal cultures in **e** (*n* = 6 biological replicates over three independent experiments). **g** The viability of primary neurons treated with 500 nM AβOs for 12 h was determined using MTT assay (*n* = 6 biological replicates over three independent experiments). Data are expressed as mean ± s.e.m. and were analyzed by student’s t-test. ns, not significant.


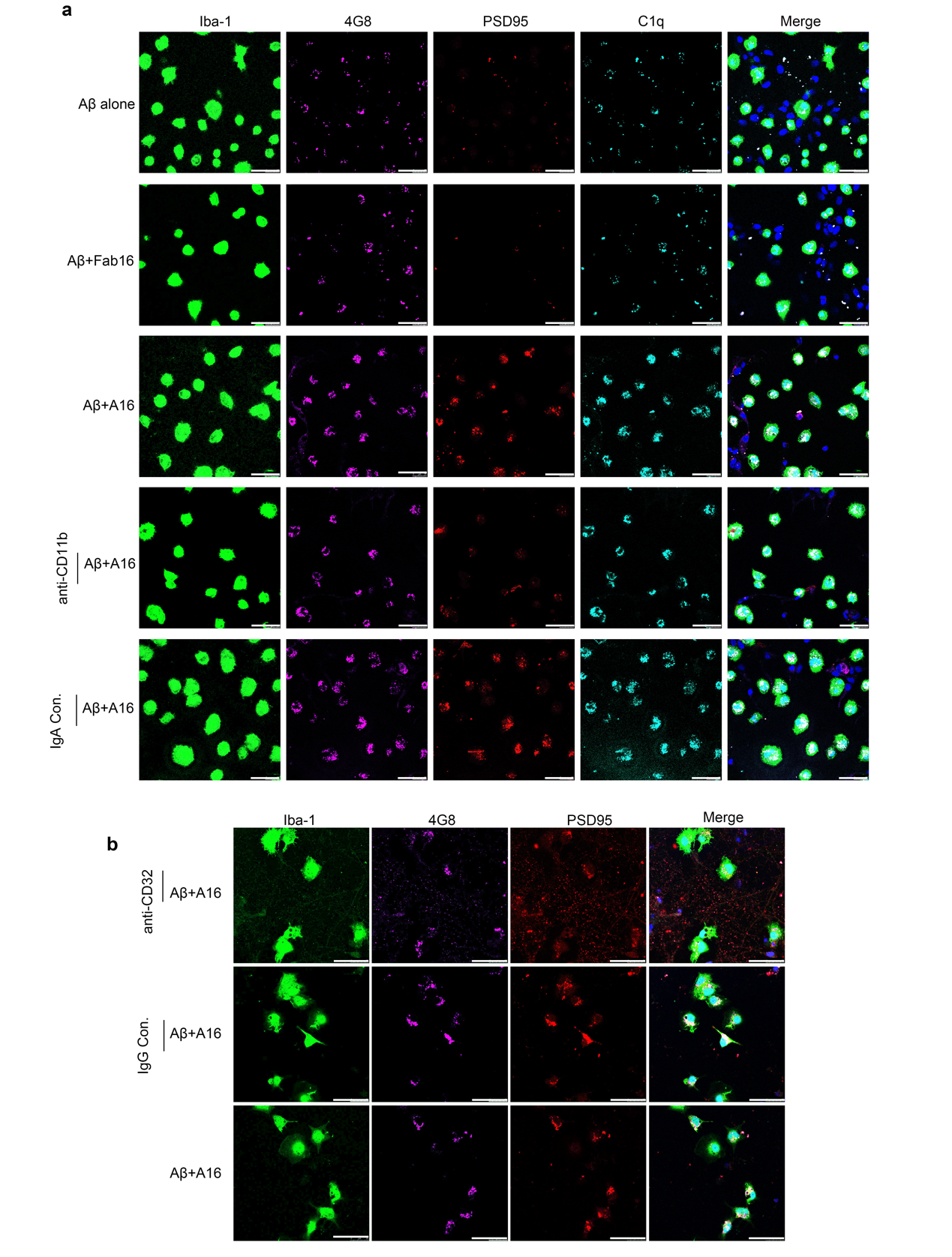


**Supplementary Fig. 19. Full effector antibody A16 but not effector-less antibody Fab16 induces microglial engulfment of synapses *in vitro*. a** Immunolabeling of Aβ (magenta), PSD95 (red), C1q (cyan) and Iba-1 (green) in neuron-microglia cocultures treated with A16 or Fab16 in presence of AβOs. Microglia cells were pretreated with anti-CD11b functional antibody or its isotype control IgA, respectively. Scale bars: 50 μm. **b** Immunolabeling of engulfed PSD95 (red) and Aβ (magenta) within Iba-1^+^ (green) microglial cells in neuron-microglia cocultures treated with A16 in presence of AβOs. Microglia cells were pretreated with anti-CD32 functional antibody or its isotype control IgG, respectively. Scale bar, 50 μm.


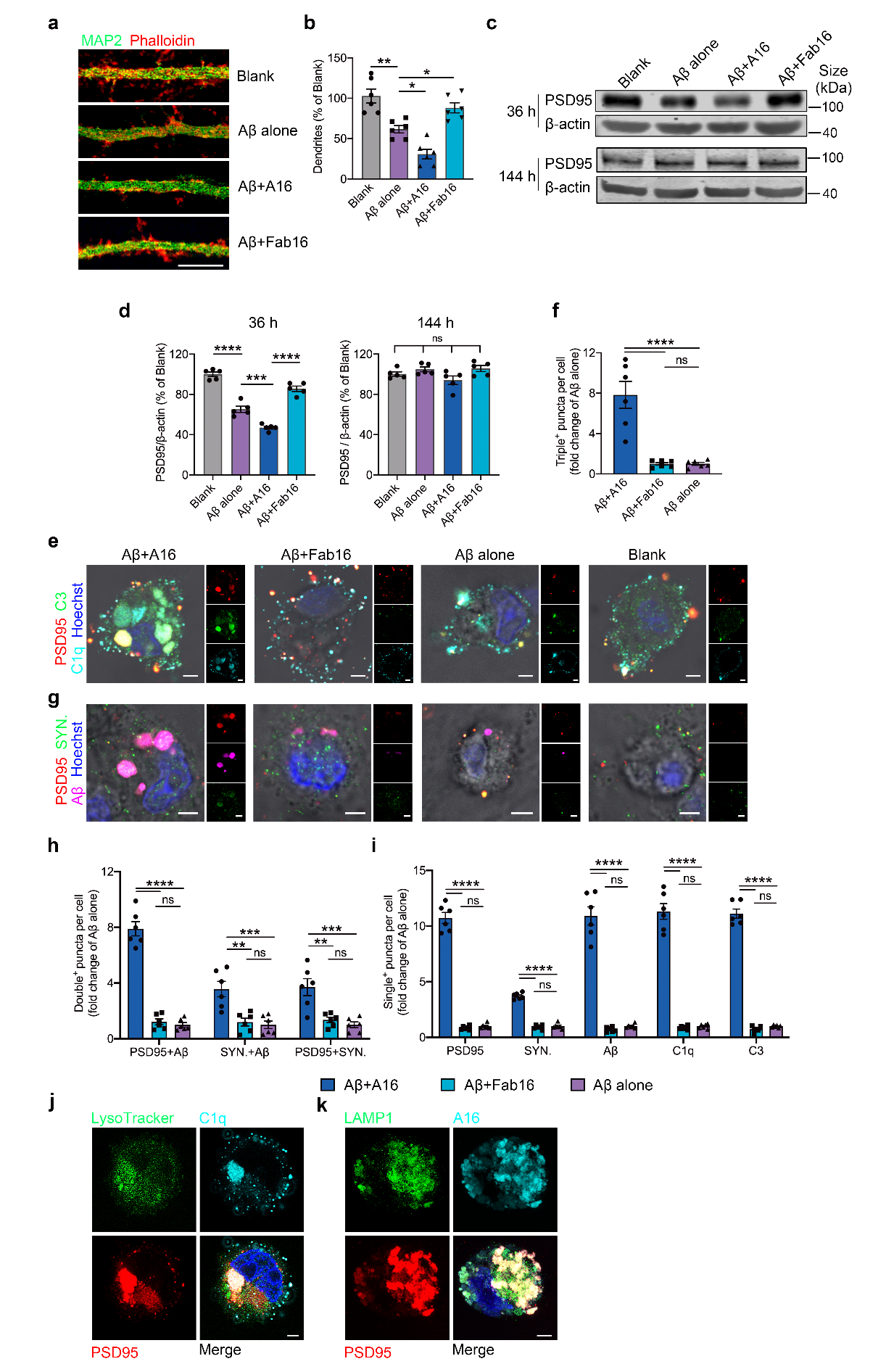


**Supplementary Fig. 20. Full effector antibody A16 mediates microglial synapse engulfment *in vitro.* a** Representative images of phalloidin (red)-stained dendritic spines in neurons (MAP2, green) cocultured with microglia and treated with A16 or Fab16 in the presence of AβOs. Scale bar, 25 μm. **b** Quantification of dendritic spine density in **a** (*n* = 6). **c** The neuron-microglia cocultures were treated with A16 or Fab16 in the presence of AβOs for 12 h, then the medium was replaced to fresh medium and the cocultures were further incubated for 36 h or 144 h. PSD95 levels in the cocultured neurons were detected using western-blotting. **d** Quantitation of PSD95 immunoblots in **c** (*n* = 5). **e** Immunolabeling of PSD95 (red), C3 (green), and C1q (cyan) in microglial cells in cocultures. Scale bar, 2.5 μm. **f** Quantification of colocalized PSD95, C3, and C1q puncta per cell in **e** (*n* = 6). **g** Immunolabeling of PSD95 (red), Aβ (magenta), and synaptophysin (green) in microglial cells in cocultures. Scale bar, 2.5 μm. **h** Quantification of colocalized PSD95 and Aβ puncta, synaptophysin and Aβ puncta, and PSD95 and synaptophysin puncta per cell in **g** (*n* = 6). **i** Quantification of PSD95, synaptophysin, Aβ, C1q, and C3 puncta per cell in **e** and **g** (*n* = 6). **j** Immunolabeling of colocalized PSD95 (red), LysoTracker (green), and C1q (cyan) in microglial cells in cocultures treated with A16 in the presence of AβOs. Scale bar, 2.5 μm. **k** Immunolabeling of PSD95 (red), LAMP1 (green), and A16 (cyan) in microglia cells in cocultures treated with A16 in the presence of AβOs. Scale bar, 5 μm. All experiments in **b**, **d**, **f**, **h**, and **i** were performed as biological replicates over three independent experiments. Data are expressed as mean ± s.e.m. and were analyzed by one-way ANOVA with Tukey’s test. **P* < 0.05, ***P* < 0.01, ****P* < 0.001, *****P* < 0.0001; ns, not significant.

**
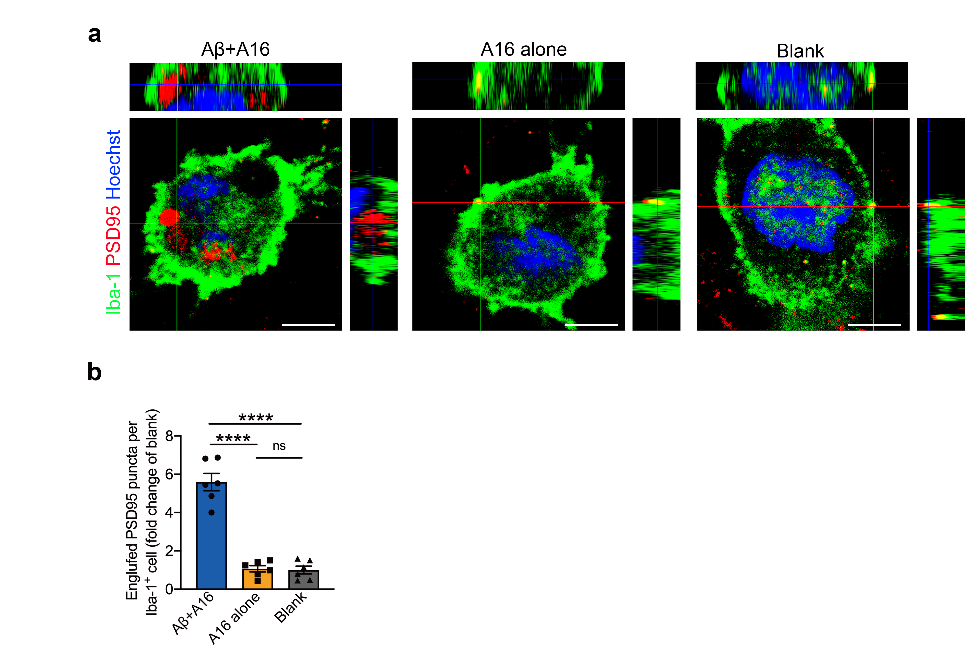
**

**Supplementary Fig. 21. Full effector antibody A16-triggered microglial engulfment of synapses is Aβ-dependent. a** Immunolabeling of PSD95 (red) and Iba-1 (green) in neuron-microglia cocultures treated with A16 in the absence or presence of AβOs. Scale bar, 5 μm. **b** Quantification of PSD95 puncta engulfed by Iba-1^+^ microglial cells in **a** (*n* = 6 biological replicates over three independent experiments). Data are expressed as mean ± s.e.m. and were analyzed by one-way ANOVA with Tukey’s test. *****P* < 0.0001; ns, not significant.

**
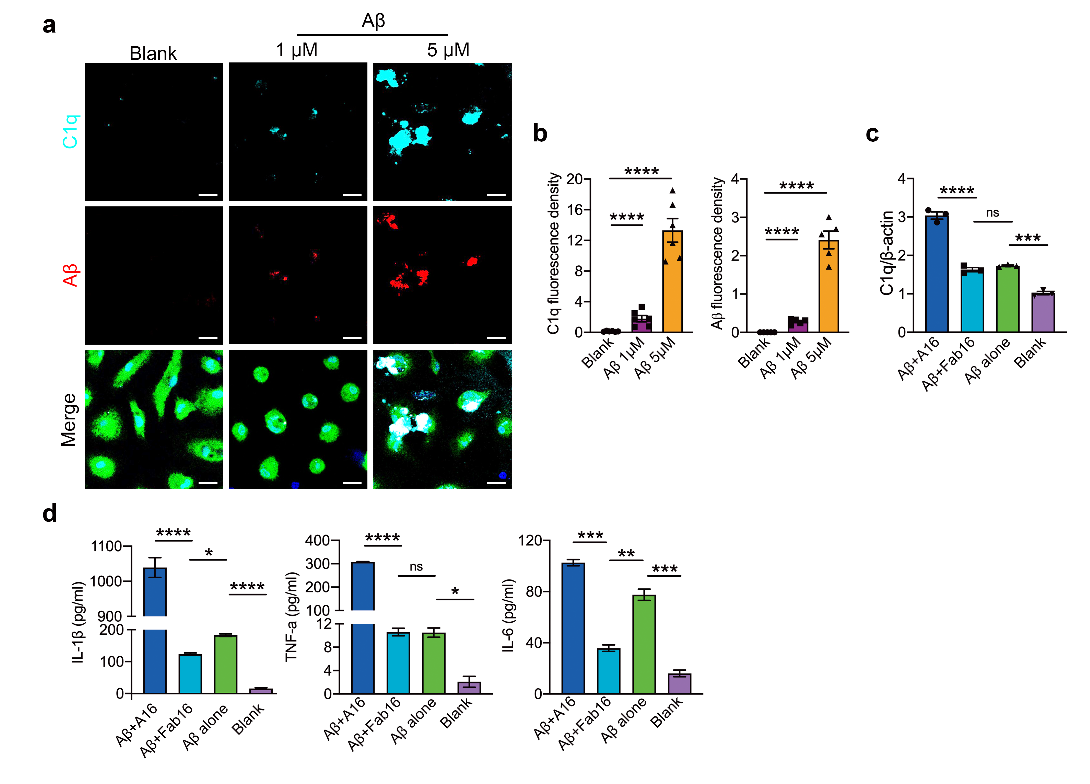
**

**Supplementary Fig. 22.** **C1q level is increased upon AβO challenge in microglia *in vitro*. a** Immunolabeling of C1q (cyan) and Aβ (red) in Iba-1^+^ (green) microglial cells in the presence of 1 or 5 μM AβOs. Scale bar, 5 μm. **b** Quantification of the fluorescence density of C1q and Aβ in Iba-1^+^ microglial cells in **a** (*n* = 6 biological replicates over three independent experiments). **c** qPCR analysis of C1q expression in the microglial cell cultures treated with A16 or Fab16 in the presence of AβOs (*n* = 3 independent experiments). **d** The levels of IL-1β, TNF-α, and IL-6 in microglial cell cultures treated with A16 or Fab16 in the presence of AβOs (*n* = 3 independent experiments). Data are expressed as mean ± s.e.m. and were analyzed by one-way ANOVA with Tukey’s test. **P* < 0.05, ***P* < 0.01, ****P* < 0.001, *****P* < 0.0001; ns, not significant.

**
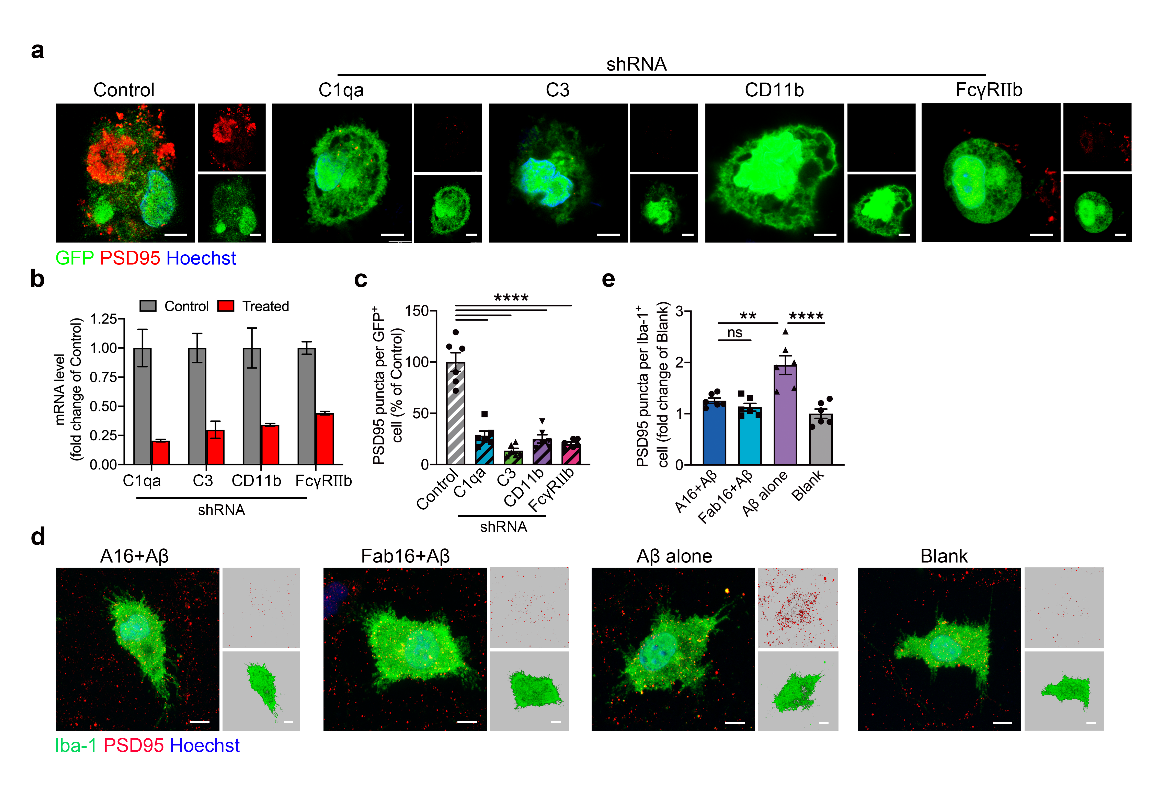
**

**Supplementary Fig. 23. CR3 and FcγRIIb mediate microglial engulfment of synapses. a** Immunolabeling of engulfed PSD95 (red) in *C1qa-, C3-, CD11b-, or FcγRIIb-*shRNA-treated microglial cells. Scale bar, 5 μm. **b** qPCR analysis of mRNA expression of *C1qa, C3, CD11b,* and *FcγRIIb* in microglial cells with or without the addition of corresponding shRNA (*n* = 3 independent experiments). **c** Quantification of engulfed PSD95 puncta in microglial cells in **a** (*n* = 6 biological replicates over three independent experiments). **d** Immunolabeling of engulfed PSD95 (red) within Iba-1^+^ (green) microglial cells in neuron-microglia cocultures pretreated with A16 or Fab16 before the addition of AβOs. Scale bar, 5 μm. **e** Quantification of PSD95 puncta engulfed by Iba-1^+^ microglial cells in **d** (*n* = 6 biological replicates over three independent experiments). Data are expressed as mean ± s.e.m. and were analyzed by one-way ANOVA with Tukey’s test. ***P* < 0.01, *****P* < 0.0001; ns, not significant.


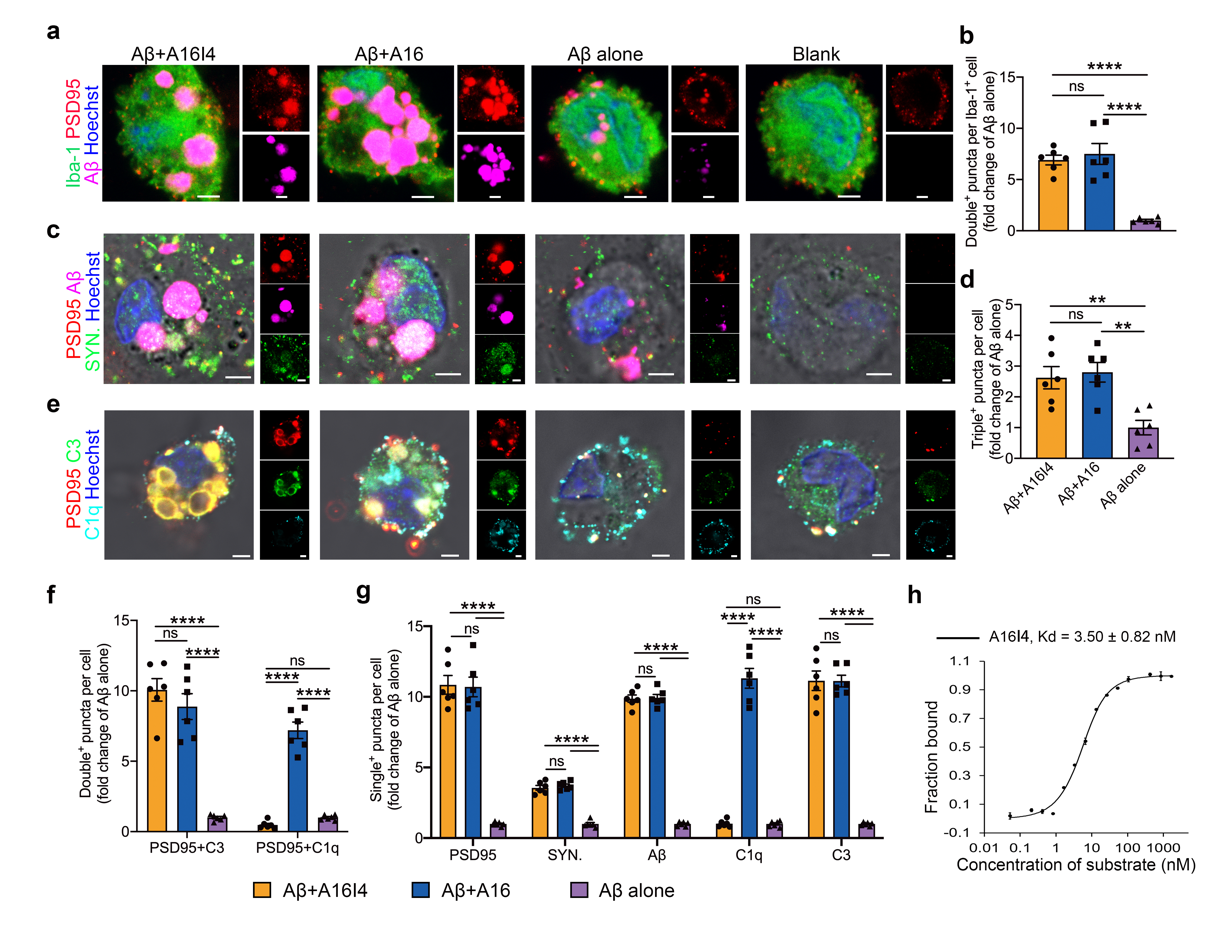


**Supplementary Fig. 24. IgG4 antibody A16I4 induces microglial engulfment of synapses *in vitro*.** **a** Immunolabeling of PSD95 (red) and Aβ (magenta) in Iba-1^+^ (green) microglial cells in neuron-microglia cocultures treated with A16I4 or A16 in the presence of AβOs. Scale bar, 2.5 μm. **b** Quantification of colocalized PSD95 and Aβ puncta per Iba-1^+^ microglia cell in **a** (*n* = 6). **c** Immunolabeling of PSD95 (red), Aβ (magenta), and synaptophysin (green) in microglial cells in cocultures. Scale bar, 2.5 μm. **d** Quantification of colocalized PSD95, Aβ, and synaptophysin puncta per cell in **c** (*n* = 6). **e** Immunolabeling of PSD95 (red), C3 (green), and C1q (cyan) in microglial cells in cocultures. Scale bar, 2.5 μm. **f** Quantification of colocalized PSD95 and C3 puncta, or PSD95 and C1q puncta, per cell in **e** (*n* = 6). **g** Quantification of PSD95, synaptophysin, Aβ, C1q, and C3 puncta per cell **(***n* = 6). **h** The binding affinity of A16I4 with Aβ measured using microscale thermophoresis (*n* = 3 independent experiments). All experiments in **b**, **d**, **f**, and **g** were performed as biological replicates over three independent experiments. Data are expressed as mean ± s.e.m. and were analyzed by one-way ANOVA with Tukey’s test. ***P* < 0.01, *****P* < 0.0001; ns, not significant.

**
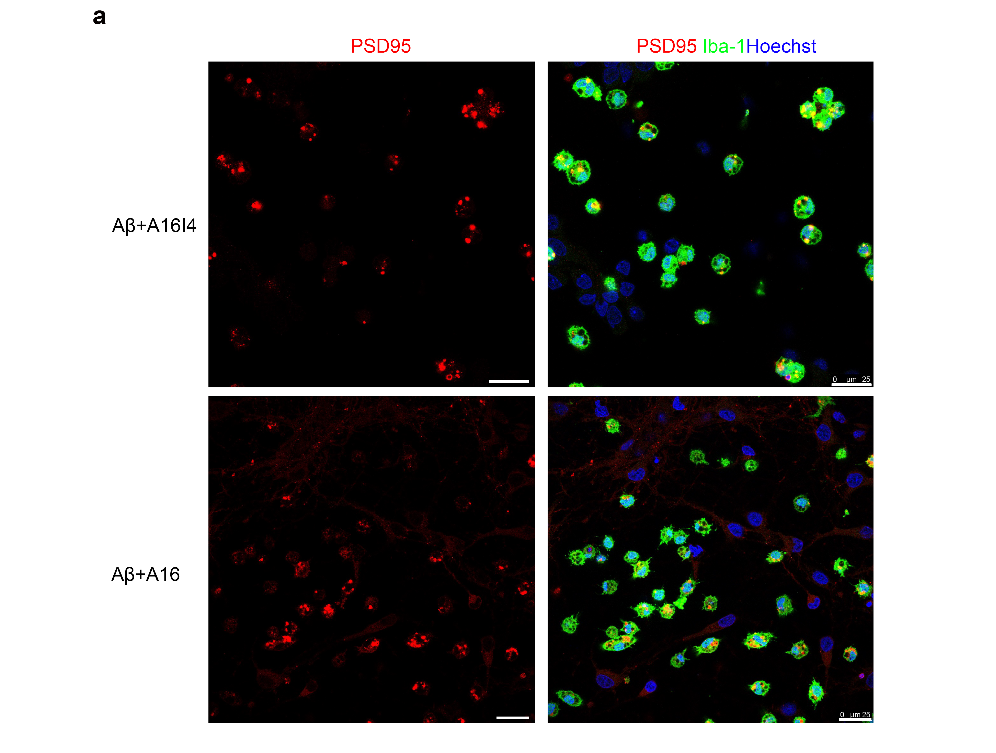
**

**Supplementary Fig. 25. Both IgG4 antibody A16I4 and IgG1 antibody A16 induce microglial synapse engulfment *in vitro*. a** Immunolabeling of PSD95 (red) and Iba-1 (green) in neuron-microglia cocultures treated with A16I4 or A16 in the presence of AβOs. Scale bars, 25 μm.


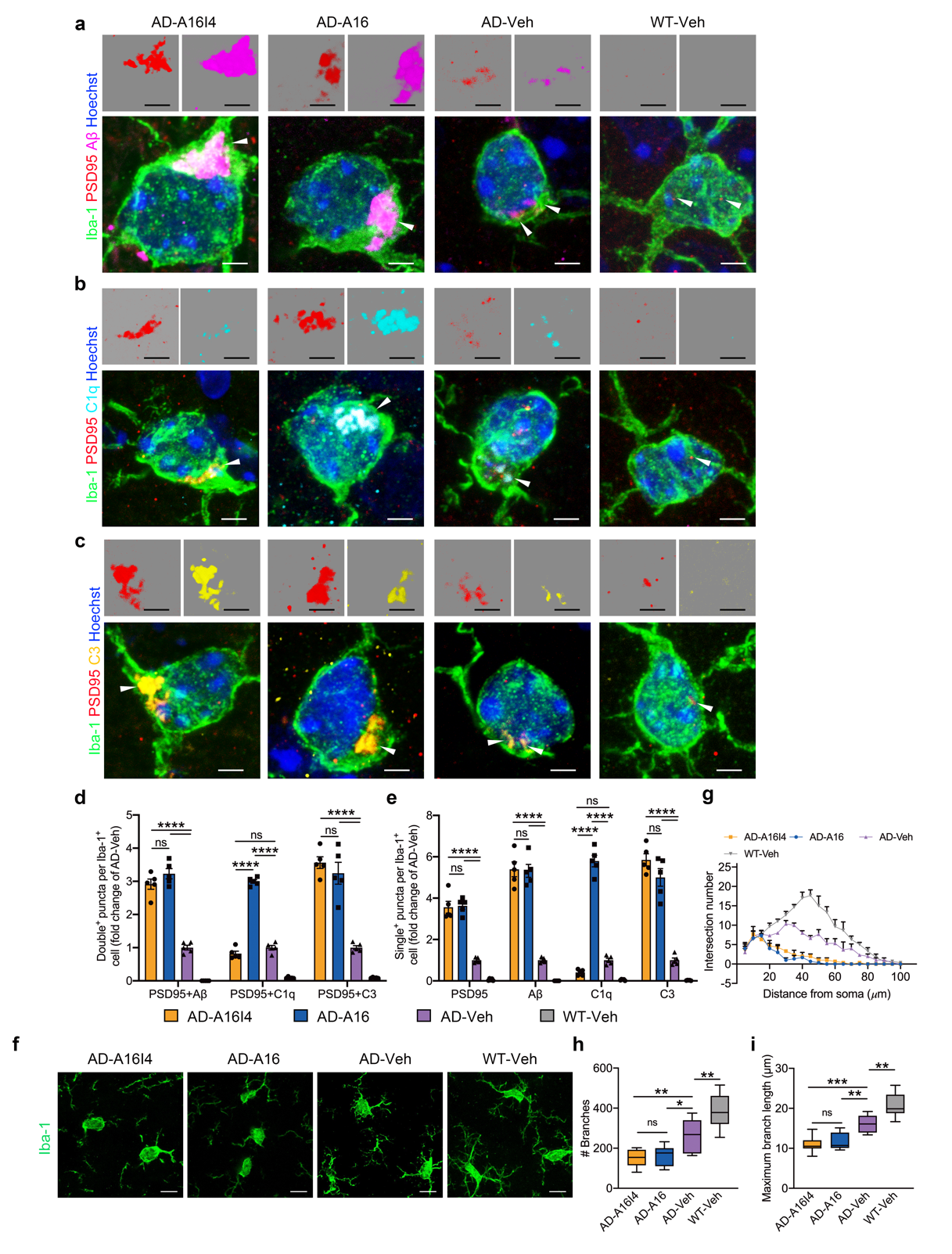


**Supplementary Fig. 26. IgG4 antibody A16I4 induces microglial engulfment of synapses in 6-month-old APP/PS1 mice. a** Representative images of the engulfed PSD95 (red) and Aβ (magenta) puncta within Iba-1^+^ (green) microglial cells in the brains of APP/PS1 or WT mice treated with A16I4, A16, or PBS, at 48 h post-injection. Scale bar, 3 μm. **b** Representative images of the engulfed PSD95 (red) and C1q (cyan) puncta within Iba-1^+^ (green) microglial cells in the brains of APP/PS1 or WT mice treated with A16I4, A16, or PBS, at 48 h post-injection. Scale bar, 3 μm. **c** Representative images of the engulfed PSD95 (red) and C3 (yellow) puncta within Iba-1^+^(green) microglial cells in the brains of APP/PS1 or WT mice treated with A16I4, A16, or PBS, at 48 h post-injection. Scale bar, 3 μm. **d** Quantification of colocalized PSD95 and Aβ puncta, PSD95 and C1q puncta, and PSD95 and C3 puncta, per Iba-1^+^ microglial cell (*n* = 5 mice). **e** Quantification of PSD95, Aβ, C1q, and C3 puncta per Iba-1^+^ microglial cell (*n* = 5 mice). **f** Representative images of Iba-1^+^(green) microglial cells in the brains of APP/PS1 or WT mice treated with A16I4, A16, or PBS, at 48 h post-injection. Scale bar, 10 μm. **g-i** Sholl analysis (**g**) and Skeleton analysis (**h**, **i**) of microglial cells in **f** (*n* = 5 mice). Arrows indicate the engulfed inputs in microglia shown in **a, b**, and **c.** Data are expressed as mean ± s.e.m. (**d, e, g**) or shown as boxplots (**h, i**). For boxplots, the central band displays the median, the boxes depict values between lower and upper quartile, and the whiskers represent the minimum and maximum values. One-way ANOVA with Tukey’s test was performed to determine significance. **P* < 0.05, ***P* < 0.01, ****P* < 0.001, *****P* < 0.0001; ns, not significant.


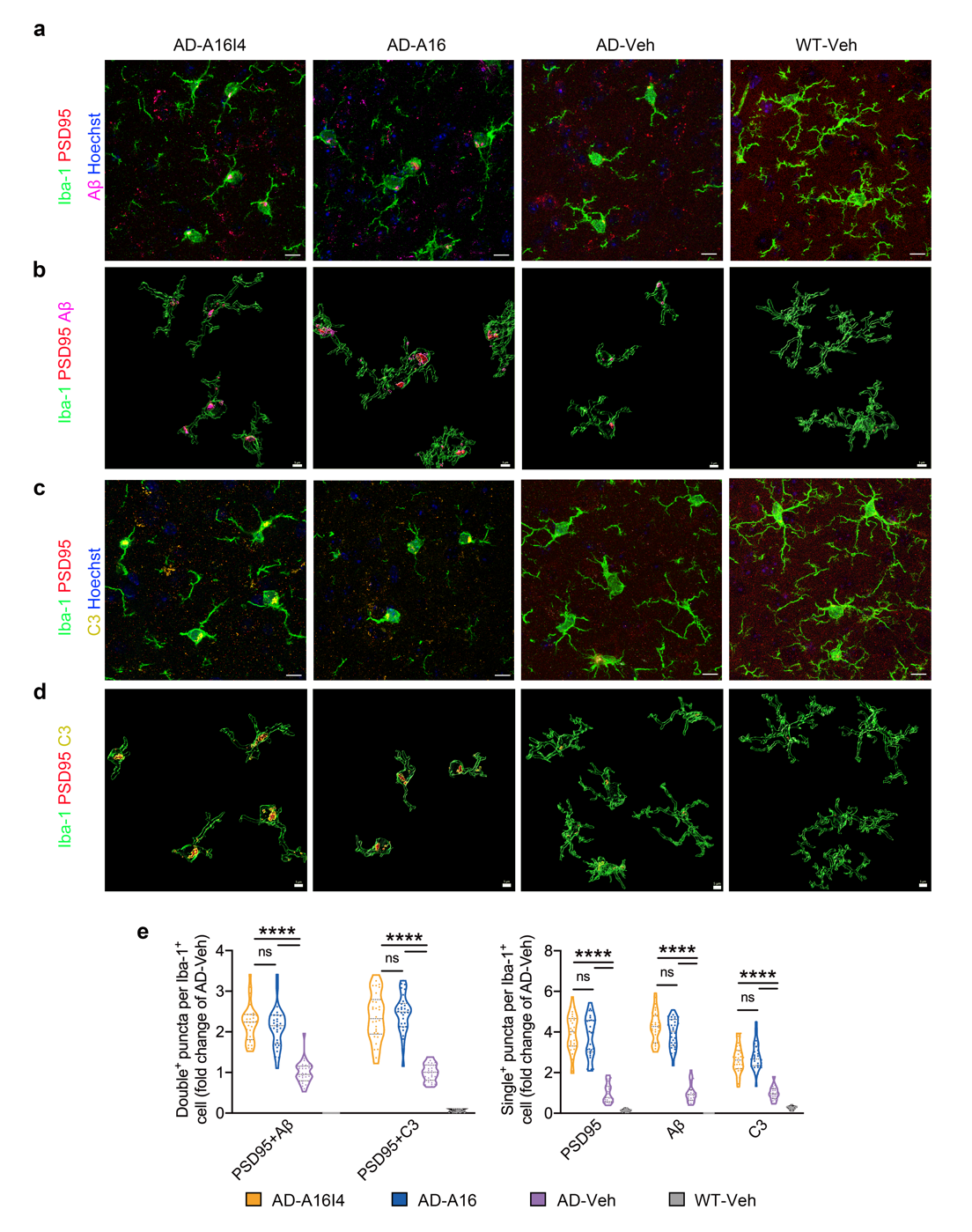


**Supplementary Fig. 27. IgG4 antibody A16I4 induces microglial synapse phagocytosis in 6-month-old APP/PS1 mice. a** Representative images of the engulfed PSD95 (red) and Aβ (magenta) puncta within Iba-1^+^ (green) microglial cells in the brains of APP/PS1 or WT mice treated with A16I4, A16, or PBS, at 48 h post-injection. Scale bar, 10 μm. **b** 3D surface rendering of Iba-1^+^ microglia (green) containing engulfed PSD95 (red) and Aβ (magenta) puncta in **a**. Scale bar, 5 μm. **c** Representative images of the engulfed PSD95 (red) and C3 (yellow) puncta within Iba-1^+^ (green) microglial cells in the brains of APP/PS1 or WT mice treated with A16I4, A16, or PBS, at 48 h post-injection. Scale bar, 10 μm. **d** 3D surface rendering of Iba-1^+^ microglia (green) containing engulfed PSD95 (red) and C3 (yellow) puncta in **c**. Scale bar, 5 μm. **e** Quantification of colocalized PSD95 and Aβ puncta, PSD95 and C3 puncta, and PSD95, Aβ and C3 puncta, per Iba-1^+^ microglial cell (*n* = 30 microglial cells from 5 mice). Data are displayed as violin plots and were analyzed by one-way ANOVA with Tukey’s test. Dotted lines represent medians and interquartile ranges. *****P* < 0.0001; ns, not significant.


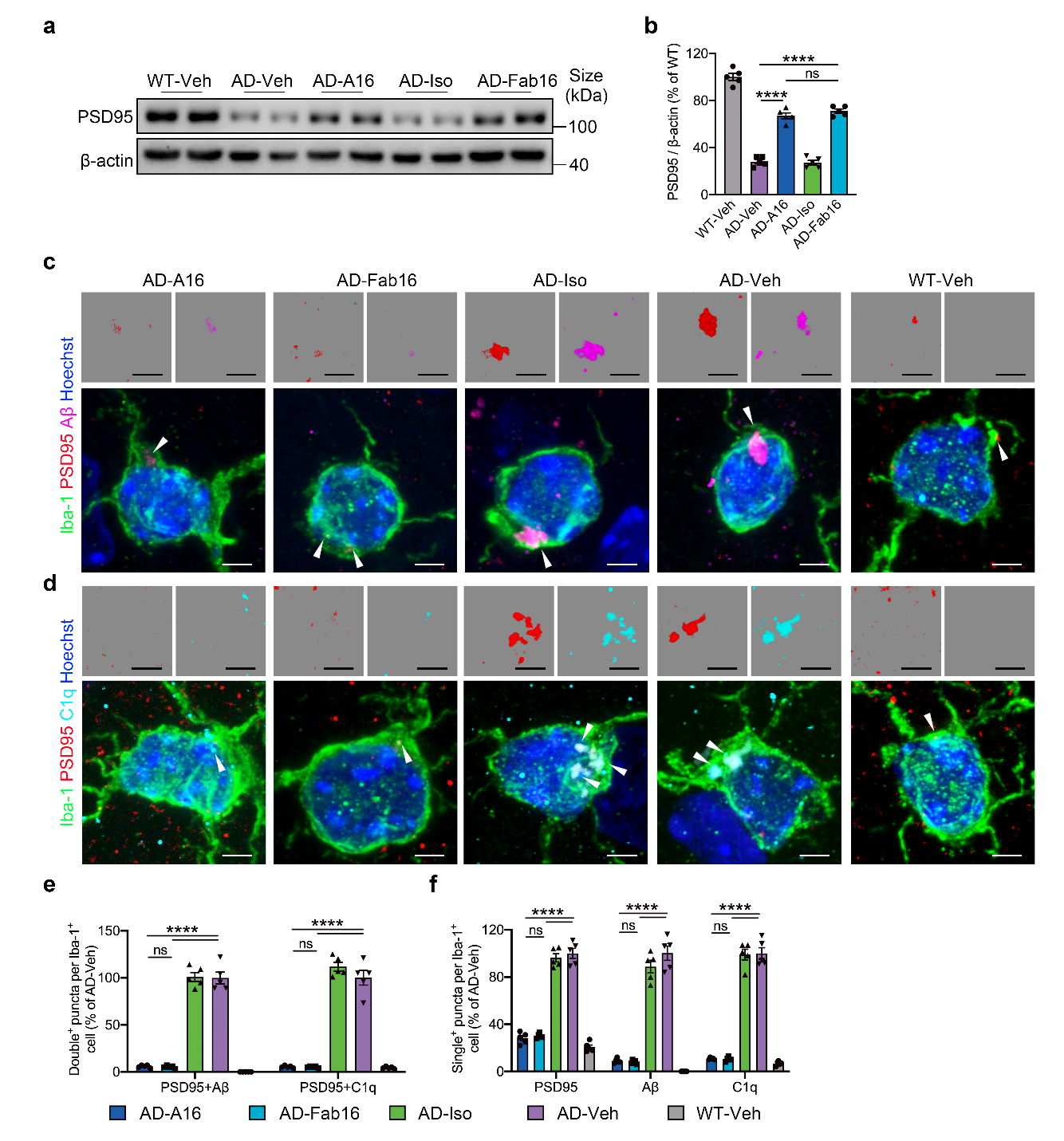


**Supplementary Fig. 28. Full effector antibody A16 prevents synapse loss in APP/PS1 mice at 2-week post 2^nd^ antibody treatment. a** Western blot analysis of PSD95 in the brains of APP/PS1 or WT mice treated with A16, Fab16, isotype control antibody, or PBS, at 2-week post 2^nd^ antibody treatment. **b** Quantitation of PSD95 expression in **a** (*n* = 5 mice). **c** Representative images of the engulfed PSD95 (red) and Aβ (magenta) puncta within Iba-1^+^ (green) microglial cells in the brains of APP/PS1 or WT mice treated with A16, Fab16, isotype control antibody, or PBS, at 2-week post 2^nd^ antibody treatment. Scale bar, 3 μm. **d** Representative images of the engulfed PSD95 (red) and C1q (cyan) puncta within Iba-1^+^ (green) microglial cells in the brains of APP/PS1 or WT mice treated with A16, Fab16, isotype control antibody, or PBS, at 2-week post 2^nd^ antibody treatment. Scale bar, 3 μm. **e** Quantification of the colocalized PSD95 and Aβ puncta, PSD95 and C1q puncta per Iba-1^+^ microglial cell (*n* = 5 mice). **f** Quantification of PSD95, Aβ, and C1q puncta per Iba-1^+^ microglial cell (*n* = 5 mice). Arrows indicate the engulfed inputs in microglia in **c** and **d**. Data are expressed as mean ± s.e.m. and were analyzed by one-way ANOVA with Tukey’s test. *****P* < 0.0001; ns, not significant.

**
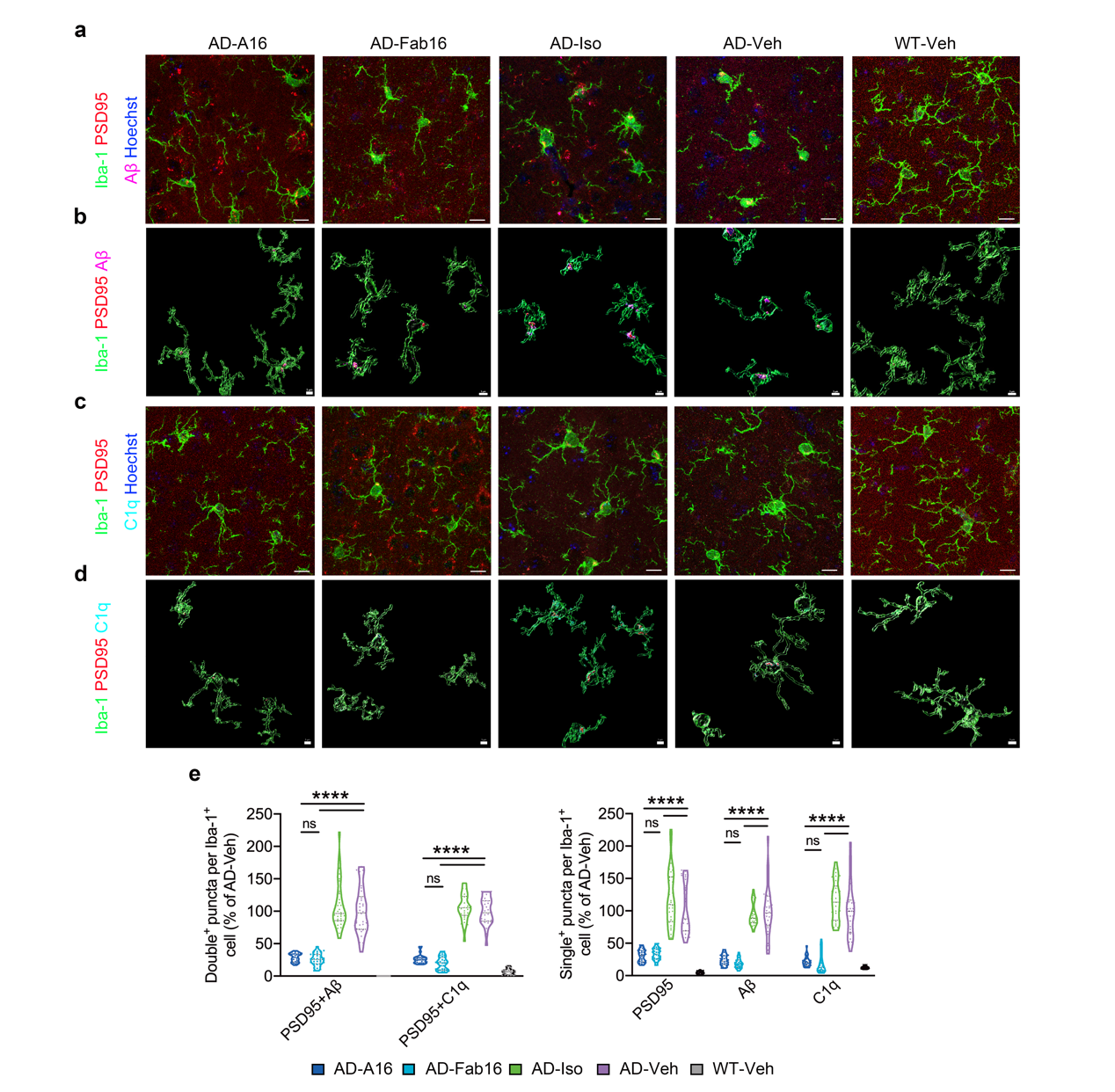
**

**Supplementary Fig. 29. Full effector antibody A16 fails to induce microglial synapse engulfment in APP/PS1 mice at 2-week post 2^nd^ antibody treatment. a** Representative images of the engulfed PSD95 (red) and Aβ (magenta) puncta within Iba-1^+^ (green) microglial cells in the brains of APP/PS1 or WT mice treated with A16, Fab16, isotype control antibody or PBS, at 2-week post 2^nd^ antibody treatment. Scale bar, 10 μm. **b** 3D surface rendering of Iba-1^+^ microglia (green) containing engulfed PSD95 (red) and Aβ (magenta) puncta in **a**. Scale bar, 5 μm. **c** Representative images of the engulfed PSD95 (red) and C1q (cyan) puncta within Iba-1^+^ (green) microglial cells in the brains of APP/PS1 or WT mice treated with A16, Fab16, isotype control antibody or PBS, at 2-week post 2^nd^ antibody treatment. Scale bar, 10 μm. **d** 3D surface rendering of Iba-1^+^ microglia (green) containing engulfed PSD95 (red) and C1q (cyan) puncta in **c**. Scale bar, 5 μm. **e** Quantification of colocalized PSD95 and Aβ puncta, PSD95 and C1q puncta, and PSD95, Aβ and C1q puncta, per Iba-1^+^ microglial cell (*n* = 30 microglia cells from 5 mice). Data are displayed as violin plots and were analyzed by one-way ANOVA with Tukey’s test. Dotted lines represent medians and interquartile ranges. *****P* < 0.0001; ns, not significant.


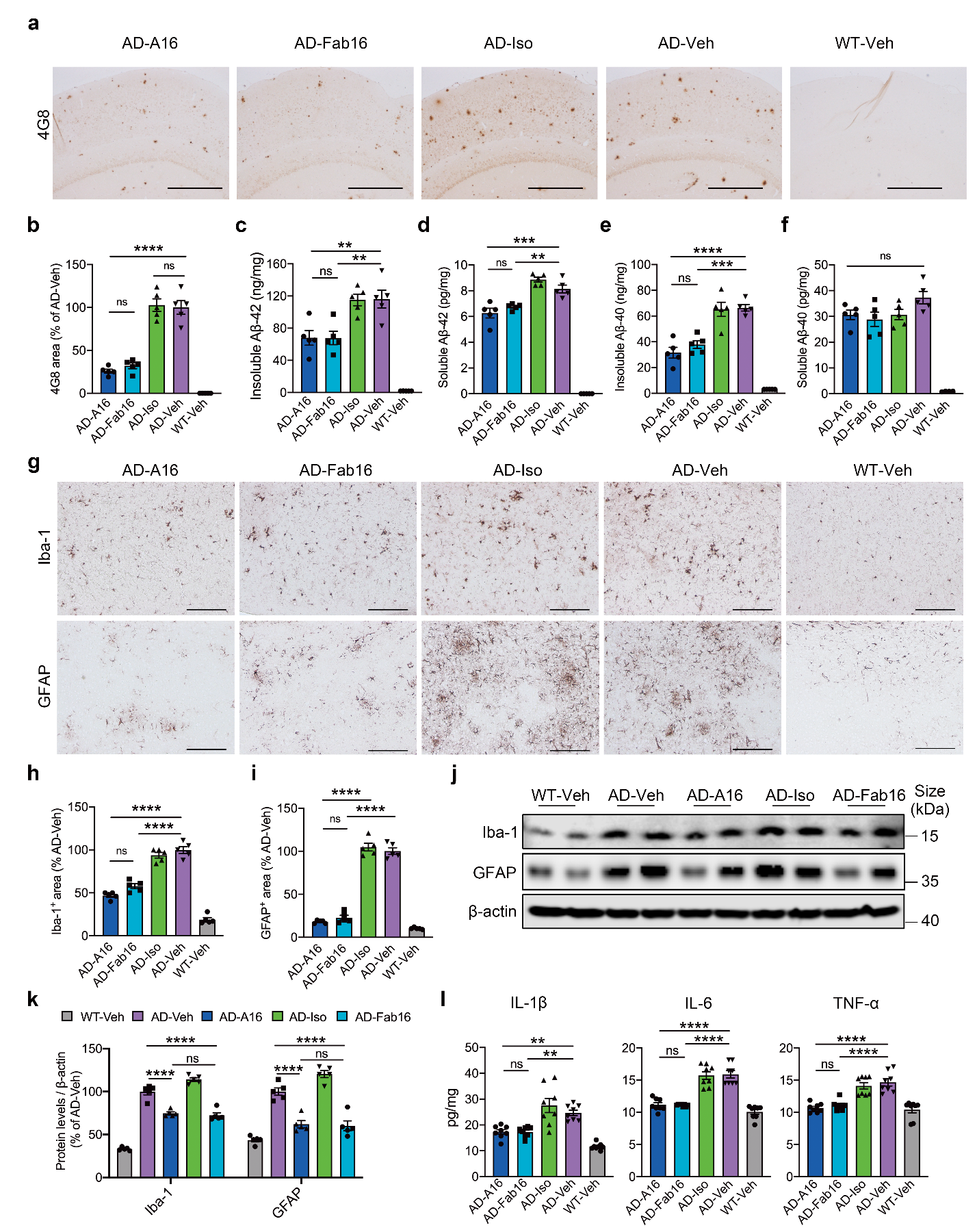


**Supplementary Fig. 30. Full effector antibody A16 reduces Aβ burden and neuroinflammation in APP/PS1 mice at 2-week post 2^nd^ antibody treatment. a** Detection of Aβ plaques by 4G8 immunolabeling in the brains of APP/PS1 or WT mice treated with A16, Fab16, isotype control antibody, or PBS, at 2-week post 2^nd^ antibody treatment. Scale bar, 500 μm. **b** Quantification of 4G8-labeled area in **a** (*n* = 5 mice). **c**-**f** Levels of insoluble Aβ42 (**c**), soluble Aβ42 (**d**), insoluble Aβ40 (**e**), and soluble Aβ40 (**f**) in the brain lysates of APP/PS1 or WT mice treated with A16, Fab16, isotype control antibody, or PBS, at 2-week post 2^nd^ antibody treatment (*n* = 5 mice). **g** Immunolabeling for Iba-1 and GFAP in the brains of APP/PS1 or WT mice treated with A16, Fab16, isotype control antibody, or PBS, at 2-week post 2^nd^ antibody treatment. Scale bar, 200 μm. **h** Quantification of Iba-1-labeled area in **g** (*n* = 5 mice). **i** Quantification of GFAP-labeled area in **g** (*n* = 5 mice). **j** Western blot analysis of the expression of Iba-1 and GFAP in the brains of APP/PS1 or WT mice treated with A16, Fab16, isotype control antibody, or PBS, at 2-week post 2^nd^ antibody treatment. **k** Quantitation of the expression of Iba-1 and GFAP in **j** (*n* = 5 mice). **l** The levels of IL-1β, IL-6, and TNF-α in the brain lysates of APP/PS1 or WT mice treated with A16, Fab16, isotype control antibody, or PBS, at 2-week post 2^nd^ antibody treatment (*n* = 8 mice). Data are expressed as mean ± s.e.m. and were analyzed by one-way ANOVA with Tukey’s test. ***P* < 0.01, ****P* < 0.001, *****P* < 0.0001; ns, not significant.


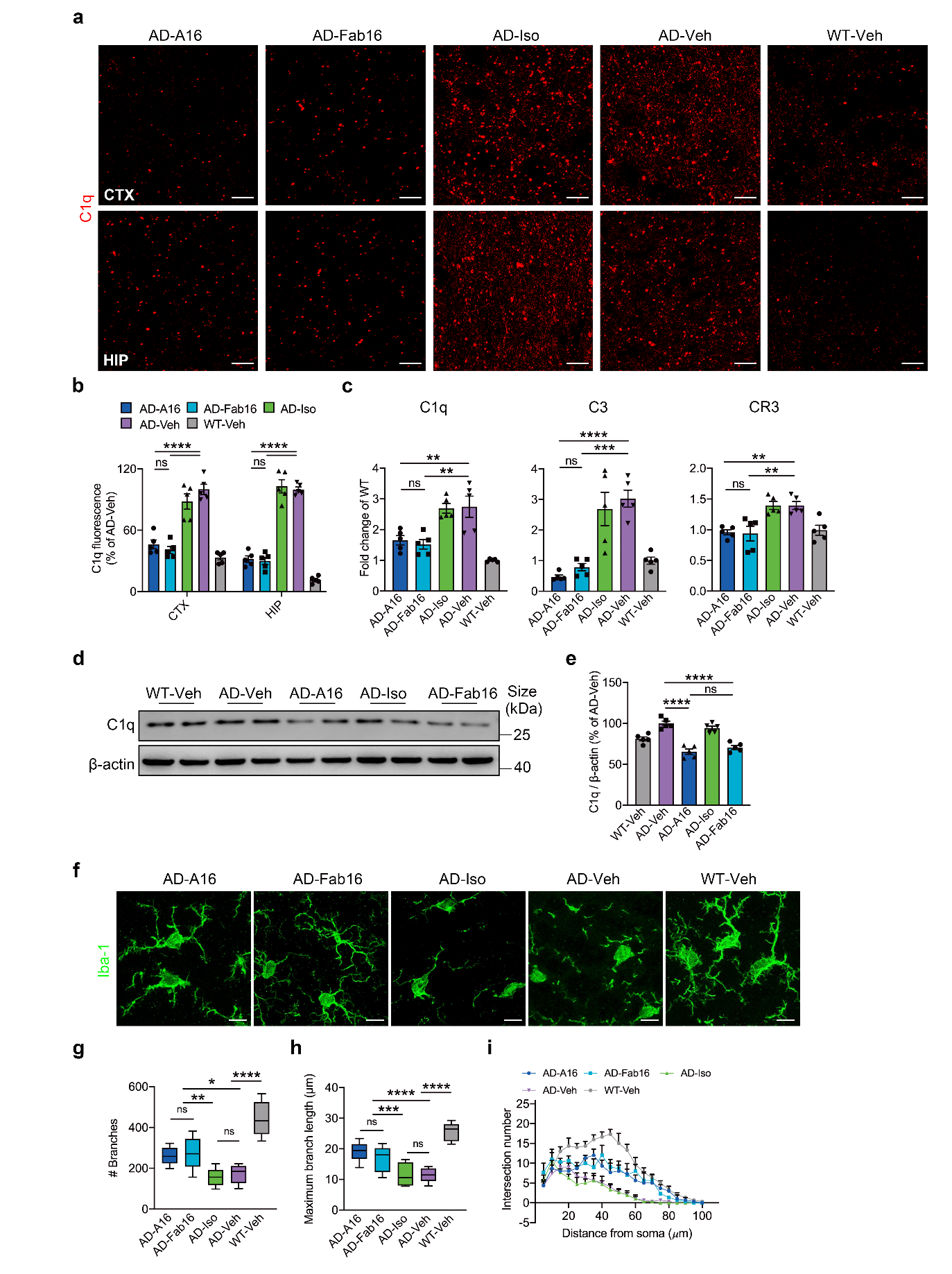


**Supplementary Fig. 31. Full effector antibody A16 reduces C1q levels in the brains of APP/PS1 mice at 2-week post 2^nd^ antibody treatment. a** Immunolabeling for C1q in the cortex and hippocampus of APP/PS1 or WT mice treated with A16, Fab16, isotype control antibody, or PBS, at 2-week post 2^nd^ antibody treatment. Scale bar, 10 μm. **b** Quantification of C1q immunofluorescence intensity in **a** (*n* = 5 mice). **c** qPCR analysis of *C1q, C3,* and *CR3* mRNA expression in the brains of APP/PS1 or WT mice treated with A16, Fab16, isotype control antibody, or PBS, at 2-week post 2^nd^ antibody treatment (*n* = 5 mice). **d** Western blot analysis of C1q expression in the brains of APP/PS1 or WT mice treated with A16, Fab16, isotype control antibody, or PBS, at 2-week post 2^nd^ antibody treatment. **e** Quantitation of C1q expression in **d** (*n* = 5 mice). **f** Representative images of Iba-1^+^ (green) microglial cells in the brains of APP/PS1 or WT mice treated with A16, Fab16, isotype control antibody, or PBS, at 2-week post 2^nd^ antibody treatment. Scale bar, 8 μm. **g-i** Skeleton analysis (**g**, **h**) and Sholl analysis (**i**)of microglial cells in **f** (*n* = 5 mice). Data are expressed as mean ± s.e.m. (**b, c, e, i**) or shown as boxplots (**g**, **h**). For boxplots, the central band displays the median, the boxes depict values between lower and upper quartile, and the whiskers represent the minimum and maximum values. One-way ANOVA with Tukey’s test was performed to determine significance. **P* < 0.05, ***P* < 0.01, ****P* < 0.001, *****P* < 0.0001; ns, not significant.


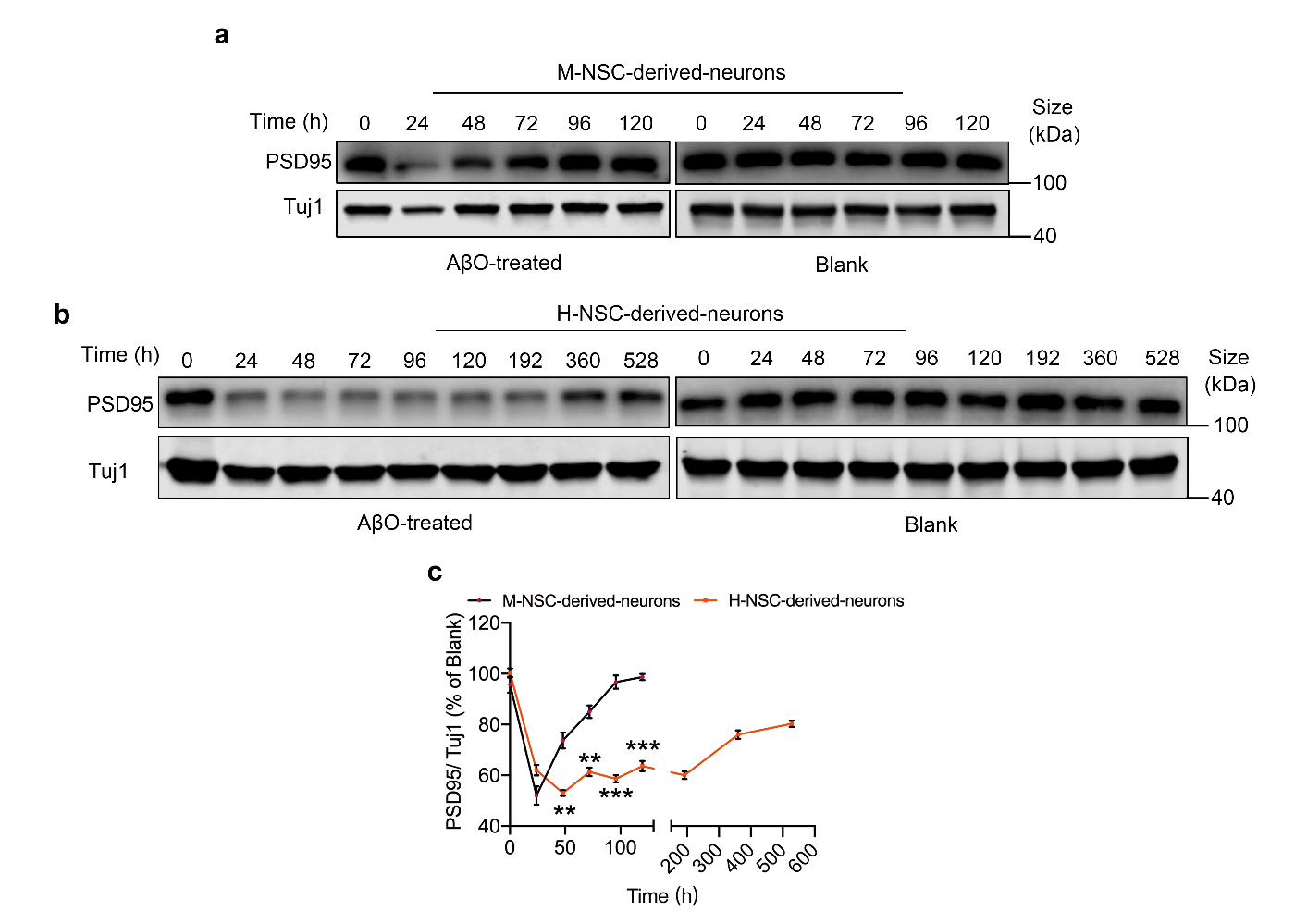


**Supplementary Fig. 32. The rate of synaptogenesis in humans is much lower than that in mice. a** The neuronal cultures derived from mouse neuronal stem cells were treated with 500 nM AβOs for 24 h, then the medium was replaced to fresh medium and the cultures were further incubated for indicated hours. PSD95 levels in cultured neurons were detected using western-blotting. **b** The neuronal cultures derived from human neuronal stem cells were treated with 500 nM AβOs for 24 h, then the medium was replaced to fresh medium and the cultures were further incubated for indicated hours. PSD95 levels in cultured neurons were detected using western-blotting. **c** Quantitation of PSD95 immunoblots in a and b (n = 3 independent experiments). Data are expressed as mean ± s.e.m. and were analyzed by two-tailed Student’s t test. ***P* < 0.01, ****P* < 0.001.

**Supplementary Movie 1. Full effector antibody A16 robustly mediates synapse loss in APP/PS1 mice at 48 h post-treatment.**

Immunolabeling of PSD95 (red) in the brains of APP/PS1 or WT mice treated with A16, Fab16, isotype control antibody, or PBS, at 48 h post-injection.

**Supplementary Movie 2. Full effector antibody A16 significantly promotes microglial engulfment of synapses in APP/PS1 mice at 48 h post-treatment.**

The engulfed PSD95 (red) puncta within Iba-1^+^ (green) microglial cells in the brains of APP/PS1 or WT mice treated with A16, Fab16, isotype control antibody, or PBS, at 48 h post-injection.
